# Characterization of a C-methyltransferase from *Streptomyces griseoviridis* – crystal structure, mechanism, and substrate scope†

**DOI:** 10.1039/d4sc07300b

**Published:** 2025-01-30

**Authors:** Mona Haase, Oliver H. Weiergräber, Benoit David, Elias L. Pfirmann, Beatrix Paschold, Holger Gohlke, Jörg Pietruszka

**Affiliations:** a Heinrich Heine University Düsseldorf in Forschungszentrum Jülich, Institute for Bioorganic Chemistry & Bioeconomy Science Center (BioSC) 52426 Jülich Germany j.pietruszka@fz-juelich.de; b Forschungszentrum Jülich, Institute of Biological Information Processing (IBI-7: Structural Biochemistry) 52425 Jülich Germany o.h.weiergraeber@fz-juelich.de; c Forschungszentrum Jülich, Institute of Bio- and Geosciences (IBG-4: Bioinformatics) 52426 Jülich Germany; d Forschungszentrum Jülich, Institute of Bio- and Geosciences (IBG-1: Bioorganic Chemistry) & Bioeconomy Science Center (BioSC) 52426 Jülich Germany gohlke@uni-duesseldorf.de; e Heinrich Heine University Düsseldorf, Institute for Pharmaceutical and Medicinal Chemistry & Bioeconomy Science Center (BioSC) 40225 Düsseldorf Germany

## Abstract

Despite the presence of pyrroloindoles in many natural products with diverse biological activities, their synthesis remains challenging in terms of stereoselectivity, especially with respect to methylation at the indole C3 position. In the present study, the pyrroloindole motif in tryptophan-based diketopiperazines (DKPs) is synthesized using the SAM-dependent methyltransferase SgMT from *Streptomyces griseoviridis*. The three-dimensional structure of this indole C3-methyltransferase was determined by X-ray crystallography, providing insights into the enzyme. The complex active site was explored by site-directed mutagenesis, highlighting an intriguing network of tyrosine side chains that is involved in catalytic activity. The enzyme's precise substrate requirements were characterized using a broad panel of methylation educts, while molecular docking and molecular dynamics simulations revealed the catalytic binding mode of the cyclo-(*ll*)-ditryptophan substrate. This study provides an in-depth account of the structure and catalytic properties of SgMT, which may apply to other diketopiperazine-targeting indole C3-methyltransferases, thus paving the way for their optimization as biocatalysts.

## Abstract 2

This study elucidates the structure, catalytic mechanism, and substrate selectivity of the SAM-dependent methyltransferase from *Streptomyces griseoviridis*, which catalyses the synthesis of pyrroloindole motifs in tryptophan-based diketopiperazines.

## Graphical abstract

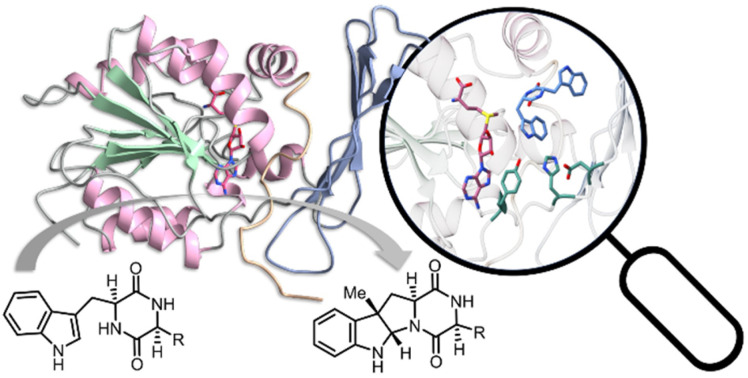

## Introduction

**Fig. 1:**
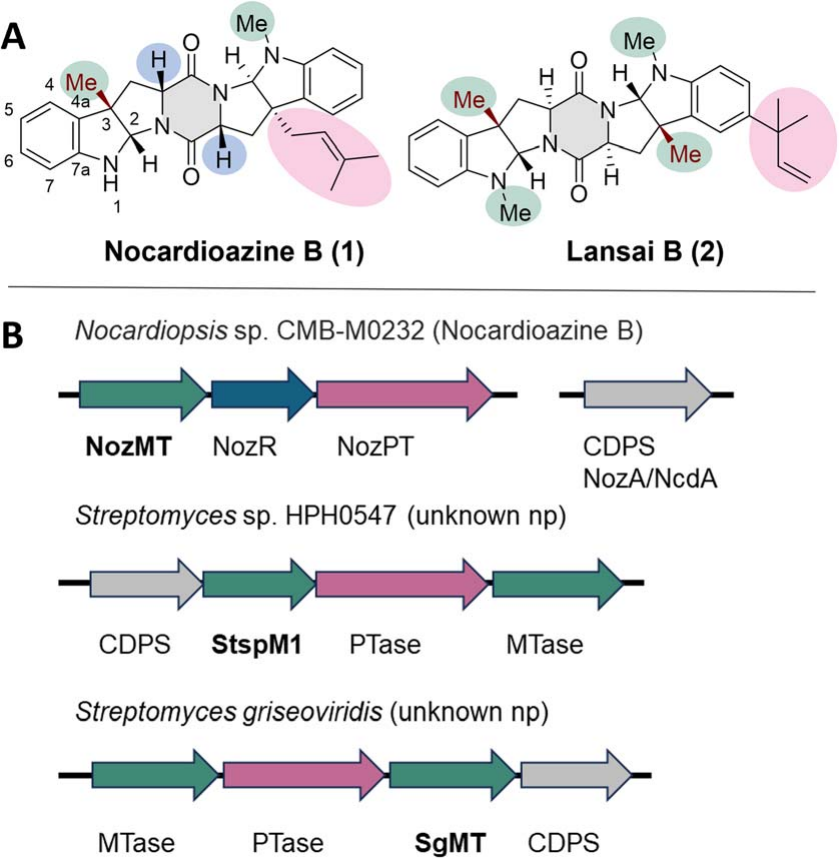
(A) Selected natural products (np), which are tryptophan-based diketopiperazines with pharmaceutical interest: nocardioazine B and lansai B. The chemical groups and structural motifs which are important for their biosynthesis are highlighted in the corresponding colour of the genes in the gene clusters (B). (B) Gene clusters containing the genes for the C3-indole methyltransferases (MTases) NozMT, StspM1 and SgMT. MTases are highlighted in green, prenyltransferases (PTases) in pink, the isomerase in blue and the cyclodipeptide synthases (CDPSs) in grey. The exact biosynthetic pathway of lansai B is not known.

2,5-Diketopiperazines (DKPs) are cyclodipeptides obtained by condensing two α-amino acids to form a semirigid six-membered ring as core structure.^1,2^ Numerous natural products containing the DKP ring have been isolated and investigated with respect to their biosynthesis.^3^ They have attracted attention because many of these compounds exhibit diverse biological activities ranging from functions as cell cycle inhibitors^4^ over DNA-binding agents^5^ to antivirals.^6^ This broad spectrum of bioactivity is due to multiple H-bond acceptor and donor functionalities, complemented by a hydrophobic or aromatic character, depending on the substituents of the DKP scaffold, thus enabling high affinity and selectivity binding to a variety of receptors.^1^ The most common DKP natural products are based on the cyclic condensation of tryptophan and proline amino acids. In cyclic dipeptides containing proline, the DKP ring is fused to a cyclic five-membered ring on either side, causing rigid constraints.^7^ Tryptophan-based DKPs are often processed further, yielding a pyrroloindole (hexahydropyrrolo[2,3-*b*]indole) ring system like in nocardioazine B (1) or lansai B (2), which leads as well to an increased rigidity (Fig. 1A).^8,9^

The synthesis of the pyrroloindole structural motif remains challenging with respect to stereoselectivity.^10^ In nature, several classes of enzymes such as methyl- and prenyltransferases are used for selective *C*-alkylation and to generate this rigid tricyclic structure by the addition of a substituent at the C3-position of the indole, followed by dearomatization and an intramolecular cyclization step.^9,11,12^ In particular for methylation at the C3 position, there is no direct method that achieves the same selectivity as the enzyme-catalysed reaction.^11,13^

For this reason, indole C3-methyltransferases have attracted considerable interest from research groups, including those of Viswanathan, Zhu, Lane, and ours, in recent years.^11,14–16^ The crystal structure of a C3-methyltransfease from *Streptomyces griseofuscus* (PsmD), which is involved in the biosynthesis of the acetylcholinesterase inhibitor physostigmine, provided important insights into this enzyme class for the formation of small pyrroloindole containing products.^15^ With regard to diketopiperazine substrates, two methyltransferases, StspM1 from *Streptomyces* sp. HPH0547 and NozMT from *Nocardiopsis* sp. CMB-M0232, have been characterized, but their catalytic mechanisms are not fully understood as the proposed catalytic residues have not yet been completely validated through mutagenesis studies.^11,16^

In this study, the newly discovered indole C3-methyltransferase SgMT from *Streptomyces griseoviridis* was subjected to an in-depth investigation including a biochemically characterization, 3D structure determination, and functional assignment of its catalytic residues. Together with molecular simulation data, which was used to unravel the *ll*-cycloditryptophan (cWW) binding mode in SgMT, this work provides insights into the catalytic mechanism of this enzyme.

## Results and discussion

### Analysis of the gene cluster

A sequence alignment showed that SgMT is homologous to both NozMT^16^ and StspM1 (ref. 11) with 45.6% and 80% sequence identity, respectively. NozMT is a bifunctional methyltransferase catalysing both *N*- and *C*-methylations in nocardioazine B (1) biosynthesis (Fig. S1†).^16^ The associated gene cluster, *noz2*, includes the gene for NozMT, along with genes for a prenyltransferase (NozPT) and an isomerase (NozR). The genes responsible for production of the CDPS (cyclodipeptide synthase) in this biosynthesis are located in a gene cluster (nozA/ncdA) separate from the *noz2* cluster encoding the methyltransferase NozMT (Fig. 1B).^16,17^ The methyltransferase StspM1 is encoded in a gene cluster with a CDPS, a prenyltransferase and a second methyltransferase.^11^ While the role of NozMT in nocardioazine biosynthesis has been confirmed, the final product of the biosynthetic pathway involving StspM1 remains unknown. Unlike the *noz2* cluster (Fig. 1B) neither the StspM1 nor the SgMT encoding gene clusters contain an isomerase, which is essential for nocardioazine B biosynthesis by transforming the *ll*-cWW (4a) into the *dd*-cWW (4b) prior to methylation (Fig. S1†). However, both gene clusters contain all the necessary components for the synthesis of a natural product like lansai B (1) (Fig. 1).

**Fig. 2:**
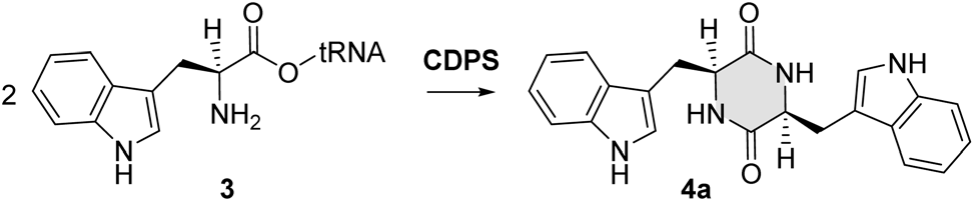
Reaction catalysed by a CDPS: the diketopiperazine motif (4a) is formed by condensing two l-tryptophanyl-tRNA (3).

Notably, the CDPS within the SgMT-containing gene cluster is annotated as a cyclo(l-leucyl-l-leucyl) synthase (GenBank: GGT26792.1). CDPSs catalyse the formation of DKP motifs using aminoacyl-tRNAs (aa-tRNAs).^18^ Their two binding sites contain consensus motifs that correspond to specific DKP profiles, enabling the prediction of the potential DKP products.^19^ For the CDPS in the SgMT gene cluster, the eight residues in the first binding site (residues 33, 35, 65, 67, 119, 185, 186, 200; AlbC numbering) and the seven residues in the second binding site (residues 152, 155, 156, 159, 204, 206, 207; AlbC numbering) were identified. The high sequence identity (>90%) of these motifs in the CDPS encoded by the SgMT gene cluster (SgCDPS) and the equivalent motifs in other characterized CDPSs suggests that SgCDPS likely forms the cWW DKP as a product, in contrast to the cLL which was annotated in GenBank (Fig. 2 and S2†).

### Characterization of SgMT

**Fig. 3:**
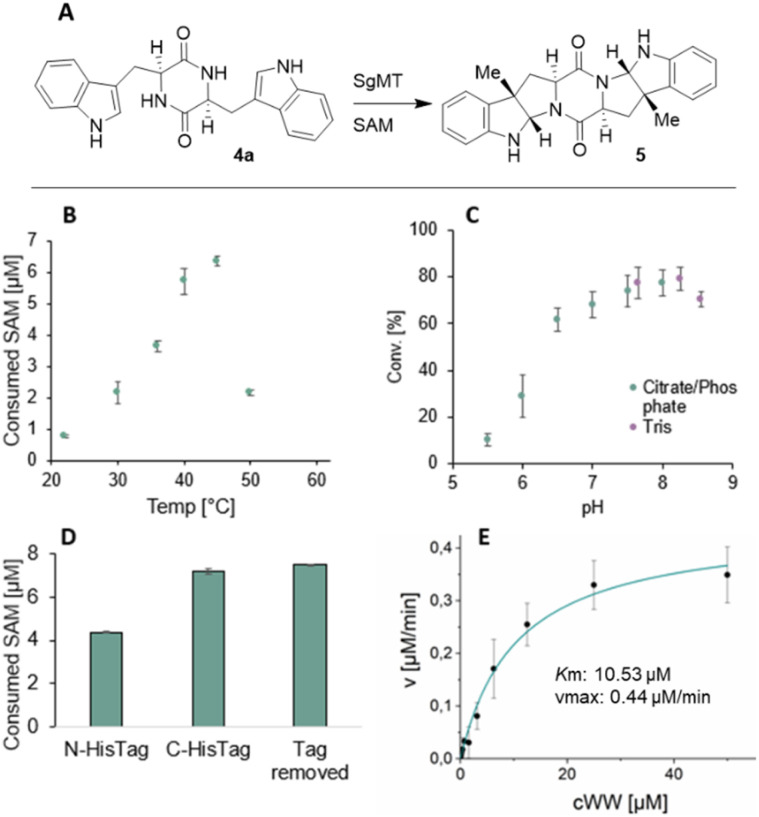
(A) C3-methylation of 4a catalysed by SgMT. (B)–(E) Biochemical characterization of SgMT: temperature screen (B), pH screen (C), influence of the His-tag on the enzyme activity (D) and Michaelis–Menten kinetics (E). Activities shown in (B), (D), and (E) were determined with the MTase Glo assay (Promega), those in (C) *via* HPLC.

Analysis of the gene cluster and the CDPS indicates that *ll*-cWW 4a is a potential substrate for the C3-indole methyltransferase SgMT, leading to the formation of the pyrroloindole product 5 (Fig. 3a). Consequently, this substrate was selected for further characterization of SgMT. The His-tagged enzyme was produced heterologously in *Escherichia coli* BL21 (DE3). After purification with Ni-NTA resin, about 40 milligrams of protein were recovered per gram wet cells, which is 25 times the amount obtained for the homologous methyltransferase StspM1.^11^ The SgMT protein is therefore highly produced in a soluble form, which is a valuable feature for synthetic utility.

The activity of the methyltransferase SgMT was determined *via* the MTase Glo assay fom Promega (unless otherwise specified), which detects the formation of *S*-adenosylhomocysteine (SAH) *via* a luminescence reaction.^20^ A reaction temperature screening was performed in the range of 22 °C to 50 °C, showing the highest conversion at 45 °C (Table S3†). For determining the optimal pH value, conversion of the substrate was measured *via* HPLC. The highest activity was detected between pH 7.5 and 8.2 (Table S4†). The temperature optimum and the optimal pH range of SgMT resemble previous findings for StspM1 (ref. 11) (45 °C, pH 6–8) and PsmD^15^ (45 °C, pH 6–9). SgMT was expressed and purified with a C- or N-terminal His-tag. The N-terminal His-tag was removed with thrombin after purification. The comparison of the three variants, namely the N-terminally tagged, the C-terminally tagged, and untagged proteins, showed a decrease in activity for the N-terminally tagged SgMT by 41% (Table S5†). The N-terminal helix of the structurally similar indole C3-methyltransferase PsmD is known to function as a lid that is crucial for catalysis.^15^ Therefore, a His-tag on this terminus could hinder the movement of the lid, resulting in a decreased activity. The C-terminally His-tagged enzyme was used for the enzyme characterization: the *K*_m_ and *v*_max_ values for processing *ll*-cWW were determined as 10.53 ± 2.01 μM and 0.44 ± 0.03 μM min^−1^, respectively. The *k*_cat_ (0.0025 s^−1^) is slightly lower than the one of StspM1 (0.0036 s^−1^) under the same reaction conditions (Table S6†).

**Fig. 4:**
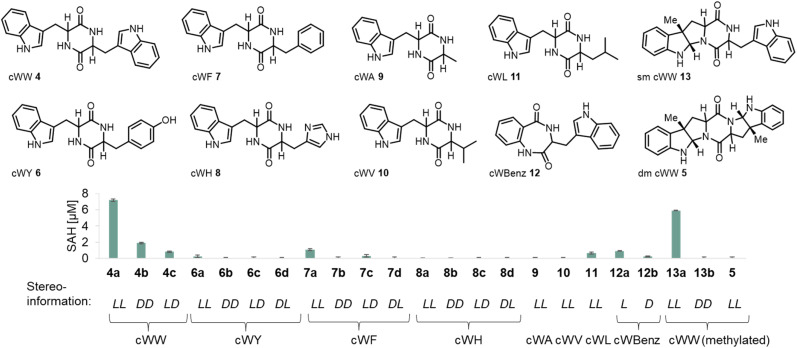
Substrate scope of SgMT. The activity of the methyltransferase was determined *via* the MTase Glo assay (Promega), which detects the consumption of SAM. The substrates are numbered, with letters indicating stereoisomers (**a**, *ll*; **b**, *dd*; **c**, *ld*; **d**, *dl*). The first stereodescriptor refers to the tryptophan and the second stereodescriptor to the respective second amino acid residue.

In total 18 different diketopiperazines 4–11 have been synthesized to investigate the substrate scope of the methyltransferase SgMT (Fig. 4 and Scheme S1†). To determine SgMT stereoselectivity towards DKPs with aromatic side chains, all possible isomer combinations were generated. Additionally, two benzodiazepinedione substrates 12 were tested (Scheme S2†). Benzodiazepines are known to bear different biological activities as anti-tubercular^21^ or anticancer agents.^22^ Natural products such as aszonalenin carry an alkyl chain at the C3-position of the indole forming the pyrroloindole motif, which is also present in nocardioazine.^8,23^

The *ll*-cWW substrate (4a) leads to the highest activity of the enzyme. Its isomer *dd*-cWW (4b) is accepted as well, but the activity decreases markedly. These reactions were repeated on an analytical scale, with products showing the same HPLC retention times as those reported for the enzyme StspM1.^11^ Characterization of the reaction products showed that *ll*-cWW (4a) is methylated on both indole moieties, whereas the *dd*-cWW (4b) is only methylated at one C3-position. The analytical data of the reaction products, including NMR, MS, and IR, align with the values reported in the literature.^11^ This observation is in line with the results of the Glo assay activity test, which showed conversion for the single methylated ll-cWW (13a) but not for the single methylated *dd*-cWW (13b) (Fig. 4). SgMT was additionally used in a preparative scale reaction (100 mg scale) as an immobilized enzyme for the conversion of 4a to 5 with a yield of 89% [conditions in ESI†]. *ld*-cWW (4c), *ll*-cWF (7a), *ld*-cWF (7c), ll-cWY (6a), ll-cWL (11) and the benzodiazepinedione derivatives 12a and 12b are all converted with low activity. The double C3-methylated *ll*-cWW (5) was also tested as a substrate to determine whether SgMT could catalyze an additional *N*-methylation of the pyrroloindole ring, as performed by the bifunctional enzyme NozMT.^16^ However, no conversion was detected (Fig. 4 and Table S3†).

### X-ray structure analysis of SgMT

**Fig. 5:**
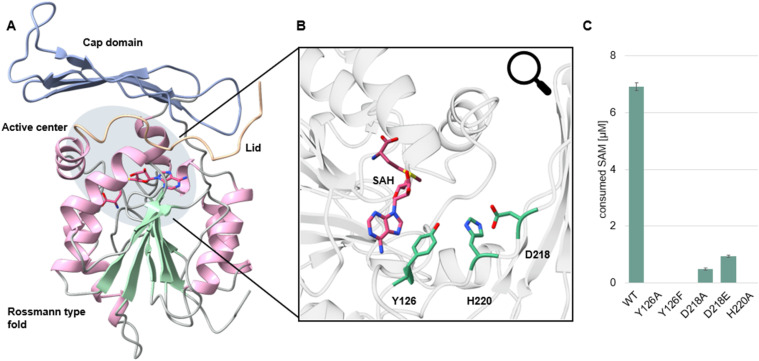
(A) Crystal structure of SgMT (PDB: 9GDJ). The Rossmann fold is displayed with α-helices and β-strands coloured pink and green, respectively. The cap domain is shown in blue, the lid in light orange. SAH is highlighted in dark salmon. (B) Active centre of SgMT. The catalytic triad (Y126, H220, and D218) is shown in green, the cofactor in dark salmon. (C) Results of the mutagensis study probing the catalytic residues of SgMT. Enzyme activities were measured with the MTase Glo assay (Promega).

In order to obtain more insights into the architecture and catalytic properties of SgMT, we crystallised the protein in the presence of the cofactor (using the non-methylated form, SAH) and determined its three-dimensional structure *via* X-ray crystallography (PDB: 9GDJ), using data extending to a diffraction limit (*d*_min_) of 1.47 Å. Crystals belong to space group *P*4_1_2_1_2 and contain three protein chains per asymmetric unit. Overall, the structure of SgMT closely resembles that of the related *C*-methyltransferase PsmD from *Streptomyces griseofuscus*.^15^ The major domain of the enzyme features a Rossmann-type α/β fold accommodating the cofactor at the bottom of a large solvent-filled cavity, which is further delimited by an all-β cap domain inserted between strand β5 and helix αE of the Rossmann domain, as well as an N-terminal extension completing the walls of the cavity (Fig. 5A). The similarity of SgMT and PsmD extends to their quaternary structure, which is established *via* two cap domains associating face-to-face at a right angle. While two out of three chains in the SgMT asymmetric unit form a non-crystallographic dimer, the third one engages in an analogous interaction with a symmetry-equivalent copy. Least-squares superposition of individual chains using residues 8–270 (considering the major conformation in case of alternates) yields average pairwise root-mean-square (RMS) distances of 1.36 Å for the 263 Cα atoms and 1.33 Å for all 1052 main-chain atoms, with major deviations being restricted to three short segments comprising residues 27–31, 203–212, and 164–168. Indeed, if these segments are excluded, average RMS distances reduce to 0.26 Å and 0.28 Å for 243 Cα atoms and 972 main-chain atoms, respectively, indicating that the overall impact of lattice interactions on the three-dimensional fold is very limited. The first divergent region (res. 27–31) comprises the end of helix αA′ and the proximal αA′-αA linker, thus connecting the Rossmann domain to the N-terminal extension, and is consistently observed with two alternate conformations in chains A and C, whereas chain B selects exactly one of these, suggesting a switch that is intrinsic to the protein structure (discussed below). The 164–168 region connects the short helix αC′ to strand β1′ of the cap domain and shows a very similar conformation in chains A and C, while in chain B the loop bends in the opposite direction. Finally, res. 203–212 representing the β3′-β4′ loop (at the tip of the cap domain and close to the dimerisation interface) are found in two distinct shapes for the non-crystallographic (A–B) dimer on the one hand and the crystallographic (C–C) dimer on the other. The latter is affected by steric interference from a neighbouring chain. It is interesting to note that two of the segments displaying significant inter-chain variability (the αA′-αA and αC′-β1′ linkers, see above) enclose what has been identified as a potential product egress channel in PsmD, thus rationalising enhanced mobility in this region. The second gate proposed to control access to the catalytic cavity is formed by the N-terminal segment that we have dubbed the lid region. While its overall structure is reminiscent of the closed form observed for PsmD, the terminal portions deviate significantly between both enzymes in both sequence and conformation. Still, the Ω-loop formed by residues 2–14 of PsmD can be considered functionally analogous to the short, extended segment 7–9 of SgMT; while the side chain of M2, together with a bent conformer of R86, shields the SAH adenine moiety from solvent in PsmD, this function is accomplished in SgMT by the arginine (R81) assuming a straight conformation (Fig. S3†). These considerations imply that residues preceding T7 in SgMT, which could not be traced in the electron density and are thus considered disordered, may be dispensable for the actual lid functionality, possibly explaining why in SgMT, in contrast to PsmD, the presence of a His-tag at the N-terminus does not abolish enzymatic activity.

Comparing the catalytic cavities of the two methyltransferases reveals different levels of conservation. As expected, residues directly contacting the cofactor, at the bottom of the cavity, are mostly identical. Minor exceptions are A120 replacing PsmD residue T123, hence omitting one of the hydrogen bonds coordinating the SAH carboxyl group, G123 representing a conservative replacement of PsmD residue A126 owing to an analogous van der Waals (vdW) interaction with the ribose moiety, and L127 taking the position of Q130 as a vdW contact of the adenine amino group. Similarly, the three tyrosine residues covering the cofactor, Y11 (Y16 in PsmD), Y18 (Y23), Y126 (Y129), are conserved between the two enzymes and occupy very similar positions. Since this is also true for H220 (H218) and D218 (E216), these data suggest that SgMT features an equivalent catalytic triad (and hence most likely a similar mechanistic paradigm) as proposed for PsmD (Fig. S7†). We tested this hypothesis by exchanging the respective residues and monitoring the effects on enzymatic activity (Fig. 5C). Indeed, the replacements Y126A or Y126F completely abolished activity, indicating that the phenolic function is necessary. Similarly, H220 did not tolerate exchange by A, which is consistent with its proposed function as a proton acceptor for the nearby tyrosine (Fig. S7†). In position 218, conservative exchange D218E and even removing the functional group by mutagenesis to D218A preserved some residual activity, suggesting that (i) D218 exhibits minimal functionality even without further proton transfer or electrostatic stabilisation and (ii) full activity requires the acidic group to be presented in an orientation that the longer E side chain cannot accomplish. Overall, these results are consistent with the notion that a proton relay system akin to PsmD,^15^ favouring nucleophilic activation of the substrate by the catalytic tyrosine, is also functional in methyltransferases StspM1 and SgMT. Yet, since the actual substrate (cWW)—despite its inclusion in the crystallisation sample—was not detectable in the electron density, its interaction with the protein was investigated *in silico* (see below).

In the SgMT crystal structure, the D218 side chain forms a hydrogen bond with N13 in the lid region, *i.e.*, it points away from H220, while in PsmD, which lacks the competing hydrogen bonding partner in the lid, E216 and H218 were consistently found in contact as long as the cofactor was present. These observations may hint at a role of the lid segment in the regulation of catalytic activity, possibly by integrating information on substrate and cofactor availability. Alternatively, the proton-relay system operating in SgMT may differ from the one found in PsmD in mechanistic details (discussed below). In accordance with the disparate substrate specificities of PsmD on the one hand and SgMT/StspM1 on the other hand, significant differences can be observed in side chains lining the catalytic pocket. These exchanges imply notable alterations in both side chain size and polarity (exemplified by Q29 and F154 in SgMT *vs.* W34 and E158 in PsmD) at the respective positions.

### Binding mode of the substrate cWW 4a

**Fig. 6:**
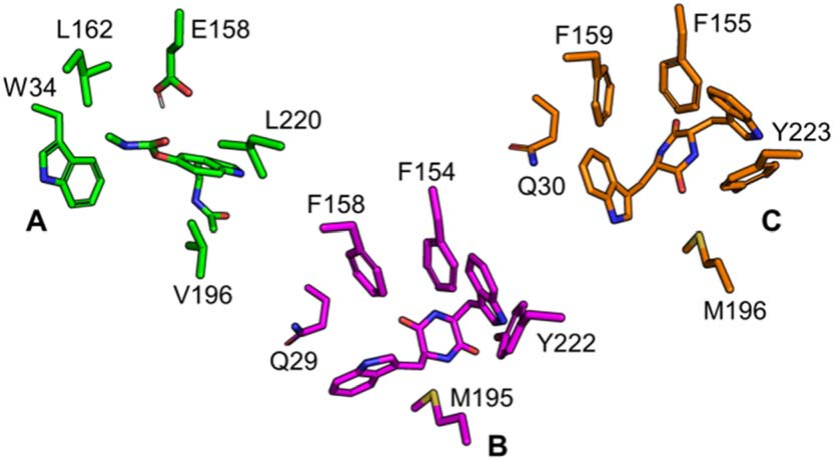
Superimposed binding sites and putative *ll*-cWW catalytic conformations extracted from MD simulations for PsmD (A), based on our previous work, SgMT (B), and StspM1 (C). The main differences in residues involved in substrate stabilization are shown at equivalent positions in the 3D structures.

To determine the *ll*-cWW substrate binding mode in relation to the identified catalytic residues, docking (Glide, Schrödinger 2024) was performed. MD simulations (8 replicas of 200 ns each) of the SgMT dimer bound to both the docked substrate and SAM cofactor were conducted to sample the substrate conformational dynamics and monitor its interaction with the surrounding residues. In accordance with our previous study,^11^ we used five geometric criteria (Fig. S3†) to extract substrate conformations that adopt a binding mode compatible with catalysis. The putative catalytic binding mode of *ll*-cWW in SgMT shows similarity with the one predicted for the *dd*-cWW enantiomer^11^ in StspM1. However, significant differences with the *ll*-cWW binding pose predicted in our previous work on StspM1 ^11^ can be observed, despite the high similarity between the StspM1 and SgMT substrate binding sites. Improving our previously published StspM1 3D model (see ESI†) corrected this discrepancy. New MD simulations (8 replicas, 200 ns each) performed in this work on this improved 3D model showed that the *ll*-cWW dominant binding pose in StspM1 over the simulation time is nearly identical to the one predicted in SgMT (Fig. 6).

**Fig. 7:**
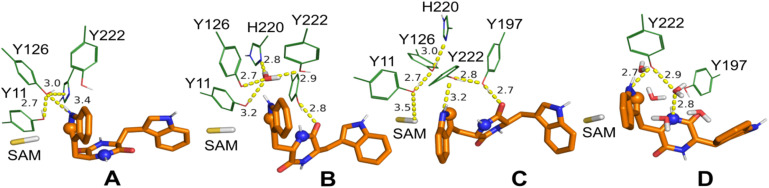
Putative *ll*-cWW catalytic conformations extracted from MD simulations. Grey sticks: reactive methyl group of SAM, orange sticks: *ll*-cWW, green lines: selected residues. Dashed lines: relevant hydrogen bond interactions with distances given in Å. As spheres: DKP nitrogen (blue sphere) and indole carbon (orange sphere) involved in pyrroloindole cyclization. (A) Conformation suitable for methyl transfer but not for pyrroloindole cyclization as the DKP nitrogen and indole carbon involved are too far from one another. (B)–(D) Conformations suitable for both methyl transfer and pyrroindole cyclization.

The comparative analysis of substrate binding sites (Fig. 6 and S8†) between SgMT, StspM1, and PsmD reveals that a few residue substitutions may be required to alter SgMT substrate scope and improve its activity for substrate homologues other than cWW. In PsmD, the presence of W34, L162, and E158 plays an important role for the binding of the substrate carbamate side chain. Per-residue MM-GBSA calculations for SgMT and StspM1 (Fig. S9†) suggest that Q29 in SgMT (Q30 in StspM1) is necessary to stabilize the indole side chain *via* hydrogen bonding of the sidechain amide group with the indole nitrogen. Superimposition of the PsmD and SgMT binding sites also shows that the presence of a tryptophan at this position (W33 in PsmD) would create a steric clash with bulky cWW substrates. Conversely, accommodating cWX substrate homologues (X being a smaller amino acid than tryptophan) might require substituting Q29 with either F or W to improve their packing in the binding site. In addition, we speculate that F158 may also be replaced by a more polar residue (like Y) to accommodate the histidyl side chain in cWH *via* the formation of additional hydrogen bonds. Due to the high similarity between SgMT and StspM1, these suggestions should also be transferable to StspM1. While most substrate binding poses adopted a geometry suitable for methyl transfer in SgMT (Fig. 7A), poses fulfilling all five geometry criteria and thereby presenting a geometry compatible with both steps of the catalytic mechanism (methyl transfer and pyrroloindole cyclization (Fig. 7B–D)) were found in only 13 snapshots out of the entire concatenated MD trajectory (containing 103 197 snapshots in total).

**Fig. 8:**
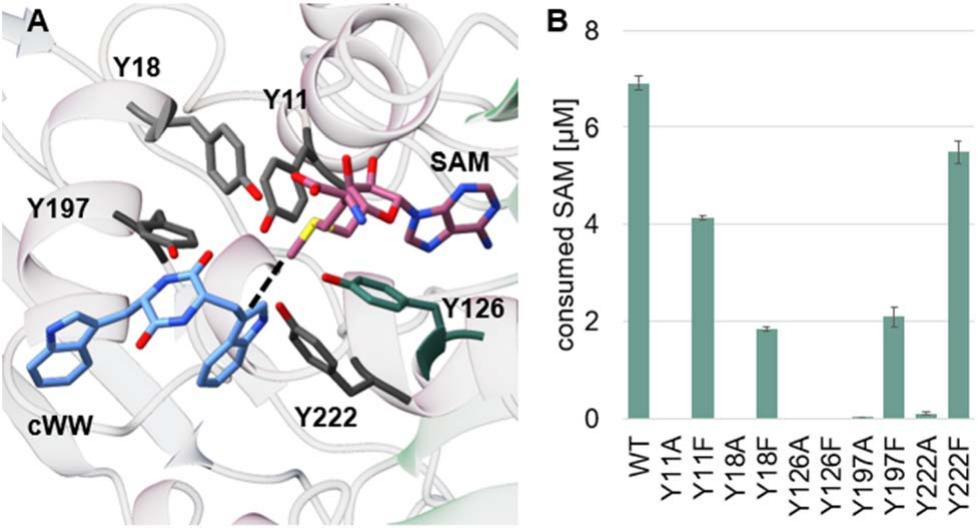
Tyrosine network in SgMT. (A) Tyrosines Y11, Y18, Y126, Y197, and Y222 are shown in grey, the cofactor in dark salmon and the docked substrate 4a in blue. The dashed line connects the SAM methyl group to the reactive indole moiety. (B) Results of the mutagenesis study probing the tyrosine network of SgMT. Enzyme activities were measured with the MTase Glo assay (Promega).

The simulations showed that both hydroxyl groups of Y126 and Y222 form polar interactions with the substrate reactive indole nitrogen (Fig. 8). A hydrogen bond formed between this group and the hydroxyl moiety of Y126 is found in six MD snapshots. As suggested in our previous works, this interaction may be important to increase the nucleophilicity of the reactive indole ring prior to methyl transfer.^15^ The phenolate form of Y126, stabilized by a hydrogen bond with H220, likely stabilizes the transient positive charge of the indolinium intermediate formed after methyl transfer, as already suggested.^11^

As opposed to our initial hypothesis regarding the involvement of D218 in a catalytic triad with Y126 and H220, our simulations did not reveal a direct interaction between H220 and D218. In contrast, we observed the formation of a hydrogen bond involving the side chains of H220 and Y126 in ten simulation snapshots, suggesting an alternative proton-relay system which may play a role in the electronic activation of the substrate (Fig. 7). Indeed, hydrogen-bonding of H220 to Y126, instead of D218, is perfectly consistent with our SgMT crystal structure (which does not contain a substrate moiety). It is also interesting to note that a water molecule hydrogen-bonded to the side chains of Y126, H220, and Y222 was observed in five simulation snapshots (Fig. 7B). This water molecule might help maintain the interaction between these residues when their side chains are further away from one another. While these data suggest that the proton relay system in SgMT may not depend on direct hydrogen bonding between D218 and H220, the aspartic acid is clearly required for full catalytic activity, most likely by creating a favourable electrostatic environment stabilising both the position and the protonation state of the histidine side chain. Y126 appears to engage in additional, direct as well as indirect, stabilising interactions, as outlined above.

In our previous study, the Y222 equivalent in StspM1 (Y223) was suggested to be potentially involved in catalysis by forming a hydrogen bond with the reactive indole nitrogen of the substrate. The inactivity of the Y223F mutant proved the importance of the hydroxyl group of Y223 in StspM1 catalysis. The equivalent Y222F mutation in SgMT only slightly decreased the activity in comparison to the WT enzyme (Fig. S4†). In contrast to StspM1, SgMT shows a network of five tyrosines pointing to the reaction center. To check their importance in catalysis, these residues were mutated into alanine and phenylalanine. All alanine mutants lost activity, whereas the phenylalanine mutants showed a decreased activity (except for Y126F which inactivates SgMT) (Fig. 8).

While this result indicates that the hydroxyl group of Y222 in SgMT is not essential for catalysis, the inactivity of the Y222A mutant confirms the catalytic importance of an aromatic ring at this position. However, conservation studies on 60 SgMT homologues showed that a glutamine residue is at this position in 67% of all aligned sequences (Fig. S5†). This result suggests that the presence of an aromatic ring at this position is a feature shared by a restricted group of methyltransferases, which includes SgMT and StspM1. In PsmD, a leucine is present at this position and may play a similar role in pre-orienting the side chain of the equivalent catalytic tyrosine (Y128) for catalysis. As observed for StspM1, the Y222 sidechain forms significantly more hydrogen bonds with the reactive indole nitrogen of the substrate than Y126 in our simulations (Fig. S6†). However, the relatively high activity of the Y222F mutant also indicates that these hydrogen bonds should not play a crucial role in catalysis. Conversely, the inactivity of the Y222A mutant and the proximity of this residue with Y126 suggests that the aromatic ring of Y222 more likely plays an essential role in correctly pre-orienting the Y126 side chain for catalysis. To assess whether the Y222F mutation could impact Y126 protonation state dynamics, constant pH MD simulations (8 replicas) at physiological pH were performed over a combined simulation time of 2 μs. Analysis of the titration statistics of Y126 in both WT and the Y222F mutant confirmed this hypothesis, revealing that the Y222F mutant reduces the deprotonation frequency of the Y126 hydroxyl group by a factor of two. This result suggests that, despite having a minor direct role in catalysis, the Y222 hydroxyl moiety may still influence the SgMT catalytic mechanism. QM/MM simulations will need to be performed to reveal further insights on this question. In addition, our classical MD simulations revealed that several water molecules interact with Y222 and the substrate DKP ring (Fig. 7D). A theoretical study^24^ showed that a proton relay mediated by hydrogen-bonded water molecules can assist the intra-molecular cyclization of C3-substituted tryptophan analogues into pyrroloindoline products. Following a similar mechanism, the polar character of the hydroxyl moiety of Y222 might help recruiting water molecules in the vicinity of the DKP ring. However, the relatively high activity of the Y222F mutant and the relatively low accessibility of water molecules in the active site when the substrate is bound suggest that this mechanism might as well occur without the help of Y222, albeit in a possibly less efficient way.

## Conclusions

In this study, the methyltransferase SgMT was extensively investigated. Following its biochemical characterization, the substrate scope was explored by testing 23 different DKPs and related substrates. To better understand its active site, the enzyme was crystallised and the structure was analysed in detail. An alanine scan confirmed the presence of a catalytic triad and a critical tyrosine network. Additionally, computational simulations were used to examine the positioning of the substrate within the active site and its interactions with catalytic residues. This in-depth analysis, which includes the first crystal structure (PDB ID: 9GDJ) of a DKP indole C3-methyltransferase, provides important insights for harnessing these enzymes as tools in (natural product) synthesis. SgMT is particularly suitable for this purpose, as it is expressed at levels around 25 times higher than the previously investigated StspM1.

## Data availability

The data supporting this article have been included as part of the ESI.† Coordinates and structure factor amplitudes for SgMT have been deposited in the wwPDB (https://www.wwpdb.org) with accession code 9GDJ (https://doi.org/10.2210/pdb9gdj/pdb).

## Author contributions

MH – conceptuation, data curation, formal analysis, investigation, methodology, validation, visualisation, writing – original draft. OHW – formal analysis, methodology, validation, supervision, resources. BD, EP, BP – formal analysis, methodology. HG – project administration, supervision, resources. JP – conceptuation, funding acquisition, project administration, supervision, resources. All authors contributed to writing – review & editing.

## Conflicts of interest

There are no conflicts to declare.

## Supplementary Material

SC-OLF-D4SC07300B-s001
