# Supplementary material for: Characterization of a C-methyltransferase from Streptomyces griseoviridis – crystal structure, mechanism, and substrate scope
Source: Chem Sci. 2025 Jan 30;16(10):4519–27. doi: 10.1039/d4sc07300b (PMC11804793; doi:10.1039/d4sc07300b)
Supplement: SC-016-D4SC07300B-s001 [file SC-016-D4SC07300B-s001.pdf]

Supplementary Information (SI) for Chemical Science.
This journal is © The Royal Society of Chemistry 2025

Characterization of a *C*-methyltransferase from *Streptomyces griseoviridis* – crystal structure, mechanism, and substrate scope

Mona Haase[a], *Oliver H. Weiergräber**[b], *Benoit David*[c], *Elias Pfirmann*[a], *Beatrix Paschold*[a], *Holger Gohlke**[c,e], *Jörg Pietruszka**[a,d]

[a] Institute for Bioorganic Chemistry & Bioeconomy Science Center (BioSC), Heinrich Heine University Düsseldorf in Forschungszentrum Jülich, 52426 Jülich, Germany

E-mail: j.pietruszka@fz-juelich.de

[b] Institute of Biological Information Processing (IBI-7: Structural Biochemistry) Forschungszentrum Jülich, 52425 Jülich, Germany

E-mail: o.h.weiergraeber@fz-juelich.de

[c] Institute of Bio- and Geosciences (IBG-4: Bioinformatics) Forschungszentrum Jülich, 52426 Jülich, Germany

[d] Institute of Bio- and Geosciences (IBG-1: Bioorganic Chemistry) & Bioeconomy Science Center (BioSC), Forschungszentrum Jülich, 52426 Jülich, Germany

[e] Institute for Pharmaceutical and Medicinal Chemistry & Bioeconomy Science Center (BioSC), Heinrich Heine University Düsseldorf, 40225 Düsseldorf, Germany

E-mail: gohlke@uni-duesseldorf.de

Table of contents

Biological methods S4

Protein sequence and vectors S4

Bacterial strains and media S4

Transformation S5

Protein expression S5

Enzyme purification (large scale) S6

Enzyme purification (small scale) S6

General procedure for Mtase glo assay S7

In-vitro assay S7

HPLC method S7

His-Tag removal S8

Enzymatic preparative scale reaction with immobilized enzyme S8

Computational methods S9

Structure prediction S9

Docking calculations S9

Classical molecular dynamics simulations S9

Constant-pH molecular dynamics simulations S11

Geometric analyses S11

Binding sites comparison in PsmD, StspM1, and SgMT S12

Per-residue binding free energy decomposition S12

Residue conservation analysis S13

X-ray crystallography S13

Chemical methods S14

General information S14

General procedure of diketopiperazine synthesis[1] S15

Synthesis of benzodiazepinedione[44] S16

Analytical data S17

TRP-TRP-DKP S18

TRP-PHE-DKP 20

TRP-TYR-DKP S24

TRP-HIS-DKP S27

TRP-ALA-DKP S30

TRP-VAL-DKP S31

TRP-LEU-DKP S32

Benzodiazepinedione S33
Single C3-methylated *LL*-cWW 13a S35
Single C3-methylated *DD*-cWW 13b S36
Double C3-methylated LL-cWW 5 .... S37
Figures and tables S39
Literature S67

Biological methods

Protein sequence and vectors

The gene sequence for the methyltransferase SgMT as well as the variants of the alanine scan were codon harmonized and synthetically constructed in a pET28a vector (GenScript, USA).

GenBank: GGT26788.1:

N-terminally His-tagged SgMT:

MGSSHHHHHHSSGLVPRGSHMSSQTVTPDPYGNLAESYDRLAQWAIDQQQESPRDRVGDFLQTFWQS
QDRPVRTVLEICCGTGLMLAELARRGYVVTGLDRSAAMLEQARARMGGKTTLIRAELPDIPAPAGEFD
AVVSAAGGLNYLSESQISATFGAVARLLPAGGTFTFDVFGQGFYAKFFDPSAPRVMALELDDISYIWTF
TKPAEAPFVDMSYTQFSPASRAVDGEPAFIRTRDLHRYYPLPHATVLRLAAEHGFTDARAHDNYSSDPS
GPHTLYDTWTMVRTGSLE*

C-terminally His-tagged SgMT:

MGHMSSQTVTPDPYGNLAESYDRLAQWAIDQQQESPRDRVGDFLQTFWQSQDRPVRTVLEICCGTGL
MLAELARRGYVVTGLDRSAAMLEQARARMGGKTTLIRAELPDIPAPAGEFDAVVSAAGGLNYLSESQI
SATFGAVARLLPAGGTFTFDVFGQGFYAKFFDPSAPRVMALELDDISYIWTFTKPAEAPFVDMSYTQFS
PASRAVDGEPAFIRTRDLHRYYPLPHATVLRLAAEHGFTDARAHDNYSSDPSGPHTLYDTWTMVRTGS
LEHHHHHH*

Amino acid sequence for SgCDPS (consensus motif 1 is marked in green, consensus motif 2 is marked in grey)

GenBank: GGT26792.1

MRYVPNGLGPGTYCSVPAHMEFSIEPMTENCYSVCQRREHVVLGVSPGNSFFKVPLLTDLIRWLSREFA
RLDIVVPDVELSTTFTSLGYPPGRAARKALAEVNAVRNRVVRAWQALGGPRPCDGLHLMSDLVDRSR
YRTARAACEKALREDETLRVTCREASRVVLRARRPGSEPTAEAVEQAMRYLLAELPFFIASADIFDVPSS
LCFYHRPLPLAELVFSGRTVLKPGPQQGYALVRPVAPPAGPAAQRAVPDT

Bacterial strains and media

E. coli BL21(DE3) was selected for cultivation and protein expression. For pre cultures, LB (lysogeny broth) liquid medium (10 g·L^{-1} tryptone, 5 g·L^{-1} yeast extract, 2 g·L^{-1} sodium chloride) and for main cultures TB (terrific broth) liquid medium (Carl Roth, Karlsruhe, Germany: 12 g·L^{-1} casein, 24 g·L^{-1} yeast extract, 12.54 g·L^{-1} K_2HPO_4, 2.3 g·L^{-1} KH_2PO_4, 4 mL·L^{-1} glycerol) with kanamycin (final concentration of 100 µg·mL^{-1}) was used. The culture media were prepared with distilled water and sterilized by autoclaving.

Transformation

Competent *E. coli* BL21(DE3) cells were transformed by heat shock with the desired plasmid. For this, 100 ng plasmid DNA was added to 100 µL of competent cells and incubated for 30 min on ice. After incubation, the cells were heated in a water bath at 42 °C for 90 seconds. 700 µL LB medium was added and the cells were inverted with an overhead shaker at 37 °C for 1 hour. The transformed cells were centrifuged at 2000 rpm for two minutes to harvest a cell pellet. The cell pellet was resuspended in 100 µl LB medium and cultured on LB medium agar plates with kanamycin at 37 °C overnight.

Protein expression

As a preculture, 5 mL of LB medium with kanamycin was inoculated with a colony from the transformed cells and incubated at 37 °C for 16 hours. The preculture was used to inoculate the main culture (500 mL TB medium) at a dilution of 1:100. The main culture was incubated at 37 °C and 130 rpm until an OD600nm of 0.5 was reached. The protein expression was induced by adding the inducer IPTG (final concentration of 100 µM). After incubation at 25 °C for 20 h, the cells were harvested by centrifugation at 7000 g for 35 minutes at 4 °C. The cell pellet was stored at -20 °C until further use.

Enzyme purification (large scale)

The cells were resuspended at a concentration of 0.2 g/mL in KPi buffer (50 KPi, pH 8) and lysed by sonication with an ultrasonic cell disruptor (Branson Sonifier II "Modell W-250", Heinemann) two times for 10 min (amplitude of 35–40%). After centrifugation at 7000 rpm for 30 min, the lysate was loaded on a Ni-NTA affinity chromatography column (5 mL HiTrap HP column, Bio-Rad) with a flow rate of 3 mL/min. A washing step was performed by washing the column with 15 mL 50 mM KPi (40 mM ImH, pH 7.5). The SgMT was eluted with 15 mL of the elution buffer (50 mM KPi, 250 mM ImH, pH 7.5). To exchange the buffer and concentrate the protein, protein concentrators (Vivaspin 20, 10000 MWCO PES, Sartorius Stedim Biotech) were used. The concentrated protein solution in KPi buffer (50 mM, pH 8) was frozen in liquid nitrogen and kept at −20 °C until usage.

Enzyme purification (small scale)

200 mg cells were resuspended in 1 mL KPi buffer (50 KPi, pH 8) and lysed by glass bead (0.2 mm) cell disruption for 10 min and 30 Hz with a swing mill (Retsch, Type MM400). After centrifugation at 7000 rpm for 30 min, the lysate was added to 100 µL washed Ni-NTA agarose beads and incubated for 40 min by slowly inverting on ice. A washing step was performed by washing the beads with 1 mL 50 mM KPi (40 mM ImH, pH 7.5). The SgMT was eluted with 500 µL of the elution buffer (50 mM KPi, 250 mM ImH, pH 7.5). To exchange the buffer and concentrate the protein, centrifugal concentrators (Vivaspin 500, 10000 MWCO PES, Sartorius Stedim Biotech) were used. The concentrated protein solution in KPi buffer (50 mM, pH 8) was frozen in liquid nitrogen and kept at −20 °C until usage.

General procedure for Mtase glo assay

The commercially available Mtase Glo assay (Promega) was used to determine the activity of the methyltransferase according to the manufacturer's protocol. The luminescence was measured using a plate reader (Infinite plate reader, Tecan). Every reaction was carried out as triplicate in a 96-well plate (Nunc™ 96-well plates (Nunclon®; Polystyrene, flat bottom, white). The reactions were performed with 3 µM enzyme and a final concentration of 25 µM SAM and the additional substrate at 45 °C and terminated after five, ten, or 15 min and ended by adding 0.5 % TFA. The kinetic parameters were determined using the Michaelis-Menten equation and calculated with the software Origin Pro 2017G (OriginLab Corporation, Northampton, MA, USA).

***In-vitro* assay**

The substrates were dissolved in DMSO (100 mM). The reactions were performed with 34 Vol% lysate in KPi buffer (pH 8, 50 mM) with an end concentration of 1 mM substrate and 3 mM SAM at 45 °C and 700 rpm. The reactions were stopped by adding 1 Vol% TFA.

HPLC method

To compare the retention times of the products with the references, the following HPLC-method was used:[1]

RP-HPLC: Jasco HPLC (pump: PU-4180, thermostat: CO-2060Plus, autosampler: AS-4050)
Stationary Phase: hyperclone column (5µ ODS C18, 125*4 mm, 120 Å, Fa. Phenomenex)
Mobile phase A: Acetonitril + 0.1% formic acid (v/v) and B: water + 0.1% formic acid (v/v)
Gradient: 0-2 min: 10:90 (A:B), 2-15 min: 10% A to 50% A, 15-25 min: 50% A to 95% A,

25-30 min: 95:5 (A:B), 30-35 min: 10:90 (A:B).

Flow rate: 0.8 mL/min

Column temperature: 25 °C

Detection wavelength: 284 nm

His-Tag removal

Thrombin from bovine plasma ordered from Sigma-Aldrich was used to cleave the His tag. The reaction was carried out in buffer (KPi buffer 10 mM with 140 mM NaCl, 2.7 mM KCl, pH 7) with 50 U thrombin per mg enzyme overnight at 16 °C and 700 rpm.

Enzymatic preparative scale reaction with immobilized enzyme

A total of 18.4 g of cell pellet was lysed in 92 mL of reaction buffer (50 mM KPi, pH 8). The lysate was prepared by sonicating the cells in lysis buffer using an ultrasonic cell disruptor (Branson Sonifier II "Model W-250", Heinemann) for 10 minutes at 35–40% amplitude, performed twice. After centrifugation (4°C, 10,000 × rcf, 20 minutes), the supernatant was collected as the lysate. The lysate was then incubated with 9 mL of washed Protino® Ni-NTA agarose suspension at 4°C for 40 minutes on an end-over-end shaker. After removing the supernatant, 60 mL of washing solution (50 mM KPi, pH 8, 80 mM ImH) was added and incubated for 10 minutes at 4°C on an end-over-end shaker. The supernatant was discarded, and the immobilized enzyme was washed twice with reaction buffer (50 mM KPi, pH 8) to remove any residual imidazole. The enzyme-bound Ni-NTA material was transferred into a glass bottle and the volume was adjusted to 270 mL with buffer. The cWW substrate **4a** (100 mg, 0.27 mmol) was dissolved in DMSO (1.34 mL) and added to the reaction mixture, along with S-Adenosylmethionine disulfate tosylate (618 mg, 0.81 mmol). The reaction was

carried out in KPi buffer (50 mM, pH 8) at a final concentration of 1 mM cWW substrate, at 40°C and 300 rpm. After 48 hours, the reaction was stopped by removing the Ni-NTA material via filtration. The product was extracted with ethyl acetate (three times), the organic phase was dried with $MgSO_4$, and concentrated under reduced pressure. The final products were purified via column chromatography (using ethyl acetate), yielding 96 mg (0.24 mmol, 89%) of the product.

Computational methods

Structure prediction

Based on the crystal structure of the monomer, the dimeric structure of SgMT was predicted using AlphaFold-multimer[2, 3] in ColabFold[4]. The SAM cofactor was docked in the binding pocket using the coordinates of the SAM cofactor modeled in StspM1[1].

Docking calculations

The binding poses of *LL*-cWW was predicted using Glide SP[5,6] (Schrödinger 2024-1) with the OPLS_2005 force field[7]. The protein protonation state was set using the implementation of PROPKA 3.0[8] in the Protein Preparation Wizard module from Schrödinger[9]. Conformational sampling of *LL*-cWW was performed using the LigPrep module at pH 7.4 with Epik[10]. By analogy with our previous work[1], a harmonic distance restraint of 10 $kcal\,mol^{-1}\,Å^{-2}$ was enforced to ensure that the substrate carbon atom involved in the methyl transfer stays within a radius of 3 Å to the SAM methyl group. The substrate was docked in the presence of the SAM cofactor in both subunits of the dimeric structure.

Classical molecular dynamics simulations

The SgMT dimer model in complex with both SAM and the docked *LL*-cWW substrate was subjected to eight replicas of classical MD simulations of 200 ns length each. The protonation state of all titratable residues was set using PROPKA 3.0[8]. Despite its high predicted pKa, the

buried Glu57 was kept deprotonated due to its interaction with the positive charge of the protonated SAM tertiary amine group. Similarly, Glu31 was left deprotonated, despite its relatively basic character (pKa ~ 6.5). Constant pH MD simulations (6 replicas) performed on SgMT in explicit TIP3P solvent at pH 7 showed that, while the protonated form of Glu31 was predicted in two out of six simulation replicas, the deprotonated form is the dominant state at physiological pH. RESP charges[11] for the ligands were derived using Multiwfn[12] (version 3.812) from the electrostatic potentials calculated at the HF/6-31G* level of theory using the ORCA 5.0.3 simulation software[13]. For compatibility with traditional AMBER force fields, RESP fitting was performed using a two-stage fit, considering the equivalent symmetries of hydrogens of methyl groups in the second stage. The ligands non-bonded force field parameters were derived from the gaff2 force field[14] using the parmchk2 module of the Antechamber program[15] from AmberTools 23[16]. The protein was modeled using the ff14SB force field[17] and simulated in a truncated octahedral box solvated with TIP3P water[18] molecules. The net charge of the system was neutralized by the addition of sodium ions. The Particle Mesh Ewald algorithm[19] was used to model long-range electrostatic interactions beyond an inter-atomic distance of 9 Å. Minimization was performed using the pmemd.MPI module of the AMBER 23.1 software[20]. Equilibration and MD production were performed using pmemd.cuda[21]. The system was minimized in three stages, including 1,000 steps of steepest-descent applied to the solvent molecules only, followed by 10,000 steps of steepest descent and 40,000 steps of conjugate gradient minimization of the whole unrestrained system. Harmonic distance restraints of 10 kcal mol^{-1} $Å^{-2}$ were applied to keep the substrate's reactive carbon atom close to the SAM methyl group to model a substrate near-attack configuration. Thermalization of the system at 300 K was performed for 500 ps using Langevin dynamics in the NVT ensemble. Density equilibration was performed in the NPT ensemble at 1 bar for 10 ns. For both equilibration steps, positional restraints of

10 kcal mol^{-1} Å^{-2} were applied on the solute. A 2 fs timestep was used together with the SHAKE algorithm[22]. Simulation snapshots were recorded every 10 ps.

Constant-pH molecular dynamics simulations

Simulations were perfomed in explicit TIP3P solvent at pH 7 using AMBER 23.1. Other simulation settings are the same as for the classical MD simulations described above. Titration statistics were analyzed using the cphstats program implemented in AmberTools 23[16].

Geometric analyses

The CPPTRAJ program[23] (AmberTools 23[16]) was used for all geometric (distances, angles) analyses. The geometric criteria used to determine hydrogen bonding were a donor (D) – acceptor (A) maximum distance of 3.5 Å and a D-H...A angle greater than 120°. In agreement with our previous study[1], five geometry criteria were used to isolate putative catalytic (reactive) *LL*-cWW conformations from MD trajectories. First, we considered all binding poses having a distance between the cofactor reactive methyl moiety ($C_{\text{SAM-methyl}}$) and the nucleophilic carbon ($C7_{\text{cWW-indole}}$) of the reactive indole ring of *LL*-cWW of less than 3.2 Å. Second, we suggest that the angle formed by the SAM methyl donor and acceptor atoms (S_{SAM} – $C_{\text{SAM-methyl}}$ – $C7_{\text{cWW-indole}}$) should be between 160 (ideally 170°) and 180°. Third, we suppose that an orthogonal orientation of the reactive indole ring to the cofactor S_{SAM} – $C_{\text{SAM-methyl}}$ axis, which can be monitored by the $C_{\text{SAM-methyl}}$ - $C7_{\text{cWW-indole}}$ - $C1_{\text{cWW-indole}}$ angle, should be necessary to allow an optimal exposure of the indole ring to the sulfonium ion. Fourth, we suggest that both $C8_{\text{cWW-indole}}$ and $N2_{\text{cWW-DKP}}$ atoms involved in the pyrroloindole ring formation in *LL*-cWW should be on the same side of the DKP ring, within a maximal distance of ~3.5 Å from each other. Finally, based on distance measurements in the crystal structures of other structurally homologous methyltransferases, we assume that the hydroxyl

moiety of the Y126 in SgMT should be within 6 Å away from the reaction center (SAM sulfur atom).

Binding sites comparison in PsmD, StspM1, and SgMT

Representative substrate binding poses for PsmD, StspM1, and SgMT were selected from single snapshots extracted from MD simulation trajectories (combined replicas) performed either in the previous (for PsmD: https://pubs.acs.org/doi/10.1021/acscatal.2c04240) or current study (for StspM1 and SgMT). 2D sketches of the substrate binding sites were drawn using LigPlus (https://pubs.acs.org/doi/10.1021/ci200227u). The StspM1 MD simulations conducted in this study were based on an improved StspM1 3D model. In contrast to our previous work[1], the modeling of the StspM1 dimeric interface was improved using the implementation of AlphaFold-multimer-v3[2, 3] in ColabFold[4]. In addition, residue Glu32 (Glu31 in SgMT) was kept deprotonated based on constant pH MD simulation results (see Classical molecular dynamics simulations section).

Per-residue binding free energy decomposition

MM-GBSA calculations of the effective binding free energy (ΔG_{eff}) per residue were performed with the GB^{OBC} model (https://doi.org/10.1021/jp994072s) using MMPBSA.py (https://doi.org/10.1021/ct300418h). Based on published (https://doi.org/10.1073/pnas.1506792112; https://doi/full/10.1002/jcc.20793) mechanistic studies on other methyltransferases, MM-GBSA calculations were performed on ensembles of conformations identified as reactant states. Reactant states are substrate conformers showing a maximum distance of 3.5 Å between the reactive substrate carbon (C7 atom) and SAM methyl carbon (C3 atom) as well as having a C7-C3-S1 angle value between 160 and 180°. An implicit ionic concentration of 0.1 M was used. ΔG_{eff} values were averaged over eight independent simulation replicas of 200 ns each after the exclusion of the first 10 ns. Snapshots were considered every 40 ps to minimize auto-correlation across samples in the calculation of the average ΔG_{eff}.

Residue conservation analysis

Sequence homologues of SgMT were retrieved from the UniRef90 database[24, 25] using HMMER[26] available from the ConSurf webserver[27-31]. Additional sequences of homologs were retrieved from the UniRef30 and environmental databases using the MMSEQS2 algorithm[32] implemented in ColabFold[33]. Redundant sequences were filtered out, and only sequences with 30% to 95% sequence identity between each other were kept. A multiple sequence alignment was performed using MUSCLE[34]. Residue conservation was calculated using WebLogo 3[35,36].

X-ray crystallography

The SgMT protein was crystallised using the vapour diffusion method in sitting-drop geometry, using robotic systems Freedom Evo (Tecan; Männedorf, Switzerland) and Mosquito LCP (SPT Labtech; Melbourn, United Kingdom) with commercially available screening sets. Protein crystals were observed for a number of conditions, several of which were subjected to matrix optimisation and additive screening. The diffraction-quality sample used for structure determination in this study was obtained with a reservoir solution containing 85.5 mM sodium citrate pH 5.6, 17.1% (v/v) isopropanol, 17.1% (w/v) PEG 4000, 4.5% (v/v) glycerol, 10 mM hexammine cobalt(III) chloride and a protein solution containing 8.4 mg/ml SgMT with 2.2 mM SAH and 2 mM LL-cWW. Prior to cryocooling, the crystal was shortly incubated in reservoir solution supplemented with 12% (v/v) glycerol. Diffraction data (doi.org/10.15151/ESRF-DC-1848199439) were recorded at 100 K on beamline ID23-1 of the European Synchrotron Radiation Facility (ESRF; Grenoble, France) tuned to an X-ray wavelength of 0.8856 Å, using an EIGER2 CdTe 16M detector (DECTRIS; Baden-Daettwil, Switzerland). Following data integration and scaling with XDS[37], the structure of SgMT in the

presence of SAH was determined by molecular replacement using MOLREP[38] with the crystal structure of the methyltransferase PsmD from *Streptomyces griseofuscus* (PDB-ID 7ZKH) as a search model. Following automated rebuilding in phenix.autobuild[39], the model was iteratively improved by reciprocal space refinement in phenix.refine[40] alternating with interactive rebuilding in COOT[41]. Assessment via MolProbity[42] and the wwPDB validation system confirmed good model geometry with no outliers in the Ramachandran plot and a proportion of rare side chain rotamers in the expected range. Full data collection and refinement statistics is provided in Table S2. Coordinates and structure factor amplitudes have been deposited in the wwPDB with PDB code 9GDJ (doi.org/10.2210/pdb9gdj/pdb).

Chemical methods

General information

For reactions under inert conditions, flame-dried glassware was used and an inert atmosphere (N_2 or Ar gas) was applied. Thin layer chromatography was performed with TLC-foil Polygram SilG/UV254 by Macherey-Nagel as a stationary phase and ethyl acetate as a mobile phase. A cerium-molybdate-solution [10 g $Ce(SO_4)2 \cdot 4\ H_2O$, 25 g phosphomolybdic acid, 60 mL conc. H_2SO_4, 940 mL H_2O] was used for staining of the TLC plates. Preparative column chromatography was performed using silica gel (0.040−0.063 mm) from Merck as the stationary phase and ethyl acetate as the mobile phase. ^1H, ^{13}C, and 2D NMR (COSY, HSQC, HMBC) spectra were measured with a Bruker Avance DRX 600 NMR. The optical rotation was measured using a JASCO P-2000 polarimeter. IR spectra were measured with a PerkinElmer SpectrumTwo spectrometer. High-resolution ESI mass spectra were measured using an MDS SCIEXQ Model Trap 4000 mass spectrometer (HHU Center of Molecular and Structural Analytics at the Heinrich-Heine-Universität Düsseldorf)

General procedure of diketopiperazine synthesis[1]

The benzyl chloroformate-protected *L*-tryptophan **S1** (1.0 equiv) and methyl-protected corresponding amino acid **S2 (**1.0 equiv) were dissolved in dry THF (0.25 mmol·mL^{-1}) at 0 °C. After Bis(2-oxo-3-oxazolidinyl)phosphinic chloride (BOPCl) (3.0 equiv) was added, the reaction was stirred for 3 h at 0 °C and allowed to warm up to 21 °C over night. 20 mL water were added and the product was extracted with ethyl acetate (3 x 20 mL). The organic phases were washed with brine and dried over $MgSO_4$. The solvent was removed under reduced pressure. The crude product **S3** was filtered over silica with ethyl acetate and used without further purification for the next reaction step.

To deprotect the amine, the crude products **S3** were dissolved in MeOH/ethyl acetate (1:2) (0.05 mmol·mL^{-1}) under nitrogen atmosphere. After adding ammonium formate (4.8 equiv) and 10% Pd/C (0.1 equiv.), the reaction mixture was stirred for 30 minutes under reflux conditions. Ther Pd/C residue was filtered off and the solvent was removed under reduced pressure. The crude product **S4** was used without further purification for the next step by dissolving it in 7 N methanolic ammonia (0.05 mmol·mL^{-1}). The reaction was stirred at 60 °C for 4 h. The solvent was removed under reduced pressure. The final product **S5** was purified using column chromatography with ethyl acetate as solvent.

For the tryptophan-histidine diketopiperazines, a different coupling reaction was chosen.[43] The methyl-protected histidine **S2** (1.0 equiv.) was dissolved in dry DMF (0.5 mmol·mL^{-1}) with the benzyl chloroformate-protected *L*-tryptophan **S1**. N-methyl morpholine (2.0 equiv) was added at 0 °C. EDC-HCl (1.0 equiv) was added portion-wise and the reaction was stirred at 0 °C for 3 h and additional 16 h at 21 °C. The residue was taken up in ethyl acetate and

washed with $NaHCO_3$ solution and water. The organic phases were dried over $MgSO_4$ before removing the solvent under reduced pressure. The crude product **S3** was filtered over silica with ethyl acetate and used without further purification for the next reaction step.

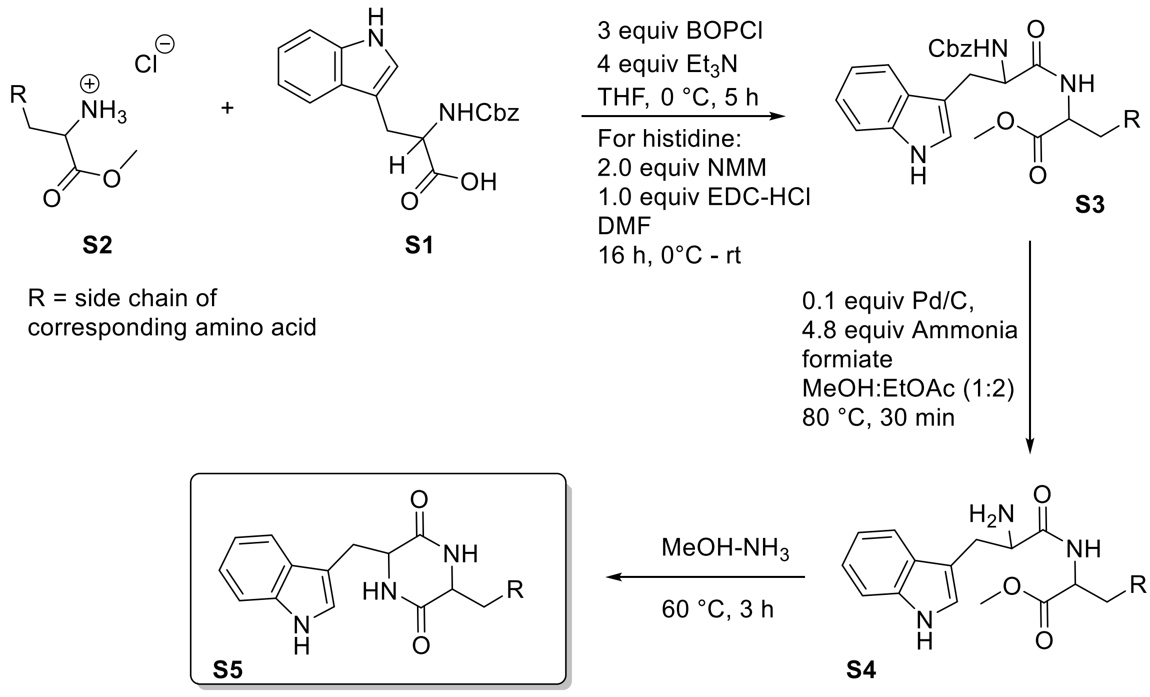

Scheme S1: Synthesis of the DKP substrates.

Synthesis of benzodiazepinedione[44]

Tryptophan **S6** (500 mg, 2.47 mmol, 1.0 equiv.) and isatoic anhydride **S7** (427.48 mg, 2.62 mmol, 1.06 equiv.) were solved in a 25 mL Schlenk flask in water. Triethylamine (0.345 mL, 2.47 mmol, 1.0 equiv.) was added and the reaction mixture was stirred at 21 °C for 5 h. The solvent was removed under reduced pressure before adding 6 mL of anhydrous acetic acid and heating the reaction to reflux for 5 h. The solvent was removed under reduced and the residue was taken up in ethyl acetate and washed with 100 mL sodium hydrogen carbonate. After extraction with ethyl acetate, the combined organic phase was washed with brine and

dried over $MgSO_4$. The solvent was removed under reduced pressure. The product **S8** was recrystallized with ethyl acetate and pentane giving a white solid.

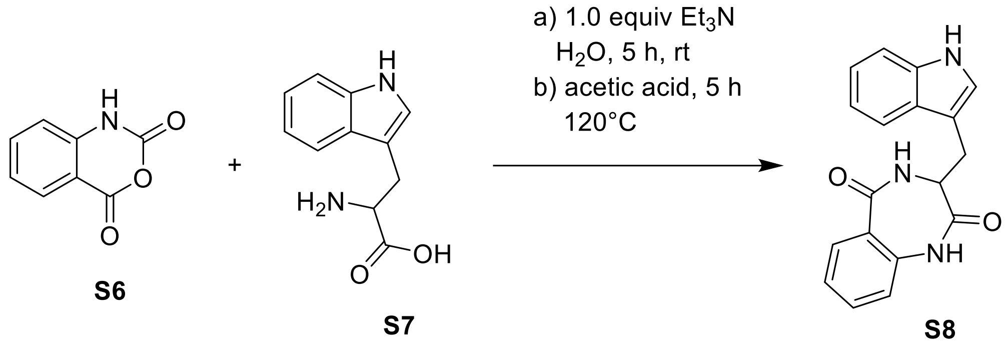

Scheme S2: Synthesis of the benzodiazepinedione substrates.

Analytical data

Table S1: Final yields over 3 steps of the substrate synthesis

	Nr.	Final yield [%]	Scale of final product [mmol]	Scale of final product [mg]
cWW	**4a**	91	1,11	409
	**4b**	90	1,38	514
	**4c**	85	0,80	298
cWY	**6a**	71	1,21	423
	**6b**	89	1,21	424
	**6c**	85	1,19	415
	**6d**	55	0,98	343
cWF	**7a**	91	1,35	449
	**7b**	90	1,34	447
	**7c**	75	1,20	401
	**7d**	74	0,98	328
cWH	**8a**	57	0,99	320
	**8b**	35	0,59	190
	**8c**	46	0,55	178
	**8d**	41	0,51	165
cWA	**9**	62	1,53	393
cWV	**10**	80	0,77	220
cWL	**11**	77	0,87	260
cWBenz	**12a**	31	0,76	231
	**12b**	33	0,78	249

TRP-TRP-DKP

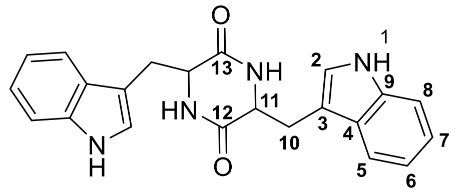

LL-cWW **4a**

^{1}H NMR (600 MHz, MeOD) δ 7.47 (d, $^3J_{8,7}$ = 8.0 Hz, 2H, H-8), 7.32 (d, $^3J_{5,6}$ = 8.1 Hz, 2H, H-5), 7.11 (dd, $^3J_{7,8}$ = 8.1, $^3J_{7,6}$ =6.9 Hz, 2H, H-7), 7.03 (dd, $^3J_{6,7}$ = 8.0, $^3J_{6,5}$ = 7.0 Hz, 2H, H-6), 6.49 (s, 2H, H-2), 4.06 (dd, $^3J_{11,10a}$ = 3.8 Hz, $^3J_{11,10b}$ = 7.2 Hz, 2H, H-11), 2.94 (dd, $^3J_{10a,10b}$ = 14.8, $^3J_{10a,11}$ = 3.8 Hz, 2H, Ha-10), 2.19 (dd, $^3J_{10b,10a}$ = 14.8, $^3J_{10b,11}$ = 7.3 Hz, 2H, Hb-10).

^{13}C NMR (151 MHz, MeOD) δ 169.71 (C-12), 138.05 (C-9), 128.61 (C-4), 125.90 (C-2), 122.52 (C-7), 120.07 (C-6), 119.68 (C-8), 112.41 (C-5), 109.45 (C-3), 56.86 (C-11), 31.39 (C-10).

IR: IR (ATR): ṽ [cm^{-1}] = 3266 (s), 3053 (vw), 2926 (w), 1704 (m), 1664 (vs), 1457 (s), 1360 (m), 1325 (m), 1230 (m), 1011 (w), 743 (s), 535 (vw).

MS: MS (ESI, 60 eV): m/z (%) = 373.17 (80) [M$^+$]

Optical rotation: $[\alpha]_D^{25}$ = *-77 (c* 0.4*, DMSO)*

Melting point: 254.3 °C

The analytical data are consistent with the literature.[1]

DD-cWW **4b**

^{1}H NMR (600 MHz, MeOD) δ 7.45 (d, $^3J_{8,7}$ = 8.0 Hz, 2H, H-8), 7.30 (d, $^3J_{5,6}$ = 8.1 Hz, 2H, H-5), 7.09 (dd, $^3J_{7,8}$ = 8.1, $^3J_{7,6}$ 7.0 Hz, 2H, H-7), 7.01 (dd, $^3J_{6,5}$ = 8.0, $^3J_{6,7}$ = 7.0 Hz, 2H, H-6), 6.46 (s, 2H, H-2), 4.05 (dd, $^3J_{11,10b}$ = 7.0, $^3J_{11,10a}$ = 3.8 Hz, 2H, H-11), 2.92 (dd, $^3J_{10a,11}$ = 14.4, $^3J_{10a,10b}$ = 3.8 Hz, 2H, Ha-10), 2.17 (dd, $^3J_{10b,11}$ = 14.4, $^3J_{10b,10a}$ = 7.2 Hz, 2H, Hb-10).

^{13}C NMR (151 MHz, MeOD) δ 169.73 (C-12), 138.09 (C-9), 128.64 (C-4), 125.90 (C-2), 122.54 (C-7), 120.09 (C-6), 119.69 (C-8), 112.42 (C-5), 109.50 (C-3), 56.91 (C-11), 31.40 (C-10).

IR: IR (ATR): ṽ [cm^{-1}] = 3266 (vs), 2923 (w), 1703 (m), 1663 (vs), 1457 (s), 1359 (s), 1325 (s), 1229 (m), 1091 (m), 1011 (w), 743 (vs), 524 (vw).

MS: MS (ESI, 60 eV): m/z (%) = 373.17 (80) [M$^+$]

Optical rotation: $[\alpha]_D^{25}$ = *66 (c 0.3, DMSO)*

Melting point: 249.0 °C

The analytical data are consistent with the literature.[1]

LD-cWW **4c**

^{1}H NMR (600 MHz, MeOD) δ 7.43 (d, $^3J_{8,7}$ = 8.0 Hz, 2H, H-8), 7.28 (d, $^3J_{5,6}$ = 8.2 Hz, 2H, H-5), 7.07 (dd, $^3J_{7,6}$ = 8.2 Hz, $^3J_{7,8}$ = 7.0 Hz, 2H, H-7), 6.99 (t, $^3J_{6,5}$ = 8.0 Hz, $^3J_{6,7}$ = 7.0 Hz, 2H, H-6), 6.44 (s, 2H, H-2), 4.03 (dd, $^3J_{11,10}$ = 7.1 Hz, 2H, H-11), 2.90 (dd, $^3J_{10a,11}$ = 14.4, 2H, Ha-10), 2.14 (dd, $^3J_{10b,11}$ = 14.4 Hz, 2H, Hb-10).

^{13}C NMR (151 MHz, MeOD) δ 169.72 (C-12), 138.08 (C-9), 128.63 (C-4), 125.90 (C-2), 122.53 (C-7), 120.09 (C-6), 119.69 (C-8), 112.42 (C-5), 109.50 (C-3), 56.90 (C-11), 31.40 (C-10).

IR: IR (ATR): ṽ [cm^{-1}] = 3304 (s), 3055 (vw), 2927 (w), 1670 (vs), 1459 (m), 1363 (w), 1318 (m), 1229 (w), 1088 (w), 1012 (vw), 744 (s)

MS: MS (ESI, 60 eV): m/z (%) = 373.17 (80) [M^+]

Melting point: 239.7 °C

The analytical data are consistent with the literature.[1]

TRP-PHE-DKP

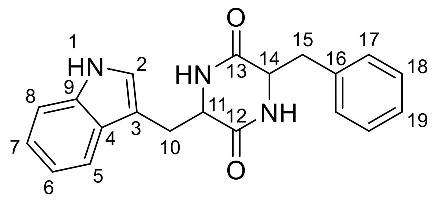

LL-cWF **7a**

^1H NMR (600 MHz, DMSO) δ 10.76 (s, 1H, NH), 7.79 (s, 1H, NH), 7.56 (s, 1H, NH), 7.35 (d, $^3J_{5,6}$ = 7.9 Hz, 1H, H-5), 7.19 (d, $^3J_{8,7}$ = 8.1 Hz, 1H, H-8), 7.07 – 6.98 (m, 3H, H-18 + H-19), 6.93 (dd, $^3J_{6,5+7}$ = 7.5 Hz, 1H, H-6), 6.85 (t, $^3J_{7,6+8}$ = 7.5 Hz, 1H, H-7), 6.83 (s, 1H, H-2), 6.58 – 6.53 (m, 2H, H-17), 3.87 – 3.82 (m, 1H, H-11), 3.73 – 3.69 (m, 1H, H-14), 2.67 (dd, $^3J_{10b,10a}$ = 14.5 Hz, $^3J_{10b,11}$ =4.4 Hz, 1H, Hb-10), 2.39 (dd, $^3J_{10a,10b}$ = 14.5 Hz, $^3J_{10a,11}$ = 5.8 Hz, 1H, Ha-10), 2.31 (dd, $^3J_{15,15a}$ = 13.5 Hz, $^3J_{15b,14}$ = 4.7 Hz, 1H, Hb-15), 1.68 (dd, $^3J_{15a,15b}$ = 13.5 Hz, $^3J_{15a,14}$ = 7.2 Hz, 1H, Ha-15).

^{13}C NMR (151 MHz, DMSO) δ 167.00 (C-12), 166.38 (C-13), 136.57 (C-16), 136.16 (C-4), 129.81 (C-17), 128.18 (C-18), 127.63 (C-9), 126.53 (C-19), 124.57 (C-2), 121.06 (C-6), 118.89 (C-5), 118.60 (C-7), 111.47 (C-8), 108.87 (C-3), 55.73 (C-14), 55.38 (C-11), 39.91 (C-15), 29.79 (C-10).

IR: IR (ATR): ṽ [cm^{-1}] = 3424 (vw), 3324 (m), 2970 (m), 2920 (m), 1739 (s), 1667 (vs). 1457 (s), 1369 (s), 1217 (s), 1092 (w), 744 (s), 699 (m)

MS: MS (ESI, 60 eV): m/z (%) = 334.16 (80) [M^+]

Optical rotation: $[\alpha]_D^{25}$ = *-94 (c 0.23, DMSO)*

Melting point: 278.6 °C

The analytical data are consistent with the literature.[45]

DD-cWF **7b**

^1H NMR (600 MHz, DMSO) δ 10.76 (s, 1H, NH), 7.79 (s, 1H, NH), 7.57 (s, 1H, NH), 7.35 (d, $^3J_{5,6}$ = 7.9 Hz, 1H, H-5), 7.19 (d, $^3J_{8,7}$ = 8.1 Hz, 1H, H-8), 7.07 – 6.99 (m, 3H, H-18 + H-19), 6.94 (dd, $^3J_{6,5+7}$ = 7.5 Hz, 1H, H-6), 6.85 (dd, $^3J_{7,6+8}$ = 7.5 Hz, 1H, H-7), 6.83 (s, 1H, H-2), 6.56 (d,$^3J_{17,18}$ = 7.0 Hz, 2H, H-17), 3.87 – 3.82 (m, 1H, H-11), 3.74 – 3.69 (m, 1H, H-14), 2.67 (dd, $^3J_{10b,10a}$ = 14.5 Hz, $^3J_{10b,11}$ =4.5 Hz, 1H, Hb-10), 2.39 (2.41 – 2.38 (m, 1H, Ha-10), 2.31 (dd, $^3J_{15,15a}$ = 13.5 Hz, $^3J_{15b,14}$ = 4.7 Hz, 1H, Hb-15), 1.68 (dd, $^3J_{15a,15b}$ = 13.4 Hz, $^3J_{15a,14}$ = 7.1 Hz, 1H, Ha-15).

^{13}C NMR (151 MHz, DMSO) δ 166.99 (C-12), 166.37 (C-13), 136.57 (C-16), 136.15 (C-4), 129.81 (C-17), 128.17 (C-18), 127.62 (C-9), 126.56 (C-19), 124.56 (C-2), 121.05 (C-6),

118.88 (C-5), 118.59 (C-7), 111.47 (C-8), 108.86 (C-3), 55.72 (C-14), 55.37 (C-11), 39.91 (C-15), 29.79 (C-10).

IR: IR (ATR): ṽ [cm^{-1}] = 3452 (vw), 3313 (vw), 3025 (w), 2970 (m), 1738 (vs), 1674 (m), 1447 (m), 1366 (s), 1217 (s), 893 (vw), 528 (m)

MS: MS (ESI, 60 eV): m/z (%) = 334.16 (80) [M^+]

Optical rotation: $[\alpha]_D^{25}$ = *86 (c 0.17, DMSO)*

Melting point: 284.7 °C

LD-cWF **7c**

^{1}H NMR (600 MHz, DMSO) δ 10.73 (s, 1H, NH), 7.82 – 7.78 (m, 2H, NH), 7.38 (d, $^3J_{5,6}$ = 7.9 Hz, 1H, H-5), 7.17 (d, $^3J_{8,7}$ = 8.1 Hz, 1H, H-8), 7.12 – 7.02 (m, 3H, H-18 + H-19), 6.98 – 6.94 (m, 2H, H-17), 6.90 (dd, $^3J_{6,5+7}$ = 7.6 Hz, 1H, H-6), 6.88 – 6.85 (m, 1H, H-2), 6.80 (dd, $^3J_{7,6+8}$ = 7.4 Hz, 1H, H-7), 3.27 – 3.22 (m, 2H, H-14 + H-11), 2.99 (dd, $^3J_{10b,10a}$ = 14.6 Hz, $^3J_{10b,11}$ =4.1 Hz, 1H, Hb-10), 2.83 (dd, $^3J_{15b,15a}$ = 13.7 Hz, $^3J_{15b,14}$ = 3.9 Hz, 1H, Hb-15), 2.74 (dd, $^3J_{10a,16b}$ = 14.6 Hz, $^3J_{10a,11}$ =4.6 Hz, 1H, Ha-10), 2.56 (dd, $^3J_{15a,15b}$ = 13.7 Hz, $^3J_{15a,14}$ = 4.9 Hz, 1H, Ha-15).

^{13}C NMR (151 MHz, DMSO) δ 167.85 (C-12), 167.06 (C-13), 136.08 (C-16), 135.95 (C-4), 130.19 (C-17), 128.09 (C-18), 127.69 (C-9), 126.73 (C-19), 124.68 (C-2), 120.98 (C-6), 118.99 (C-5), 118.51 (C-7), 111.26 (C-8), 108.36 (C-3), 54.77 (C-14), 54.69 (C-11), 37.77 (C-15), 28.45 (C-10).

IR: IR (ATR): ṽ [cm^{-1}] = 3324(w), 3197 (w), 3031 (m), 2970 (m), 1738 (s), 1671 (vs), 1452 (s), 1365 (s), 1217 (s), 1098 (w), 749 (w), 519 (w)

MS: MS (ESI, 60 eV): m/z (%) = 334.16 (80) [M^+]

Optical rotation: $[\alpha]_D^{25}$ = *-12,4 (c 0.21, DMSO)*

Melting point: 251.5 °C

DL-cWF **7c**

^{1}H NMR (600 MHz, DMSO) δ 10.87 (s, 1H, NH), 7.96 – 7.92 (m, 2H, NH), 7.51 (d, $^3J_{5,6}$ = 7.9 Hz, 1H, H-5), 7.30 (d, $^3J_{8,7}$ = 8.1 Hz, 1H, H-8), 7.25 – 7.13 (m, 3H, H-18 + H-19), 7.11 – 7.07 (m, 2H, H-17), 7.03 (dd, $^3J_{6,5+7}$ = 7.6 Hz, 1H, H-6), 7.00 – 6.99 (m, 1H, H-2), 6.92 (dd, $^3J_{7,6+8}$ = 7.5 Hz, 1H, H-7), 3.41 – 3.34 (m, 2H, H-14 + H-11), 3.12 (dd, $^3J_{10b,10a}$ = 14.6 Hz, $^3J_{10b,11}$ =4.1 Hz, 1H, Hb-10), 2.96 (dd, $^3J_{15b,15a}$ = 13.7 Hz, $^3J_{15b,14}$ = 3.8 Hz, 1H, Hb-15), 2.85 (dd, $^3J_{10a,16b}$ = 14.6 Hz, $^3J_{10a,11}$ =4.6 Hz, 1H, Ha-10), 2.68 (dd, $^3J_{15a,15b}$ = 13.7 Hz, $^3J_{15a,14}$ = 4.9 Hz, 1H, Ha-15).

^{13}C NMR (151 MHz, DMSO) δ 167.65 (C-12), 166.87 (C-13), 136.04 (C-16), 135.84 (C-4), 130.09 (C-17), 127.95 (C-18), 127.60 (C-9), 126.57 (C-19), 124.55 (C-2), 120.81 (C-6), 118.89 (C-5), 118.35 (C-7), 111.13 (C-8), 108.28 (C-3), 54.65 (C-14), 54.65 (C-11), 37.64 (C-15), 28.35 (C-10).

IR: IR (ATR): ṽ [cm^{-1}] = 3258 (m), 3031 (m), 2970 (m), 2920 (m), 2859 (vw), 1738 (vs), 1668 (vs), 1452 (m), 1365 (s), 1217 (s), 1092 (w), 744 (w), 705 (w), 528 (w)

MS: MS (ESI, 60 eV): m/z (%) = 334.16 (80) [M^+]

Optical rotation: $[\alpha]_D^{25}$ = *4 (c 0.57, DMSO)*

Melting point: 241.2 °C

The analytical data are consistent with the literature.[46]

TRP-TYR-DKP

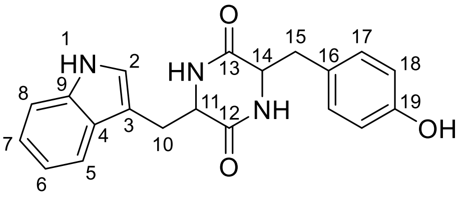

LL-cWY **6a**

^{1}H NMR (600 MHz, DMSO) δ 10.76 (s, 1H, NH), 7.70 (s, 1H, NH), 7.49 (s, 1H, NH), 7.34 (d, $^{3}J_{5,6}$ = 7.9 Hz, 1H, H-5), 7.18 (d, $^{3}J_{8,7}$ = 8.1 Hz, 1H, H-8), 6.93 (dd, $^{3}J_{6,5+7}$ = 7.5 Hz, 1H, H-6), 6.87 – 6.81 (m, 2H, H-2 + H-7), 6.44 (d, $^{3}J_{18,17}$ = 8.3 Hz, 2H, H-18), 6.37 (d, $^{3}J_{17,18}$ = 8.3 Hz, 2H, H-17), 3.83 – 3.78 (m, 1H, H-11), 3.66 – 3.61 (m, 1H, H-14), 3.03 (s, 1H, OH), 2.66 (dd, $^{3}J_{10b,10a}$ = 14.4 Hz, $^{3}J_{10b,11}$ =4.4 Hz 1H, Hb-10), 2.31 (dd, $^{3}J_{15,15a}$ = 14.4 Hz, $^{3}J_{15b,14}$ = 6.3 Hz, 1H, Hb-15), 2.27 (dd, $^{3}J_{10a,10b}$ = 13.6 Hz, $^{3}J_{10a,11}$ = 4.6 Hz Hz, 1H, Ha-10), 1.63 (dd, $^{3}J_{15a,15b}$ = 13.5 Hz, $^{3}J_{15a,14}$ = 7.1 Hz, 1H, Ha-15).

^{13}C NMR (151 MHz, DMSO) δ 166.95 (C-12), 166.46 (C-13), 156.07 (C-19), 136.18 (C-4), 130.82 (C-17), 127.58 (C-9), 126.50 (C-16), 124.52 (C-2), 121.04 (C-6), 118.85 (C-5), 118.58 (C-7), 115.03 (C-18), 111.46 (C-8), 108.96 (C-3), 55.99 (C-14), 55.35 (C-11), 19.91 (C-15), 30.04 (C-10).

IR: IR (ATR): ṽ [cm^{-1}] = 3313 (m), 3202 (m), 3056 (w), 2926 (vw), 1671 (vs), 1619 (vw), 1516 (m), 1457 (s), 1358 (vw), 1232 (m), 1104 (w), 821 (w), 745 (m)

MS: MS (ESI, 60 eV): m/z (%) = 350.15 (80) [M^{+}]

Optical rotation: $[\alpha]_D^{25}$ = *-106 (c 0.29, DMSO)*

Melting point: 271.3 °C

The analytical data are consistent with the literature.[45]

DD-cWY **6b**

^{1}H NMR (600 MHz, DMSO) δ 10.75 (s, 1H, NH), 7.69 (s, 1H, NH), 7.49 (s, 1H, NH), 7.34 (d, $^3J_{5,6}$ = 7.9 Hz, 1H, H-5), 7.18 (d, $^3J_{8,7}$ = 8.1 Hz, 1H, H-8), 6.93 (dd, $^3J_{6,5+7}$ = 7.5 Hz, 1H, H-6), 6.87 – 6.81 (m, 2H, H-2 + H-7), 6.44 (d, $^3J_{18,17}$ = 8.1 Hz, 2H, H-18), 6.37 (d, $^3J_{17,18}$ = 8.1 Hz, 2H, H-17), 3.83 – 3.78 (m, 1H, H-11), 3.66 – 3.61 (m, 1H, H-14), 3.03 (s, 1H, 1H), 2.66 (dd, $^3J_{10b,10a}$ = 14.5 Hz, $^3J_{10b,11}$ =4.4 Hz 1H, Hb-10), 2.31 (dd, $^3J_{15,15a}$ = 14.4 Hz, $^3J_{15b,14}$ = 6.3 Hz, 1H, Hb-15), 2.27 (dd, $^3J_{10a,10b}$ = 13.6 Hz, $^3J_{10a,11}$ = 4.6 Hz Hz, 1H, Ha-10), 1.62 (dd, $^3J_{15a,15b}$ = 13.6 Hz, $^3J_{15a,14}$ = 7.1 Hz, 1H Ha-15).

^{13}C NMR (151 MHz, DMSO) δ 166.96 (C-12), 166.47 (C-13), 156.08 (C-19), 136.19 (C-4), 130.83 (C-17), 127.59 (C-9), 126.50 (C-16), 124.53 (C-2), 121.04 (C-6), 118.86 (C-5), 118.58 (C-7), 115.03 (C-18), 111.46 (C-8), 108.96 (C-3), 55.99 (C-14), 55.35 (C-11), 39.93 (C-15), 30.04 (C-10).

IR: IR (ATR): ṽ [cm^{-1}] = 3300 (m), 3208 (w), 3092 (w), 2926 (vw), 1704 (s), 1681 (vs), 1613 (w), 1519 (m), 1467 (m), 1358 (m), 1331 (m), 1233 (s), 1104 (m), 777 (w), 733 (s)

MS: MS (ESI, 60 eV): m/z (%) = 350.15 (80) [M$^+$]

Optical rotation: $[\alpha]_D^{25}$ = *102 (c 0.22, DMSO)*

Melting point: 267.3 °C

LD-cWY **6c**

^{1}H NMR (600 MHz, DMSO) δ 7.46 (d, $^{3}J_{5,6}$ = 8.0 Hz, 1H, H-5), 7.28 (d, $^{3}J_{8,7}$ = 8.1 Hz, 1H, H-8), 7.07 – 7.01 (m, 1H, H-6), 6.98 (s, 1H, H-2), 6.97 – 6.92 (m, 1H, H-7), 6.87 – 6.82 (m, 2H, H-17), 6.64 – 6.58 (m, 2H, H-18), 3.47 (t, J = 4.6 Hz, 1H, H-11), 3.35 – 3.33 (m, 1H, H-14), 3.23 – 3.18 (m, 1H, Hb-10), 3.02 (dd, $^{3}J_{10a,10b}$ = 14.8 Hz, $^{3}J_{10a,11}$ = 4.4 Hz Hz, 1H, Ha-10), 2.90 (dd, $^{3}J_{15,15a}$ = 14.2 Hz, $^{3}J_{15b,14}$ = 4.2 Hz, 1H, Hb-15), 2.66 (dd, $^{3}J_{15a,15b}$ = 14.1 Hz, $^{3}J_{15a,14}$ = 4.6 Hz, 1H Ha-15).

^{13}C NMR (151 MHz, MeOD) δ 170.70 (C-12), 170.04 (C-13), 157.78 (C-19), 137.95 (C-4), 132.26 (C-17), 128.76 (C-9), 126.88 (C-16), 125.77 (C-2), 122.50 (C-6), 120.07 (C-5), 119.78 (C-7), 116.16 (C-18), 112.15 (C-8), 109.09 (C-3), 56.77 (C-14), 56.29 (C-11), 38.84 (C-15), 30.24 (C-10).

IR: IR (ATR): ṽ [cm^{-1}] = 3319 (s), 3191 (m), 3047 (w), 2926 (vw), 1671 (vs), 1613 (w), 1516 (m), 1330 (m), 1231 (s), 1103 (w), 827 (w), 745 (m)

MS: MS (ESI, 60 eV): m/z (%) = 350.15 (80) [M^{+}]

Optical rotation: $[\alpha]_D^{25}$ = *-21 (c 0.41, DMSO)*

Melting point: 237.5 °C

DL-cWY **6d**

^{1}H NMR (600 MHz, MeOD) δ 7.47 (d, $^{3}J_{5,6}$ = 8.0 Hz, 1H, H-5), 7.29 (d, $^{3}J_{8,7}$ = 8.1 Hz, 1H, H-8), 7.07 – 7.03 (m, 1H, H-6), 6.99 (s, 1H, H-2), 6.98 – 6.95 (m, 1H, H-7), 6.88 – 6.83 (m, 2H, H-17), 6.64 – 6.57 (m, 2H, H-18), 3.50 – 3.45 (m, 1H, H-11), 3.35 – 3.33 (m, 1H, H-14), 3.21 (dd, $^{3}J_{10b,10a}$ = 14.8 Hz, $^{3}J_{10b,11}$ = 4.8 Hz 1H, Hb-10), 3.02 (dd, $^{3}J_{10a,10b}$ = 14.8 Hz, $^{3}J_{10a,11}$ = 4.4 Hz Hz, 1H, Ha-10), 2.91 (dd, $^{3}J_{15,15a}$ = 14.1 Hz, $^{3}J_{15b,14}$ = 4.2 Hz, 1H, Hb-15), 2.66 (dd, $^{3}J_{15a,15b}$ = 14.1 Hz, $^{3}J_{15a,14}$ = 4.6 Hz, 1H Ha-15).

^{13}C NMR (151 MHz, MeOD) δ 170.71 (C-12), 170.04 (C-13), 157.77 (C-19), 137.98 (C-4), 132.22 (C-17), 128.76 (C-9), 126.94 (C-16), 125.76 (C-2), 122.52 (C-6), 120.09 (C-5), 119.76 (C-7), 116.22 (C-18), 112.17 (C-8), 109.15 (C-3), 56.80 (C-14), 56.29 (C-11), 38.89 (C-15), 30.26 (C-10).

IR: IR (ATR): ṽ [cm^{-1}] = 3324 (m), 3196 (m), 3056 (m), 2915 (w), 1670 (vs), 1615 (w), 1516 (m), 1467 (s), 1103 (w), 1015 (vw), 827 (w), 745 (m)

MS: MS (ESI, 60 eV): m/z (%) = 350.15 (80) [M$^+$]

Optical rotation: $[\alpha]_D^{25}$ = 7 (c 5.4, DMSO)

Melting point: 234.9 °C

TRP-HIS-DKP

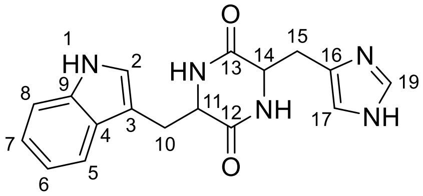

LL-cWH **8a**

^{1}H NMR (600 MHz, MeOD) δ 8.26 (s, 1H, NH), 8.01 (s, 1H, H-19), 7.61 (d, $^3J_{5,6}$ = 7.9 Hz, 1H, H-5), 7.34 (d, $^3J_{8,7}$ = 8.1 Hz, 1H, H-8), 7.13 (dd, $^3J_{6,5+7}$ = 7.5 Hz, 1H, H-6), 7.08 (s, 1H, H-2), 7.04 (dd, $^3J_{7,6+8}$ = 7.4 Hz, 1H, H-7), 5.62 – 5.59 (m, 1H, H-17), 4.26 – 4.21 (m, 1H, H-11), 3.83 – 3.78 (m, 1H, H-14), 3.36 (dd, $^3J_{10b,10a}$ = 14.8 Hz, $^3J_{10b,11}$ = 3.7 Hz, 1H, Hb-10), 3.05 (dd, $^3J_{10a,10b}$ = 14.7 Hz, $^3J_{10a,11}$ = 4.5 Hz, 1H, Ha-10), 2.32 (dd, $^3J_{15b,15a}$ = 14.5 Hz, $^3J_{15b,14}$ = 4.3 Hz, 1H, Hb-15), 0.99 (dd, $^3J_{15a,15b}$ = 14.5 Hz, $^3J_{15a,14}$ = 10.0 Hz, 1H, Ha-15).

13C NMR (151 MHz, MeOD) δ 169.78 (C-13), 168.58 (C-12), 137.83 (C-4), 135.76 (C-19), 131.16 (C-16), 129.28 (C-9), 126.40 (C-2), 122.72 (C-6), 120.42 (C-5), 120.34 (C-7),

118.91 (C-17), 112.64 (C-8), 109.59 (C-3), 57.33 (C-11), 55.30 (C-14), 31.79 (C-15), 30.73 (C-10).

IR: IR (ATR): ṽ [cm-1] = 3214 (m), 3134 (m), 3059 (w), 2926 (w), 2870 (w), 1667 (vs), 1584 (s), 1457 (s), 1340 (s), 1225 (m), 1094 (m), 747 (s), 633 (w)

MS: MS (ESI, 60 eV): m/z (%) = 324.15 (80) [M+]

Optical rotation: $[\alpha]_D^{25}$ = -72 (c 0.22, DMSO)

Melting point: 304.2 °C

DD-cWH **8b**

^{1}H NMR (600 MHz, MeOD) δ 7.58 (d, $^3J_{5,6}$ = 8.0 Hz, 1H, H-5), 7.44 – 7.41 (m, 1H, H-19), 7.32 (d, $^3J_{8,7}$ = 8.1 Hz, 1H, H-8), 7.09 (dd, $^3J_{6,5+7}$ = 8.2 Hz, 1H, H-6), 7.05 (s, 1H, H-2), 7.00 (dd, $^3J_{7,6+8}$ = 7.4 Hz, 1H, H-7), 5.75 (s, 1H, H-17), 4.21 – 4.16 (m, 1H, H-11), 3.86 – 3.80 (m, 1H, H-14), 3.20 (dd, $^3J_{10b,10a}$ = 14.7 Hz, $^3J_{10b,11}$ = 4.5 Hz, 1H, Hb-10), 3.04 (dd, $^3J_{10a,10b}$ = 14.7 Hz, $^3J_{10a,11}$ = 4.5 Hz, 1H, Ha-10), 2.43 – 2.37 (m, 1H, Hb-15), 1.02 (dd, $^3J_{15a,15b}$ = 14.3 Hz, $^3J_{15a,14}$ = 9.9 Hz, 1H, Ha-15).

13C NMR (151 MHz, MeOD) δ 169.73 (C-13), 169.23 (C-12), 137.88 (C-4), 136.52 (C-19), 132.6 (C-16), 129.18 (C-9), 126.14 (C-2), 122.65 (C-6), 120.27 (C-5), 120.27 (C-7), 117.81 (C-17), 112.65 (C-8), 109.52 (C-3), 57.25 (C-11), 55.93 (C-14), 31.12 (C-15), 30.92 (C-10).

IR: IR (ATR): ṽ [cm-1] = 3220 (m), 3021 (m), 2971 (m), 1738 (vs), 1667 (vs), 1589 (m), 1456 (s), 1362 (vs), 1224 (vs), 1091 (w), 743 (m=, 533 (vw)

MS: MS (ESI, 60 eV): m/z (%) = 324.15 (80) [M+]

Optical rotation: $[\alpha]_D^{25}$ = 79 (c 0.3, DMSO)

Melting point: 301.3 °C

LD-cWH **8c**

^{1}H NMR (600 MHz, MeOD) δ 7.51 (d, $^3J_{5,6}$ = 8.0 Hz, 1H, H-5), 7.48 (s, 1H, H-19), 7.26 (d, $^3J_{8,7}$ = 8.1 Hz, 1H, H-8), 7.02 (dd, $^3J_{6,5+7}$ = 7.6 Hz, 1H, H-6), 6.99 (s, 1H, H-2), 6.93 (dd, $^3J_{7,6+8}$ = 7.4 Hz, 1H, H-7), 6.59 (s, 1H, H-17), 3.90 (t, $^3J_{11,10}$ = 4.5 Hz, 1H, H-11), 3.34 – 3.29 (m, 1H, Hb-10), 3.06 (dd, $^3J_{10a,10b}$ = 14.8 Hz, $^3J_{10a,11}$ =4.4 Hz, 1H, Ha-10), 3.04 – 3.00 (m, 1H, H-14), 2.84 (dd, $^3J_{15b,15a}$ = 15.2 Hz, $^3J_{15b,14}$ = 5.5 Hz, 1H, Hb-15), 2.74 (dd, $^3J_{15a,15b}$ = 15.2 Hz, $^3J_{15a,14}$ = 4.4 Hz, 1H, Ha-15).

13C NMR (151 MHz, MeOD) δ 170.90 (C-12), 169.86 (C-13), 137.96 (C-4), 136.24 (C-19), 128.78 (C-9), 125.91 (C-2), 122.57 (C-6), 120.15 (C-7), 119.71 (C-5), 119.21 (C-17), 112.24 (C-8), 109.18 (C-3), 57.22 (C-11), 55.23 (C-14), 30.81 (C-10), 30.69 (C-15).

IR: IR (ATR): ṽ [cm-1] = 3225 (s), 2920 (m), 2848 (w), 1740 (m), 1667 (vs), 1585 (m), 1452 (s), 1364 (s), 1225 (m), 1092 (w), 749 (m), 622 (vw)

MS: MS (ESI, 60 eV): m/z (%) = 324.15 (80) [M+]

Optical rotation: $[\alpha]_D^{25}$ = - 6 (c 0.98, DMSO)

Melting point: 212.7 °C

DL-cWH **8d**

^{1}H NMR (600 MHz, MeOD) δ 7.53 (d, $^3J_{5,6}$ = 8.0 Hz, 1H, H-5), 7.51 (s, 1H, H-19), 7.29 (d, $^3J_{8,7}$ = 8.1 Hz, 1H, H-8), 7.04 (dd, $^3J_{6,5+7}$ = 7.6 Hz, 1H, H-6), 7.01 (s, 1H, H-2), 6.96 (dd, $^3J_{7,6+8}$ = 7.4 Hz, 1H, H-7), 6.61 (s, 1H, H-17), 3.92 (t, $^3J_{11,10}$ = 4.4 Hz, 1H, H-11), 3.36 – 3.32 (m, 1H, Hb-10), 3.09 (dd, $^3J_{10a,10b}$ = 14.8 Hz, $^3J_{10a,11}$ =4.4 Hz, 1H, Ha-10), 3.06 – 3.01 (m, 1H, H-14), 2.86 (dd, $^3J_{15b,15a}$ = 15.2 Hz, $^3J_{15b,14}$ = 5.5 Hz, 1H, Hb-15), 2.76 (dd, $^3J_{15a,15b}$ = 15.2 Hz, $^3J_{15a,14}$ = 4.4 Hz, 1H, Ha-15).

13C NMR (151 MHz, MeOD) δ 170.92, (C-12), 169.86 (C-13), 137.98 (C-4), 136.23 (C-19), 128.79 (C-9), 125.93 (C-2), 122.58 (C-6), 120.15 (C-7), 119.71 (C-5), 119.19 (C-17), 112.23 (C-8), 109.17 (C-3), 57.24 (C-11), 55.23 (C-14), 30.83 (C-10), 30.68 (C-15).

IR: IR (ATR): ṽ [cm-1] = (3230 (w), 2929 (w), 1740 (m), 1669 (s), 1456 (m), 1364 (m) 1230 (m), 1098 (w), 745 (m), 622 (w).

MS: MS (ESI, 60 eV): m/z (%) = 324.15 (80) [M+]

Optical rotation: $[\alpha]_D^{25}$ = 1 (c 0.70, DMSO)

Melting point: 202.6 °C

TRP-ALA-DKP

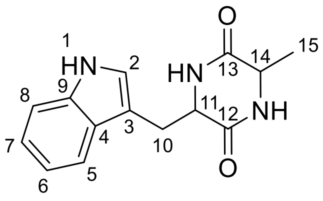

LL-cWA **9**

^{1}H NMR (600 MHz, DMSO) δ 10.87 (s, 1H, NH), 8.01 – 7.98 (m, 1H, NH), 7.90 – 7.86 (m, 1H, NH), 7.53 (d, $^3J_{5,6}$ = 7.9 Hz, 1H, H-5), 7.27 (d, $^3J_{8,7}$ = 8.1 Hz, 1H, H-8), 7.04 – 6.97 (m,

2H, H-2 + H-6), 6.91 (dd, $^3J_{7,6+8}$ = 7.5 Hz, 1H, H-7), 4.10 – 4.05 (m, 1H, H-11), 3.59 – 3.52 (m, 1H, H-14), 3.21 (dd, $^3J_{10b,10a}$ = 14.4 Hz, $^3J_{10b,11}$ =4.1 Hz, 1H, Hb-10), 2.98 (dd, $^3J_{10a,10b}$ = 14.5 Hz, $^3J_{10a,11}$ =4.6 Hz, 1H, Ha-10), 0.38 (d, $^3J_{15,14}$ = 6.9 Hz, 3H, H-15).

13C NMR (151 MHz, DMSO) δ 167.73 (C-13), 166.75 (C-12), 135.79 (C-4), 127.80 (C-9), 124.56 (C-2), 120.80 (C-6), 118.98 (C-5), 118.38 (C-7), 111.10 (C-8), 108.50 (C-3), 55.40 (C-11), 49.78 (C-14), 28.84 (C-10), 19.55 (C-15).

IR: IR (ATR): ṽ [cm-1] = 3410 (w), 3187 (w), 3049 (w), 2919 (w), 1656 (s), 1454 (m), 1324 (m), 1094 (w), 826 (m), 742 (s), 504 (m).

MS: MS (ESI, 60 eV): m/z (%) = 258.12 (80) [M+]

Optical rotation: $[\alpha]_D^{25}$ = - 17 (c 0.22, DMSO)

Melting point: 284.0 °C

The analytical data are consistent with the literature.[47]

TRP-VAL-DKP

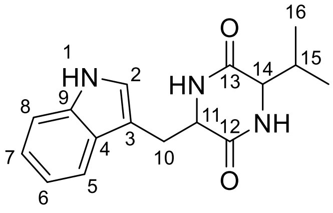

LL-cWV **10**

^{1}H NMR (600 MHz, DMSO) δ 10.83 (s, 1H, NH), 7.99 (s, 1H, NH), 7.87 (s, 1H, NH), 7.58 (d, $^3J_{5,6}$ = 7.9 Hz, 1H, H-5), 7.27 (d, $^3J_{8,7}$ = 8.1 Hz, 1H, H-8), 7.07 – 7.04 (m, 1H, H-2), 7.00 (d, $^3J_{6,7}$ = 7.5 Hz, 1H, H-6), 6.92 (dd, $^3J_{7,6+8}$ = 7.5 Hz, 1H, H-7), 4.15 – 4.10 (m, 1H, H-11), 3.49 – 3.45 (m, 1H, H-14), 3.20 (dd, $^3J_{10b,10a}$ = 14.4 Hz, $^3J_{10b,11}$ =4.1 Hz, 1H, Hb-10),), 3.05

(dd, $^3J_{10a,10b}$ = 14.5 Hz, $^3J_{10a,11}$ =4.6 Hz, 1H, Ha-10), 1.67 – 1.58 (m, 1H, H-15), 0.58 (d, $^3J_{16a,15}$ = 7.0 Hz, 3H, Ha-16), 0.15 (d, $^3J_{16b,15}$ = 6.8 Hz, 3H, Hb-16).

13C NMR (151 MHz, DMSO) δ 167.36 (C-13), 166.29 (C-12), 136.02 (C-4), 127.94 (C-9), 124.49 (C-2), 120.67 (C-6), 118.91 (C-5), 118.18 (C-7), 111.01 (C-8), 108.84 (C-3), 59.29 (C-14), 55.18 (C-11), 31.10 (C-15), 28.75 (C-10), 18.33 (C-16a), 16.11 (C-16b).

IR: IR (ATR): ṽ [cm-1] = 3325 (w), 3187 (w), 2953 (w), 2919 (w), 1736 (m), 1661 (s), 1462 (m), 1339 (w), 1224 (w), 1102 (w), 849 (w), 741 (m), 548 (m).

MS: MS (ESI, 60 eV): m/z (%) = 286.16 (80) [M+]

Optical rotation: $[\alpha]_D^{25}$ = - 26 (c 0.30, DMSO)

Melting point: 291.4 °C

The analytical data are consistent with the literature.[48]

TRP-LEU-DKP

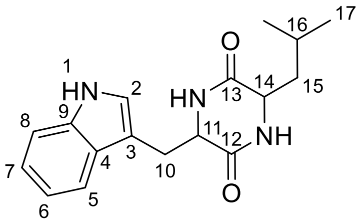

LL-cWL **11**

^{1}H NMR (600 MHz, DMSO) δ 10.95 – 10.92 (m, 1H, NH), 8.08 – 8.05 (m, 1H, NH), 7.98 – 7.94 (m, 1H, NH), 7.55 (d, $^3J_{5,6}$ = 7.9 Hz, 1H, H-5), 7.30 (d, $^3J_{8,7}$ = 8.1 Hz, 1H, H-8), 7.05 – 6.99 (m, 1H, H-2, H-6), 6.92 (dd, $^3J_{7,6+8}$ = 7.4 Hz, 1H, H-7), 4.11 – 4.06 (m, 1H, H-11), 3.42 – 3.37 (m, 1H, H-14), 3.26 (dd, $^3J_{10b,10a}$ = 14.4 Hz, $^3J_{10b,11}$ =4.0 Hz, 1H, Hb-10),), 2.98 (dd, $^3J_{10a,10b}$ = 14.5 Hz, $^3J_{10a,11}$ =4.7 Hz, 1H, Ha-10), 1.25 – 1.15 (m, 1H, H-16), 0.67 – 0.59 (m,

1H, Hb-15), 0.52 (d, $^3J_{17b,16}$ = 6.5 Hz, 3H, H-17b), 0.42 (d, $^3J_{17a,16}$ = 6.6 Hz, 3H, H-17a), 0.01 – -0.06 (m, 1H, 15a).

13C NMR (151 MHz, DMSO) δ 167.48 (C-13), 167.11 (C-12), 135.93 (C-4), 127.80 (C-9), 124.65 (C-2), 120.76 (C-6), 119.01 (C-5), 118.34 (C-7), 111.12 (C-8), 108.51 (C-3), 55.54 (C-14), 52.37 (C-11), 43.69 (C-15), 29.12 (C-10), 22.87 (C-16), 22.69 (C-17a), 21.28 (C-17b).

IR: IR (ATR): ṽ [cm-1] = 3325 (w), 3192 (w), 2956 (w), 1661 (s), 1457 (m), 1327 (w), 1231 (w), 1093 (w), 1011 (w), 741 (m), 458 (w).

MS: MS (ESI, 60 eV): m/z (%) = 300.17 (80) [M+]

Optical rotation: $[\alpha]_D^{25}$ = - 7 (c 0.30, DMSO)

Melting point: 247.0 °C

The analytical data are consistent with the literature.[46]

Benzodiazepinedione

TRP-benzodiazepinedione

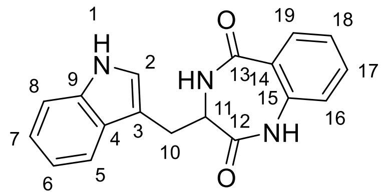

L-cWBenz **12a**

^{1}H NMR (600 MHz, DMSO) δ 10.86 – 10.82 (m, 1H, NH), 10.41 (s, 1H NH), 8.42 – 8.38 (m, 1H, NH), 7.62 (d, $^3J_{19,18}$ = 7.9, 1H, H-19), 7.50 – 7.44 (m, 2H, H-18 + H-5), 7.29 (d, $^3J_{8,7}$ = 8.1 Hz, 1H, H-8), 7.23 – 7.19 (m, 1H, H-2), 7.15 (dd, $^3J_{17,16+18}$ = 7.5 Hz, 1H, H-17), 7.08 (d,

$^3J_{16,17}$ = 8.1 Hz, 1H, H-16), 7.00 (dd, $^3J_{6,7+5}$ = 7.5 Hz, 1H, H-6), 6.88 (d, $^3J_{7,8+9}$= 7.4 Hz, 1H, H-7), 3.85 (dd, $^3J_{11,10a}$ = 9.3 Hz, $^3J_{11,10b}$ = 5.6 Hz, 1H, H-11), 3.21 (dd, $^3J_{10b,10a}$ = 14.9 Hz, $^3J_{10b,11}$ = 5.2 Hz, 1H, Hb-10), 2.97 (dd, $^3J_{10a,10b}$ = 14.9 Hz, $^3J_{10a,11}$ = 9.4 Hz, 1H, Ha-10)

^{13}C NMR (151 MHz, DMSO) δ 171.55 (C-12), 167.60 (C-13), 136.80 (C-14), 136.06 (C-4), 132.24 (C-18), 130.39 (C-19), 126.96 (C-9), 126.24 (C-15), 124.32 (C-2), 123.90 (C-17), 120.97 (C-16), 120.93 (C-7), 118.34 (C-5), 118.25 (C-7), 111.40 (C-8), 109.65 (C-3), 52.79 (C.11), 23.54 (C-10).

IR: IR (ATR): ṽ [cm^{-1}] = 3272 (w), 3019 (w), 2973 (w), 1736 (s), 1646 (m), 1439 (w), 1366 (m), 1217 (m), 741 (w), 527 (w).

MS: MS (ESI, 60 eV): m/z (%) = 306.12 (80) [M$^+$]

Optical rotation: $[\alpha]_D^{25}$= 193 (c 0.26, DMSO*)*

Melting point: 247.4 °C

The analytical data are consistent with the literature.[44, 49]

D-cWBenz **12b**

^{1}H NMR (600 MHz, DMSO) δ 10.85 – 10.82 (m, 1H, NH), 10.41 (s, 1H NH), 8.42 – 8.38 (m, 1H, NH), 7.62 (d, $^3J_{19,18}$ = 7.8, 1H, H-19), 7.49 – 7.43 (m, 2H, H-18 + H-5), 7.29 (d, $^3J_{8,7}$ = 8.1 Hz, 1H, H-8), 7.23 – 7.19 (m, 1H, H-2), 7.14 (dd, $^3J_{17,16+18}$ = 7.6 Hz, 1H, H-17), 7.07 (d, $^3J_{16,17}$ = 8.1 Hz, 1H, H-16), 7.00 (dd, $^3J_{6,7+5}$ = 7.5 Hz, 1H, H-6), 6.87 (d, $^3J_{7,8+9}$= 7.5 Hz, 1H, H-7), 3.85 (dd, $^3J_{11,10a}$ = 9.3 Hz, $^3J_{11,10b}$ = 5.6 Hz, 1H, H-11), 3.20 (dd, $^3J_{10b,10a}$ = 15.0 Hz, $^3J_{10b,11}$ = 5.2 Hz, 1H, Hb-10), 2.97 (dd, $^3J_{10a,10b}$ = 14.9 Hz, $^3J_{10a,11}$ = 9.4 Hz, 1H, Ha-10)

^{13}C NMR (151 MHz, DMSO) δ 171.56 (C-12), 167.62 (C-13), 136.80 (C-14), 136.06 (C-4), 132.25 (C-18), 130.39 (C-19), 126.97 (C-9), 126.24 (C-15), 124.33 (C-2), 123.91 (C-17), 120.98 (C-16), 120.94 (C-7), 118.35 (C-5), 118.26 (C-7), 111.41 (C-8), 109.66 (C-3), 52.80 (C.11), 23.55 (C-10).

IR: IR (ATR): ṽ [cm^{-1}] = 3263 (w), 3066 (w), 2919 (w), 1738 (m), 1647 (s), 1480 (m), 1402 (m), 1365 (m), 1228 (m), 742 (m), 529 (w).

MS: MS (ESI, 60 eV): m/z (%) = 306.12 (80) [M^+]

Optical rotation: $[\alpha]_D^{25}$= -195 (c 0.29, DMSO*)*

Melting point: 250.0 °C

Single C3-methylated *LL*-cWW 13a

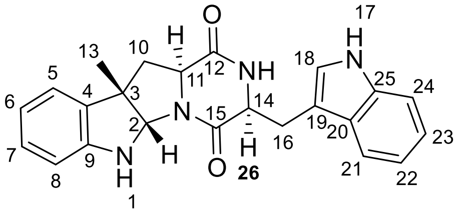

^1H NMR (600 MHz, $CDCl_3$) δ 8.35 (s, 1H, NH), 7.56 (d, $^3J_{24,23}$ = 8.0 Hz, 1H, 24-H), 7.36 (d, $^3J_{21,22}$ = 8.2 Hz, 1H, 21-H), 7.24 – 7.18 (m, 1H, H-Ar), 7.16 – 7.06 (m, 4H, H-Ar), 6.78 (t, *J* = 7.4, 1H, H-Ar), 6.63 (d, $^3J_{5,6}$ = 7.4 Hz, 1H, 5-H), 5.89 (s, 1H, NH), 5.26 (s, 1H, 2-H), 5.13 (s, 1H, NH), 4.30 (dd, $^3J_{14,16a}$ = 10.6 Hz, $^3J_{14,16b}$ = 3.8 Hz, 1H, 14-H), 3.95 (dd, $^3J_{11,10a}$= 11.3 Hz, $^3J_{11,10b}$ = 6.1 Hz, 1H, 11-H), 3.67 (dd, $^2J_{16b,16a}$ = 15.2 Hz, $^3J_{16b,14}$ 3.8 Hz, 1H, 16b-H), 3.03 (dd, $^2J_{16a,16b}$ = 15.0 Hz, $^3J_{16a,14}$ = 10.6 Hz, 1H, 16a-H), 2.65 (dd, $^2J_{10b,10a}$ = 12.7 Hz, $^3J_{10b,11}$ = 6.1 Hz, 1H, Hb-10), 2.03 (dd, $^2J_{10a,10b}$ = 12.7 Hz, $^3J_{10a,11}$ = 11.3 Hz, 1H, Ha-10), 1.37 (s, 3H, 13-H).

^{13}C NMR (151 MHz, $CDCl_3$) δ 169.01 (C-15), 166.21 (C-12), 148.61 (C-9), 136.69 (C-25), 132.27 (C-4), 128.83 (C-18), 126.80 (C-20), 123.64 (C-18), 122.91, 122.88, 120.13, 119.71 (C-Ar), 118.67 (C-24), 111.68 (C-21), 109.57 (C-19), 109.52 (C-8), 81.57 (C-2), 59.16 (C-11), 54.88 (C-14), 51.71 (C-3), 40.93 (C-10), 27.33 (C-16), 24.47 (C-13).

IR: IR (ATR): ṽ [cm^{-1}] = 3316 (m), 1664 (vs), 1457 (s), 1321 (m), 1216 (w), 1097 (w), 1062 (w), 745 (s).

MS: MS (ESI, 60 eV): m/z (%) = 387.18 [M+]

Optical rotation: $[\alpha]_D^{20}$ = -140 (c 1.0, MeOH)

The analytical data are consistent with the literature.[1]

Single C3-methylated *DD*-cWW 13b

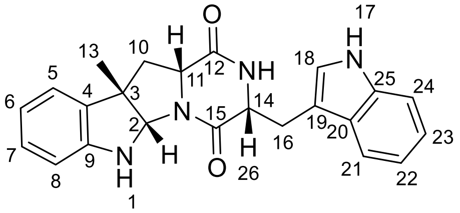

^{1}H NMR (600 MHz, $CDCl_3$) δ 8.26 (s, 1H, NH), 7.58 (d, $^3J_{24,23}$ = 7.9 Hz, 1H, 24-H), 7.33 (d, $^3J_{21,22}$ = 8.2 Hz, 1H, 21-H), 7.24 – 7.18 (m, 1H, H-Ar), 7.15 – 7.07 (m, 3H, H-Ar), 7.02 – 6.98 (m, H, NH), 6.78 (t, J = 7.5 Hz, 1H, H-Ar), 6.61 (d, $^3J_{5,6}$ = 7.7 Hz, 1H, 5-H), 5.95 (s, 1H, NH), 5.41 (d, $^3J_{2,NH}$ = 3.1 Hz, 1H, 2-H), 4.37 (dd, $^3J_{14,16a}$ = 11.0 Hz, $^3J_{14,16b}$ = 3.8 Hz, 1H, 14-H), 4.35 – 4.30 (m, 1H, 11-H), 3.72 (dd, $^2J_{16b,16a}$ = 15.0 Hz, $^3J_{16b,14}$ = 3.7 Hz, 1H, 16b-H), 2.98 (dd, $^2J_{16a,16b}$ = 15.1 Hz, $^3J_{16a,14}$ =11.0 Hz, 1H, 16a-H), 2.36 (d, $^3J_{10,11}$ = 8.7 Hz, 2H, 10-H), 1.48 (s, 3H, 13-H).

^{13}C NMR (151 MHz, $CDCl_3$) δ 168.22 (C-15), 167.26 (C-12), 147.28 (C-9), 136.74 (C-25), 133.51 (C-4), 128.67 (C-18), 126.77 (C-20), 123.63, 122.83, 122.61, 120.06, 119.42 (C-Ar),

118.55 (C-24), 111.71 (C-21), 109.9´65 (C-19), 109.58 (C-5), 84.09 (C-2), 58.41 (C-14), 54.81 (C-11), 51.32 (C-3), 41.03 (C-10), 26.81 (C-16), 23.57 (C-13).

IR: IR (ATR): ṽ [cm^{-1}] = 3333 (m), 2925 (w), 1668 (vs), 1418 (s), 1343 (w), 1199 (m), 1101 (w) 745 (vs), 463 (w).

MS: MS (ESI, 60 eV): m/z (%) = 387.18 [M+]

Optical rotation: $[\alpha]_D^{20}$ = -46 (*c* 1.0, MeOH)

The analytical data are consistent with the literature.[1]

Double C3-methylated LL-cWW 5

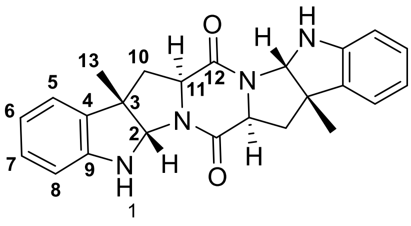

^{1}H NMR (600 MHz, $CDCl_3$) δ 7.05 (d, $^{3}J_{8,7}$ = 7.4 Hz, 1H, 8-H), 7.04 – 7.00 (m, 1H, 7-H), 6.74 (dd, $^{3}J_{6,5}$ = 7.4 Hz, $^{3}J_{6,7}$ = 7.4 Hz, 1H, 6-H), 6.52 (d, $^{3}J_{5,6}$ = 7.8 Hz, 1H, 5-H), 5.19 (s, 1H, 2-H), 5.04 (s, 1H, NH), 3.96 (dd, $^{3}J_{11,10b}$ = 11.0 Hz, $^{3}J_{11,10b}$ = 6.1 Hz, 1H, 11-H), 2.69 (dd, $^{2}J_{10b,10a}$ = 12.9 Hz, $^{3}J_{10b,11}$ = 6.2 Hz, 1H, Hb-10), 2.27 (dd, $^{2}J_{10a,10b}$ = 12.9 Hz, $^{3}J_{10a,11}$ = 11.3 Hz, 1H, Ha-10), 1.44 (s, 3H, 13-H).

^{13}C NMR (151 MHz, $CDCl_3$) δ 166.75 (C-12), 148.40 (C-9), 132.22 (C-4), 128.79 (C-7), 122.70 (C-8), 119.70 (C-6), 109.60 (H-5), 81.23 (C-2), 60.63 (C-11), 52.06 (C-3), 39.99 (C-10), 24.24 (C-13).

IR: IR (ATR): ṽ [cm^{-1}] = 3373 (m), 2960 (w), 1660 (vs), 1484 (m), 1424 (s), 1241 (w), 1165 (w), 1057 (w), 744 (s).

MS: MS (ESI, 60 eV): m/z (%) = 401.20 [M+]

Optical rotation: $[\alpha]_D^{20}$ = -704 (*c* 0.1, MeOH)

The analytical data are consistent with the literature.[1]

Figures and tables

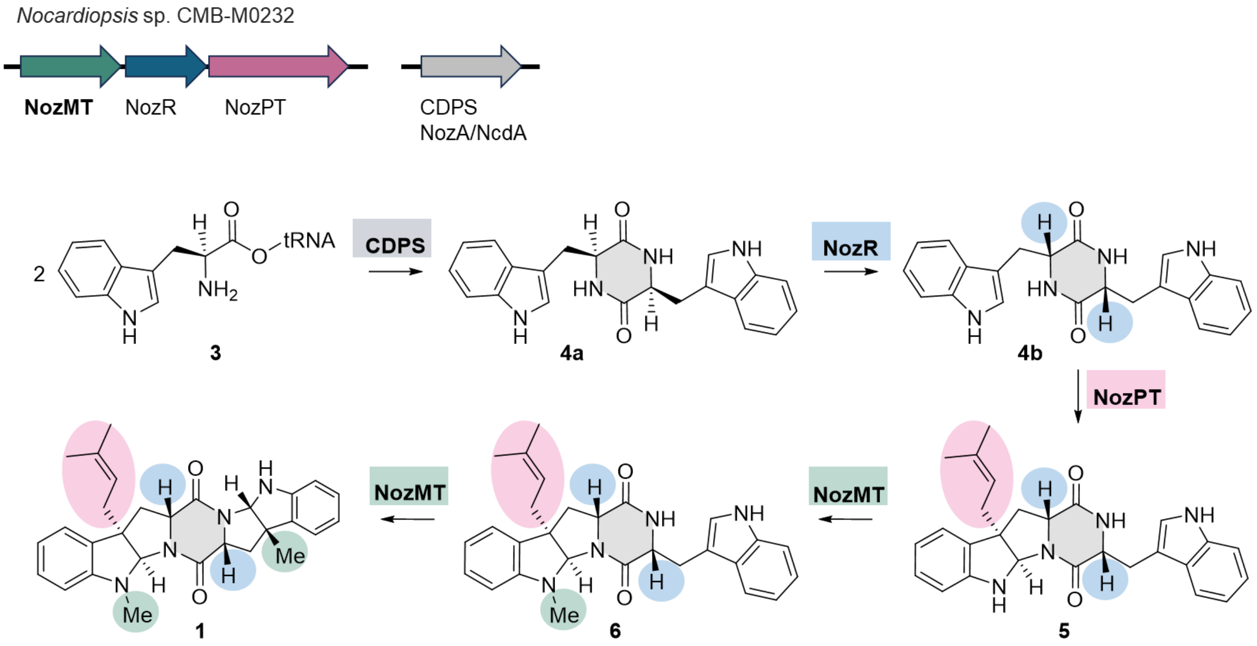

Figure S1: Gene cluster and biosynthesis pathway of nocardioazine B. The methyltransferase NozMT is highlighted in green, the isomerase NozR in brown, the prenyltransferase NozPT in pink and the CDPS NozA and NcdA in grey.

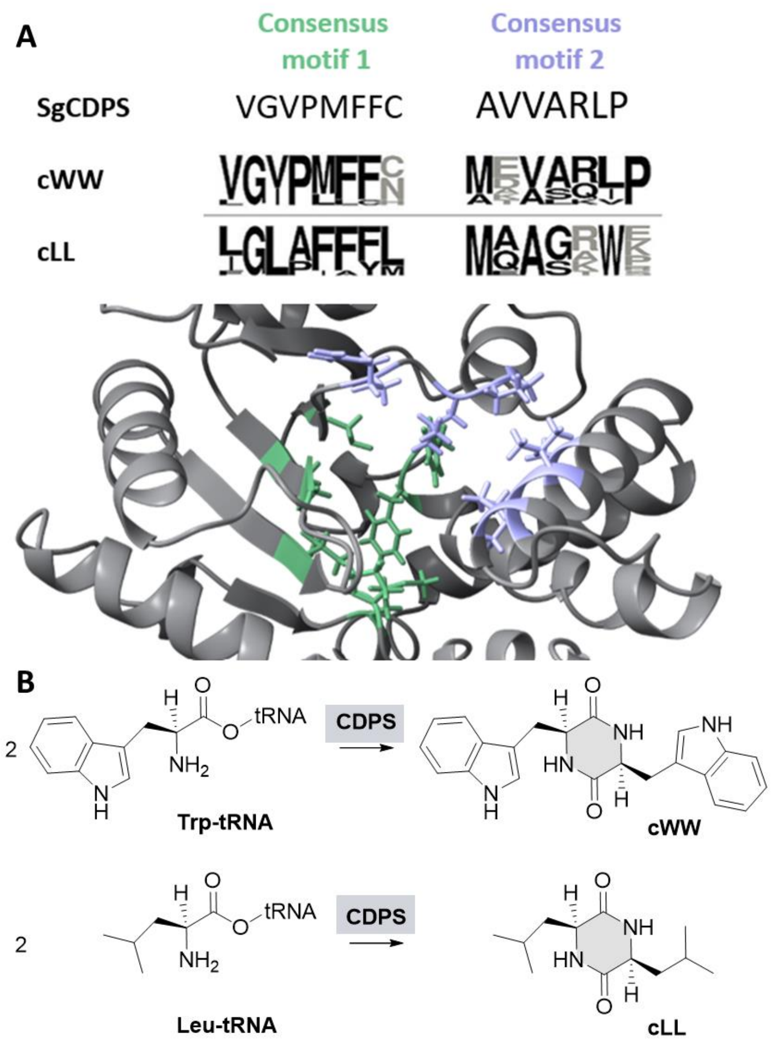

Figure S2: A: Analysis of the signature motifs of SgCDPS w.r.t. characterized CDPSs synthesising cWW or cLL. Consensus motif 1 (residues 33, 35, 65, 67, 119, 185, 186, 200; AlbC numbering) is marked in green; consensus motif 2 (residues 152, 155, 156, 159, 204, 206, 207; AlbC numbering) in violet. B: Reaction catalysed by a CDPS: The diketopiperazine motif is formed by condensing two tRNA bound amino acids (Leu or Trp).

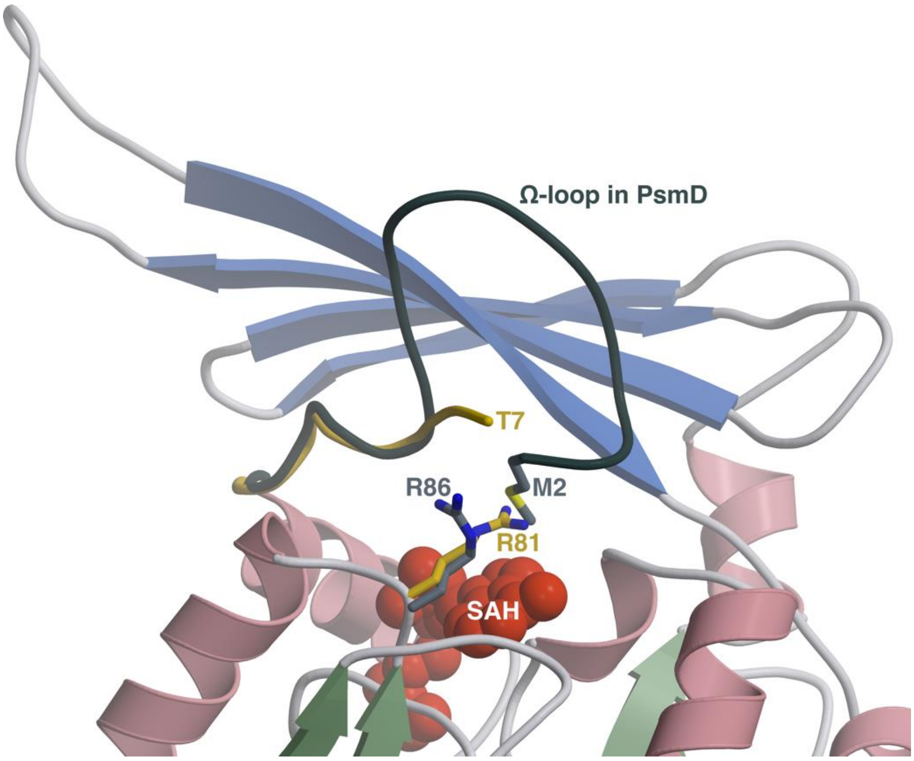

Figure S3: SgMT and PsmD shield the cofactor in different ways. The SgMT structure (PDB 9GDJ, chain A) is shown with its lid region and the extended R81 side chain in gold, and the cofactor SAH (spheres) in red. In PsmD (PDB 7ZKH, chain A; dark grey items) the lid segment forms an Ω-loop, allowing M2 to cooperate with R86 in sealing the catalytic cavity.

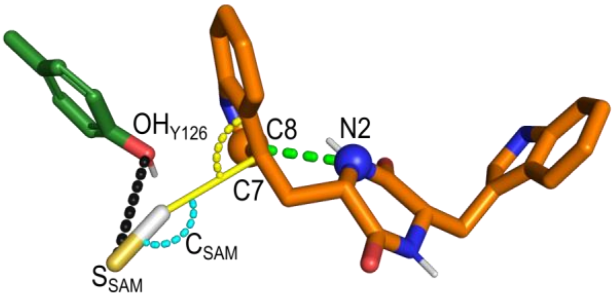

Figure S4: Geometric measurements considered for defining catalytically reactive LL-cWW binding poses. Black dashes: distance OHY126 - SSAM, cyan dashes: angle SSAM - CSAM-methyl - C7cWW indole, yellow line: distance CSAM-methyl - C7cWW indole, yellow dashes: angle S_{SAM} - $C_{SAM\text{-}methyl}$ - $C7_{cWW\ indole}$, yellow line: distance $C_{SAM\text{-}methyl}$ - $C7_{cWW\ indole}$, yellow dashes: angle $C_{SAM\text{-}methyl}$ - $C7_{cWW\ indole}$ - $C1_{cWW\ indole}$, green dashes: distance $C8_{cWW\ indole}$ - $N2_{cWW\ DKP}$.

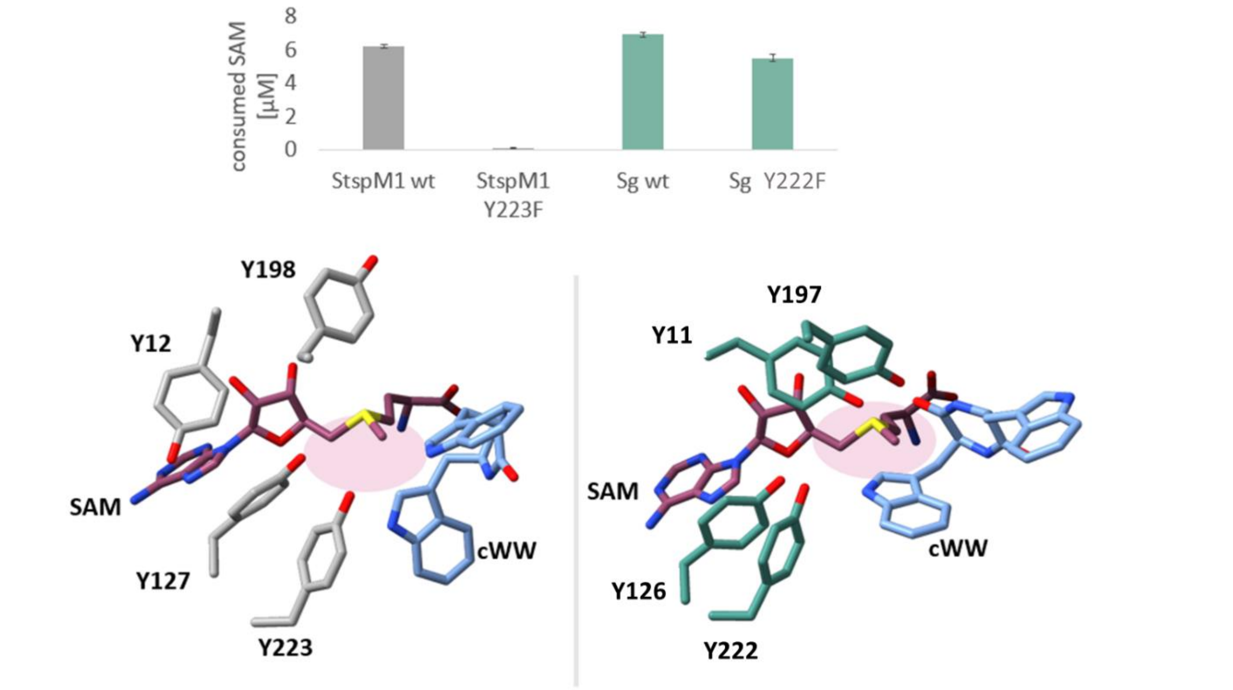

Figure S5: Comparison of tyrosine clusters in StspM1 (left) and SgMT (right). The tyrosines of StspM1 are shown in grey, the tyrosines of SgMT in green. The cofactor is shown in violet, the cWW in blue. The activity was measured with the glo Assay (Promega).

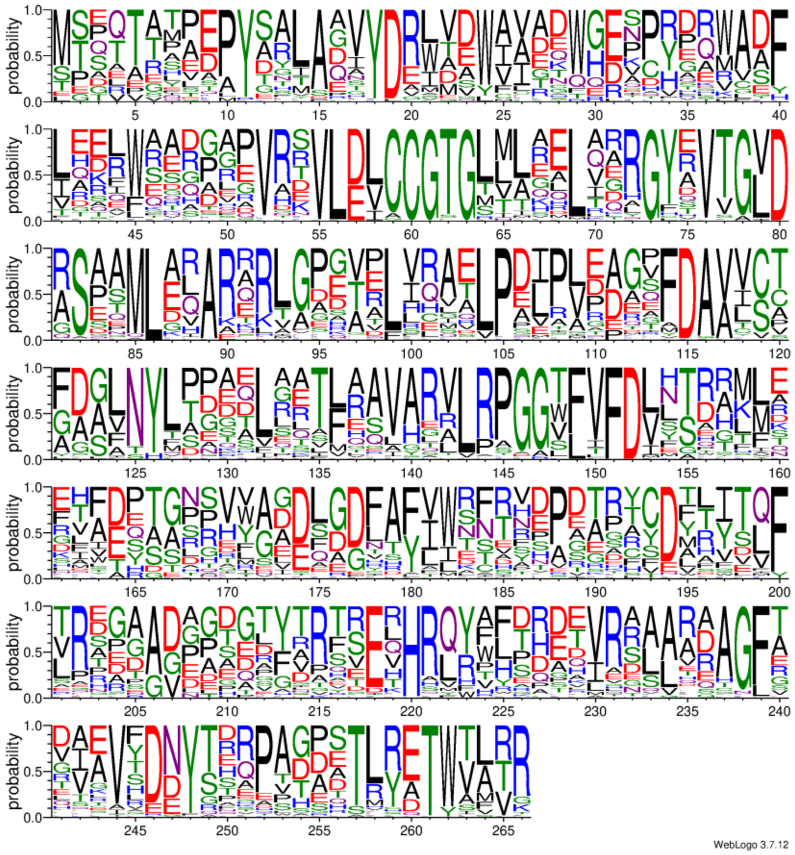

Figure S6: Residue conservation in SgMT homologs. The residue probability per position was derived using WebLogo 3 on a multiple sequence alignment of 60 sequences of SgMT homologs.

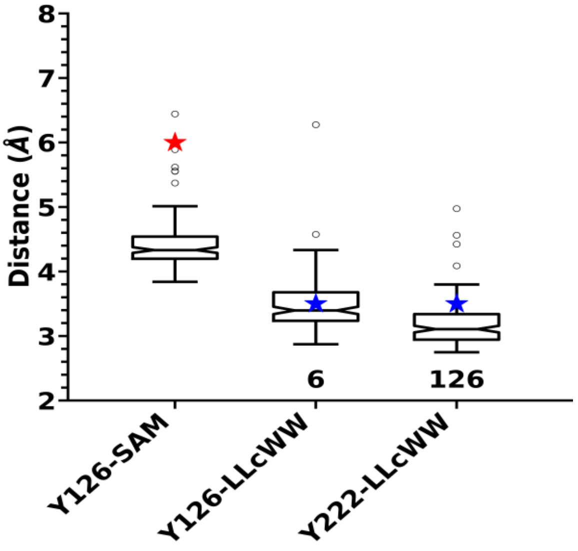

Figure S7: Relevant distances measured in MD simulations. Only snapshots for which the LL-cWW reactive carbon is within 4 Å of the SAM reactive methyl group were considered for the analysis. Y126-SAM: distance distribution between the Y126 hydroxyl group and the sulfur atom of the SAM cofactor. Red star: maximum Y126-SAM distance value measured in the crystal structures of five methyltransferases sharing a similar fold (PDB IDs 7ZKH, 1WZN, 1Y8C, 3D2L, 3BX0). Y126-LLcWW: distance distribution between the Y126 hydroxyl group and the nitrogen atom of the substrate reactive indole. Y222-LLcWW: distance distribution between the Y222 hydroxyl group and the nitrogen atom of the substrate reactive indole. Blue stars: maximum Y126-LLcWW / Y222-LLcWW distance value, below which a hydrogen bond can be formed. The number of hydrogen bonds formed between the corresponding Y126 and Y222 hydroxyl group and the reactive indole nitrogen atom of the substrate are indicated below the respective boxplots.

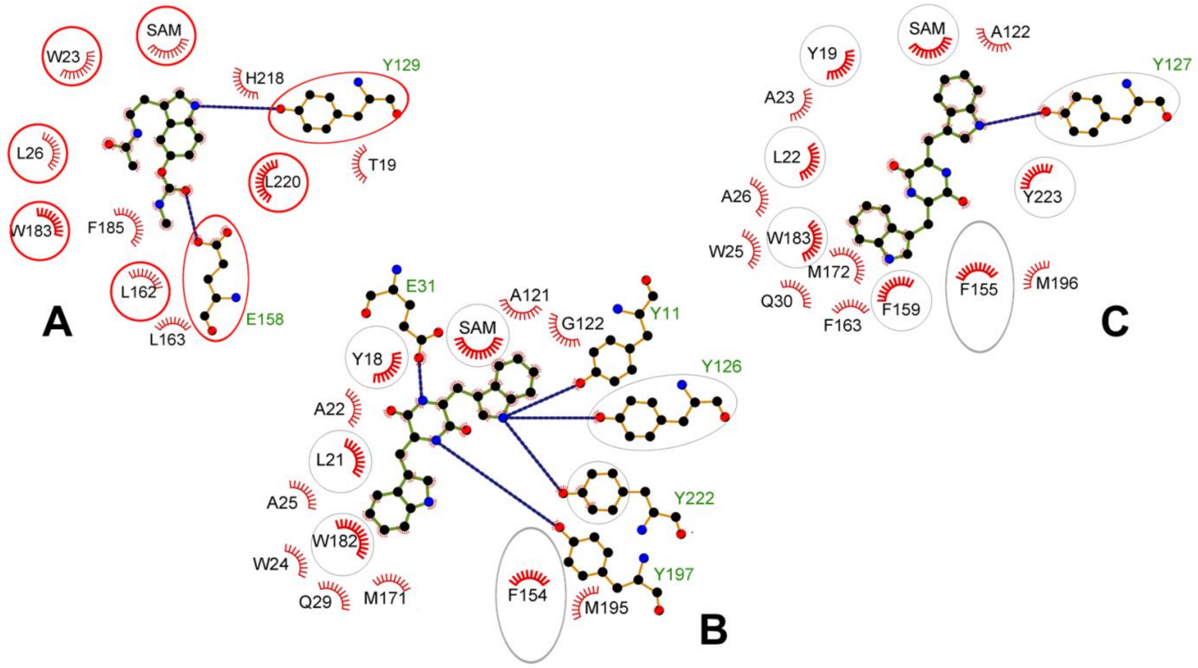

Figure S8: Substrate binding sites in PsmD (A), SgMT (B), and StspM (C). The 2D sketches were drawn using LigPlus, considering only binding residues within 4 Å from the bound substrate. Polar interactions are shown as a blue dashed line. Spatially equivalent positions in all three 3D structures are circled in red (PsmD) and grey (SgMT/StspM1)

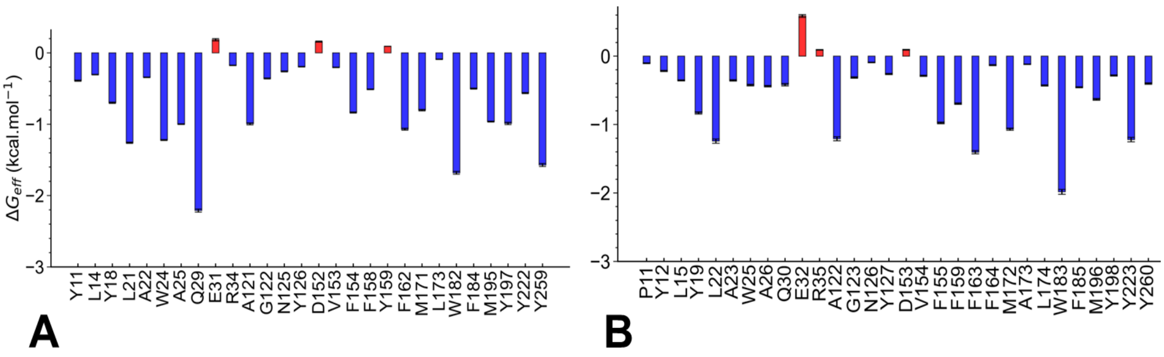

Figure S9: MM-GBSA effective free-energy per residue for SgMT (A) and StspM1 (B). Calculations for SgMT and StspM1 were done using LL-cWW as ligand for both enzymes. The averages and standard errors were calculated over eight simulation replicas (200 ns each). Blue and red bars indicate positive versus negative contributions to the binding effective energy, respectively.

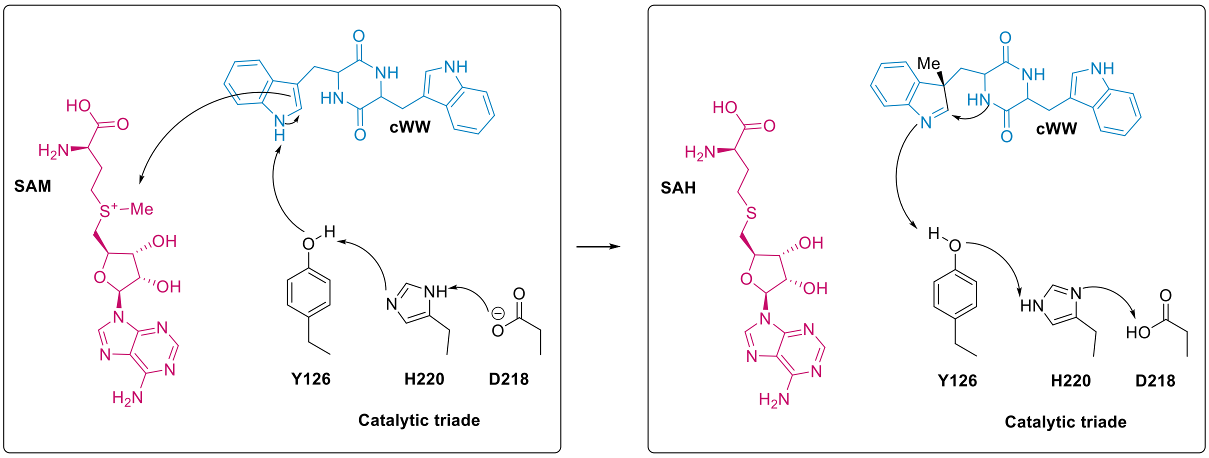

Figure S10: Mechanism of the indole C3-methylation catalyzed by SgMT. In the biocatalytic methylation reaction of the cWW substrate, the methyl group from the cofactor SAM is transferred to the C3 position of the indole ring in cWW, generating an electron sink on the indole nitrogen and forming a highly reactive iminium ion intermediate. Subsequently, the nucleophilic nitrogen in the diketopiperazine ring attacks the C2 position of the indole, resulting in the formation of the desired methylated product. SgMT features a catalytic triad comprising residues Y126, D218, and H220 supporting this mechanism.

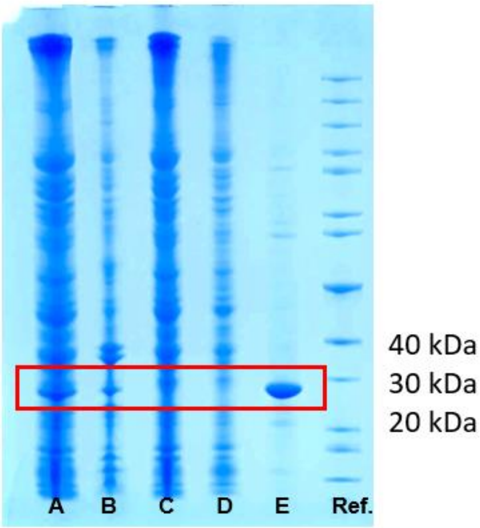

Figure S11: SDS gel of the purification of SgMT. 158.3 mg protein have been isolated from 4 g wet cells. As standard the Page Ruler unstained protein ladder (0.02 – 0.05 µg/µL) has been used. For the resuspended Lysate A, cell-pellet B and flow through C: 2 µL sample have been diluted in 10 µL Water. The washing fraction D (40 mM ImH) and the elution fraction E (250 mM ImH) have been diluted according to the given protein concentration. Ref. = Unstained Protein Standard, Broad Range (10-200 kDa), New England Biolabs.

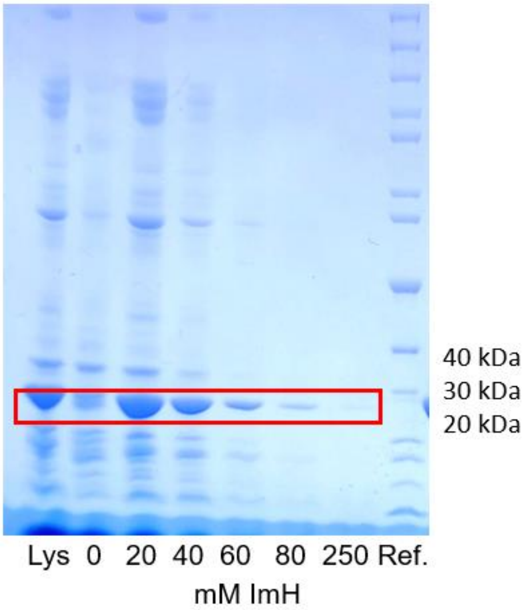

Figure S12: SDS-Gel of the thrombin His Tag cleavage. The lysate was applied to a Ni-NTA column and flushed with differnt concentrations of imidazol (0-250). Ref. = Unstained Protein Standard, Broad Range (10-200 kDa), New England Biolabs.

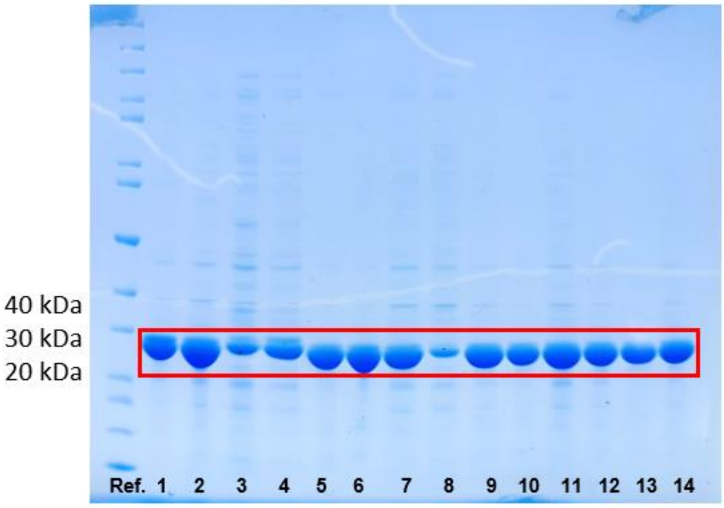

Figure S13: SDS gel of the alanine-scan mutants of SgMT. The purified proteins have been diluted according to the given protein concentration. 1: wt, 2: Y11A, 3: Y11F, 4: Y18A, 5 Y18F, 6: Y126A, 7: Y126F, 8: Y197A, 9: Y197F, 10: Y222A, 11: Y222F, 12: D218A, 13: D218E, 14: H220A. Ref. = Unstained Protein Standard, Broad Range (10-200 kDa), New England Biolabs.

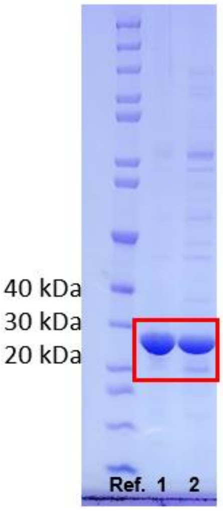

Figure S14: SDS gel of the StspM1 wt (1) and the Y223F (2) mutant. The purified proteins have been diluted according to the given protein concentration. Ref. = Unstained Protein Standard, Broad Range (10-200 kDa), New England Biolabs.

Table S2: X-ray data collection and refinement statistics. Values in parentheses refer to the highest-resolution shell.

Specimen	SgMT
PDB code	9GDJ
Data collection statistics	
Beamline	ESRF ID23-1
Detector	EIGER2 CdTe 16M
Wavelength [Å]	0.8856
Space group	$P\ 4_1\ 2_1\ 2$
Unit cell parameters	
a, b, c [Å]	123.9, 123.9, 126.4
α, β, γ [°]	90, 90, 90
Resolution range [Å]	37.43–1.47 (1.51–1.47)
No. reflections	166,186 (12,132)
Completeness [%]	100 (100)

Specimen<br>PDB code	SgMT<br>9GDJ
Multiplicity	13.2 (13.2)
Mean I/σ(I)	11.7 (0.8)
$CC_{1/2}$ [%]	99.8 (34.7)
Refinement statistics	
No. of reflections used	166,176
R_{work}	0.147
R_{free}	0.175
RMS deviations from ideal values	
Bonds [Å]	0.005
Angles [°]	0.740
Mean B [Å^2] (no. of atoms)	
Protein	30.4 (6816)
Ligands	54.0 (316)
Water	41.6 (815)
Ramachandran statistics [%]	
Favoured	98.7
Allowed	1.3
Outliers	0.0
Unusual rotamers [%]	0.9

Table S3: Temperature screen of SgMT: the activity of the methyltransferase SgMT was determined via the MTase Glo assay (Promega), which detects the consumption of SAM.

Temp [°C]	Consumed SAM [µM]	Stabw
22	0.95	0.14
30	2.34	0.52
36	3.81	0.36
40	5.91	0.59
45	6.54	0.33
50	2.34	0.08

Table S4: pH screen of SgMT: the conversion of the substrate was measured via HPLC. A citrate-phosphate-buffer was used for a pH range between pH 5.5 and 8, a tris-buffer between pH 7.65 and 8.55.

Buffer	pH	Conversion [%]	SD [%]
**Citrate-phosphate-buffer**	8.0	73.2	5.9
	7.5	69.7	6.9
	7.0	63.9	5.5
	6.5	57.5	4.7
	6.0	25.1	8.2
	5.5	8.5	2.1
	5.0	11.4	7.2
	4.0	0.0	0.0
**Tris-buffer**	7.65	73.3	6.9
	8.25	74.8	5.2
	8.55	66.0	3.2

Table S5: Effect of expression tags an SgMT catalytic activity. The activity of the methyltransferase SgMT was determined via the MTase Glo assay (Promega), which detects the consumption of SAM.

	Consumed SAM [µM]	SD
**N-HisTag**	4.39	0.02
**C-HisTag**	7.18	0.15
**Tag removed**	7.48	0.03

Table S6: Determination of the kinetic parameters of SgMT. The activity of the methyltransferase SgMT was determined via the MTase Glo assay (Promega), which detects the consumption of SAM.

Substrate conc [µM]	v [µM/min]	SD
50.00	0.35	0.05
25.00	0.33	0.05
12.50	0.25	0.04
6.25	0.17	0.06
3.13	0.08	0.03
1.56	0.03	0.03
0.78	0.01	0.03
0.39	0.02	0.02
0.20	0.01	0.01
0.10	0.01	0.00

Table S7: Substrate scope of SgMT. The activity of the methyltransferase SgMT was determined via the MTase Glo assay (Promega), which detects the consumption of SAM.

Substrate	Consumed SAM [µM]	SD
**4a**	7.18	0.32
**4b**	1.90	0.10
**4c**	0.81	0.06
**6a**	0.26	0.02
**6b**	-	0.02
**6c**	-	0.04
**6d**	-	0.03
**7a**	1.05	0.05
**7b**	0.03	0.01
**7c**	0.33	0.00
**7d**	0.02	0.02
**8a**	-	0.05
**8b**	-	0.05
**8c**	-	0.01
**8d**	-	0.02
**9**	-	0.05
**10**	-	0.02
**11**	0.65	0.05
**12a**	0.92	0.21
**12b**	0.24	0.12
**13a**	5.91	0.17

**13b**	0.01	0.01
**5**	0.05	0.03

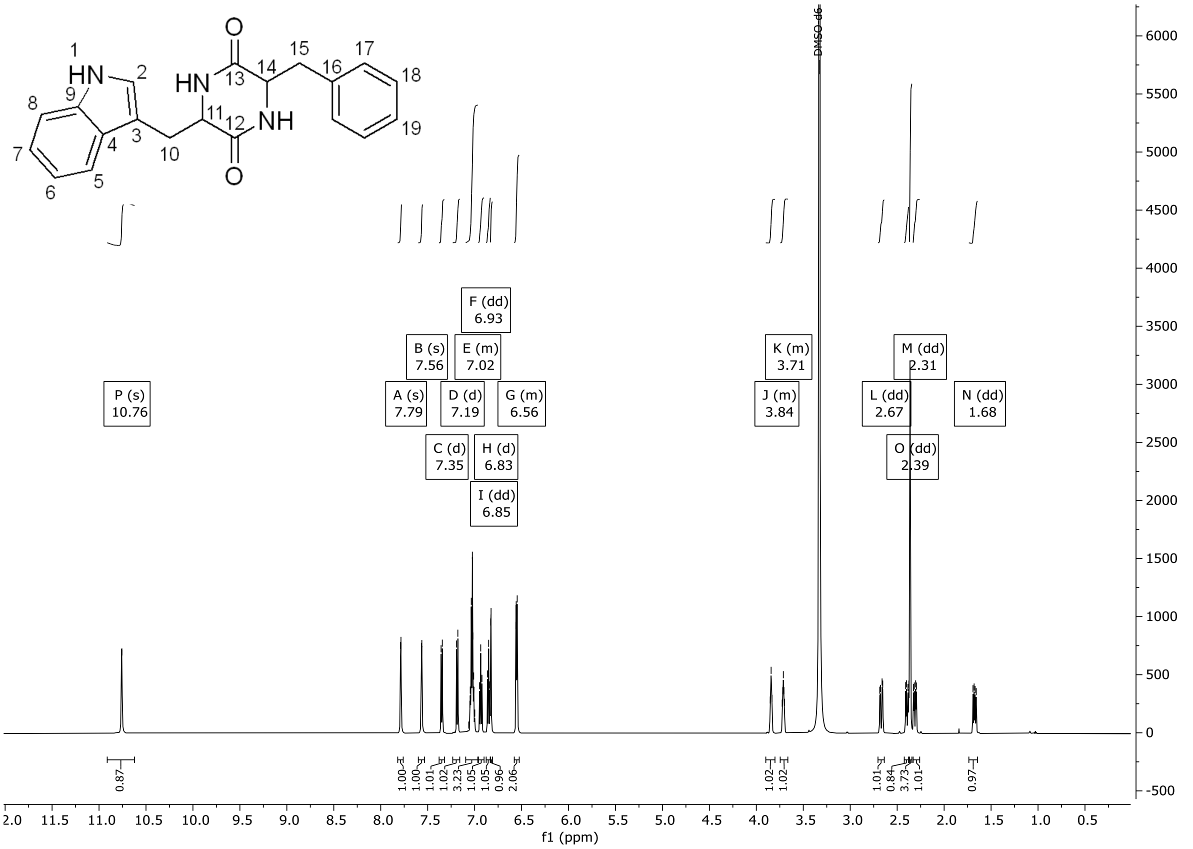

*Figure S15: ^{1}H-NMR spectrum of LL-cWF **7a** in DMSO (600 MHz).*

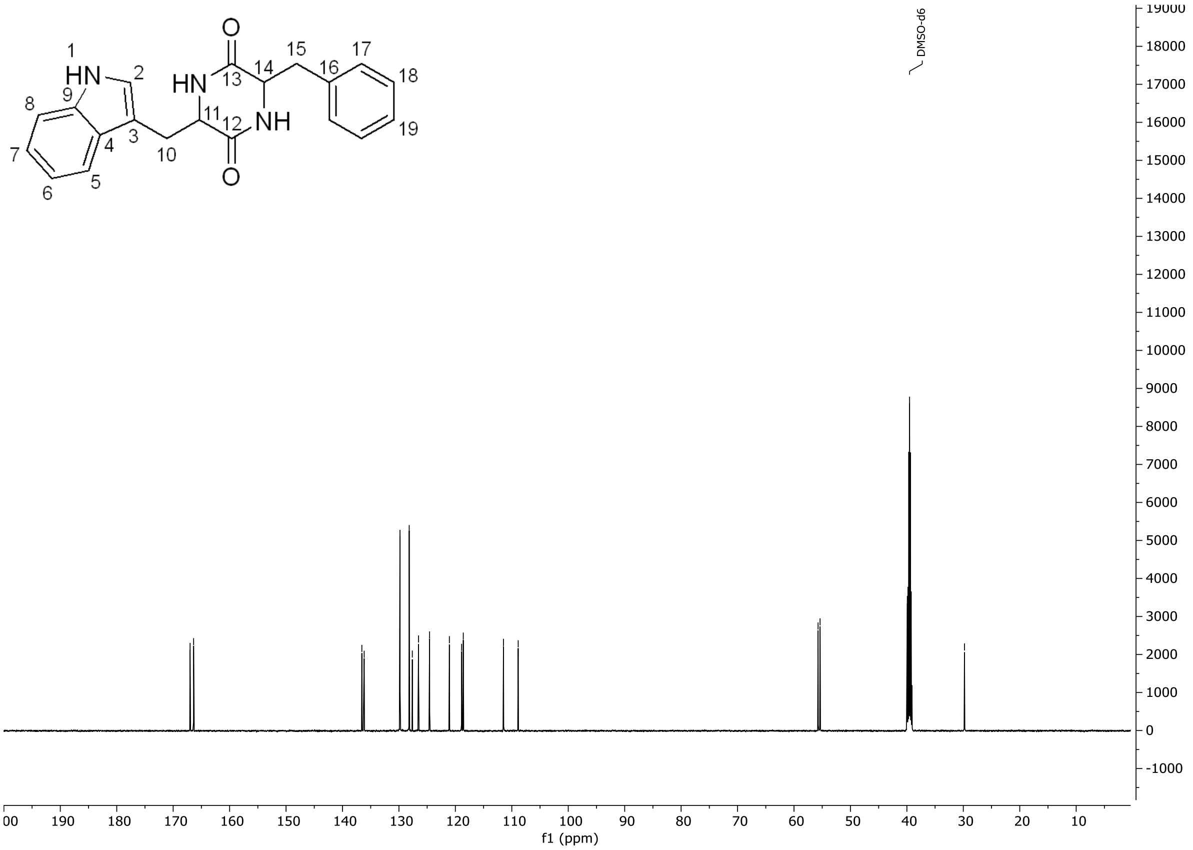

*Figure S16: ^{13}C-NMR spectrum of LL-cWF **7a** in DMSO (151 MHz).*

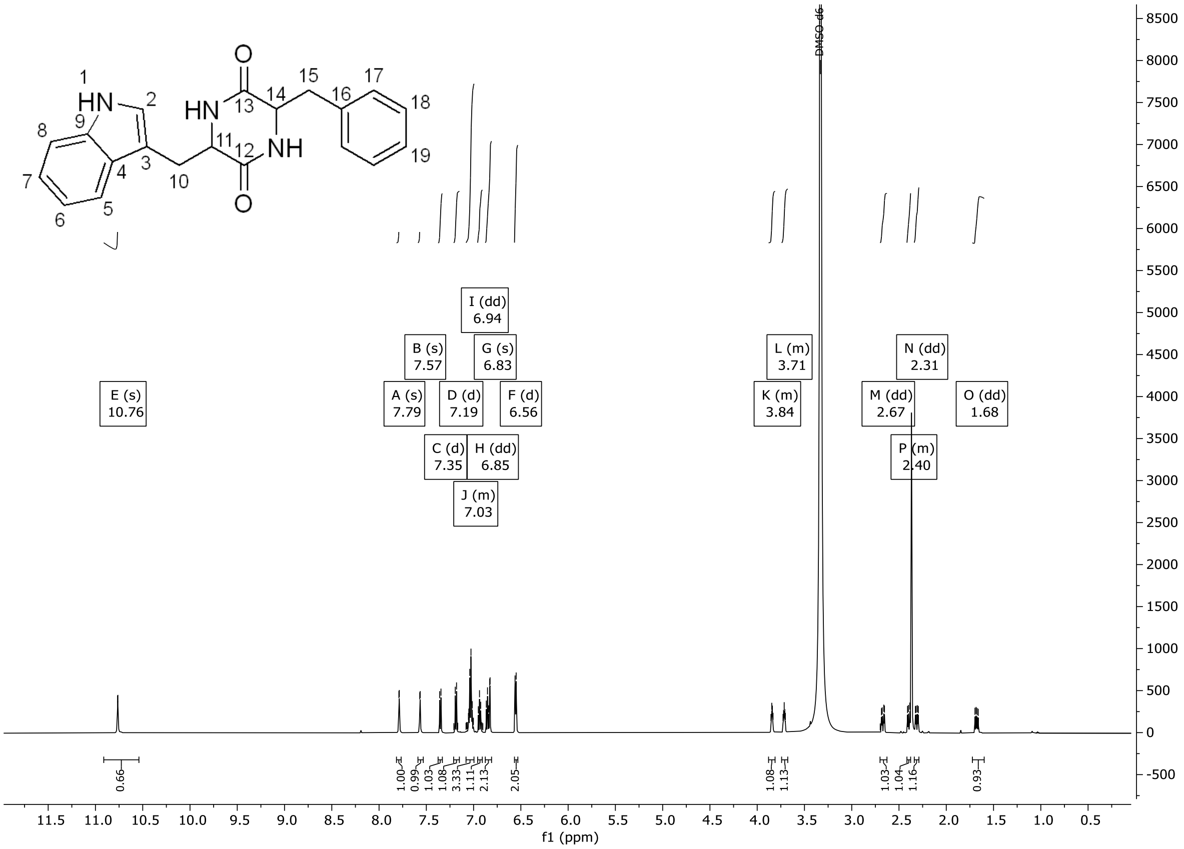

*Figure S17: ^{1}H-NMR spectrum of DD-cWF **7b** in DMSO (600 MHz).*

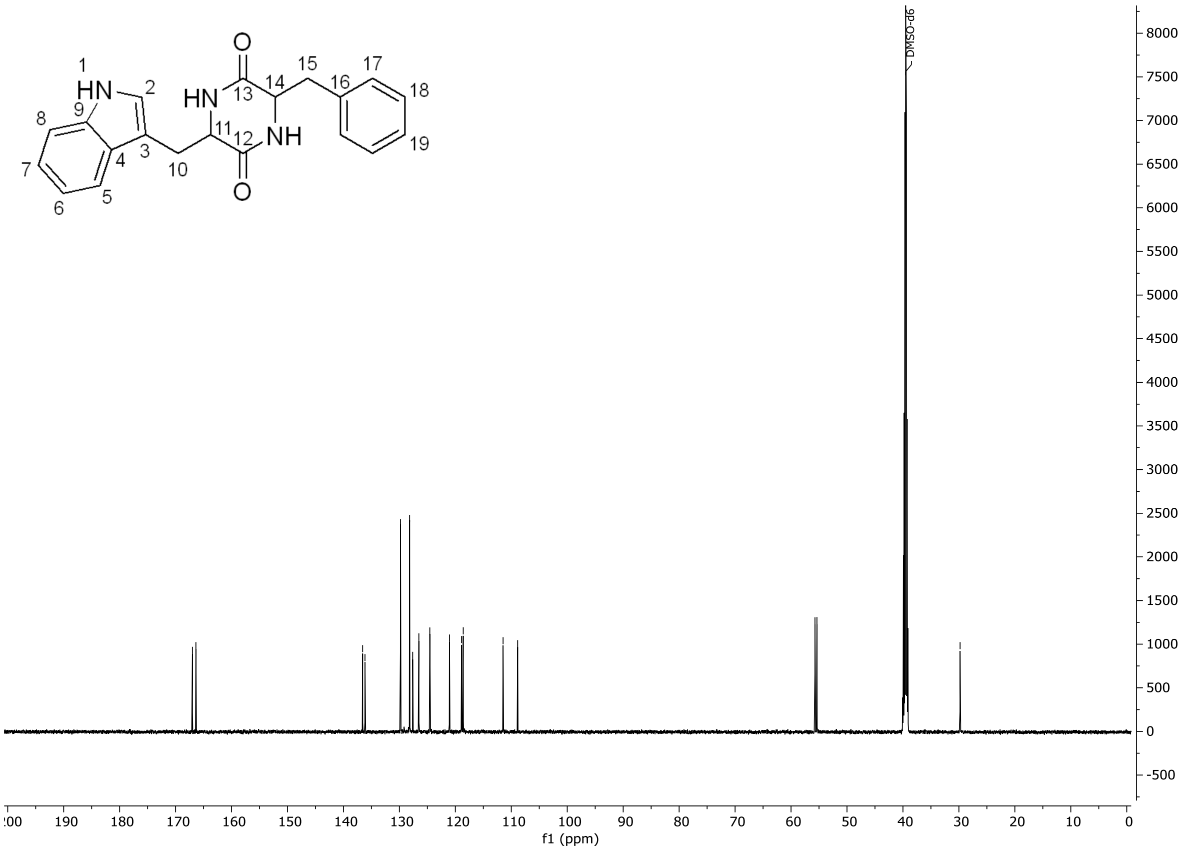

*Figure S18: ^{13}C-NMR spectrum of DD-cWF **7b** in DMSO (151 MHz).*

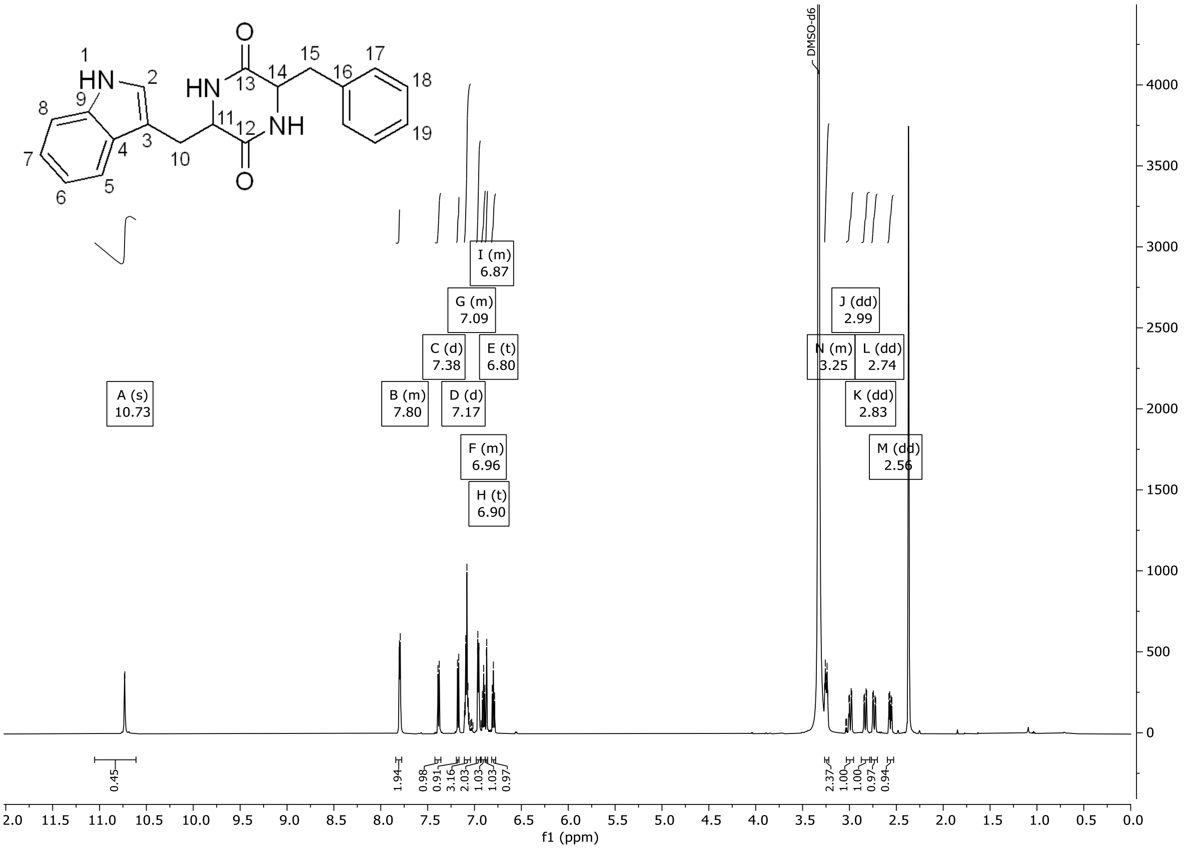

*Figure S19: ^{1}H-NMR spectrum of LD-cWF **7c** in DMSO (600 MHz).*

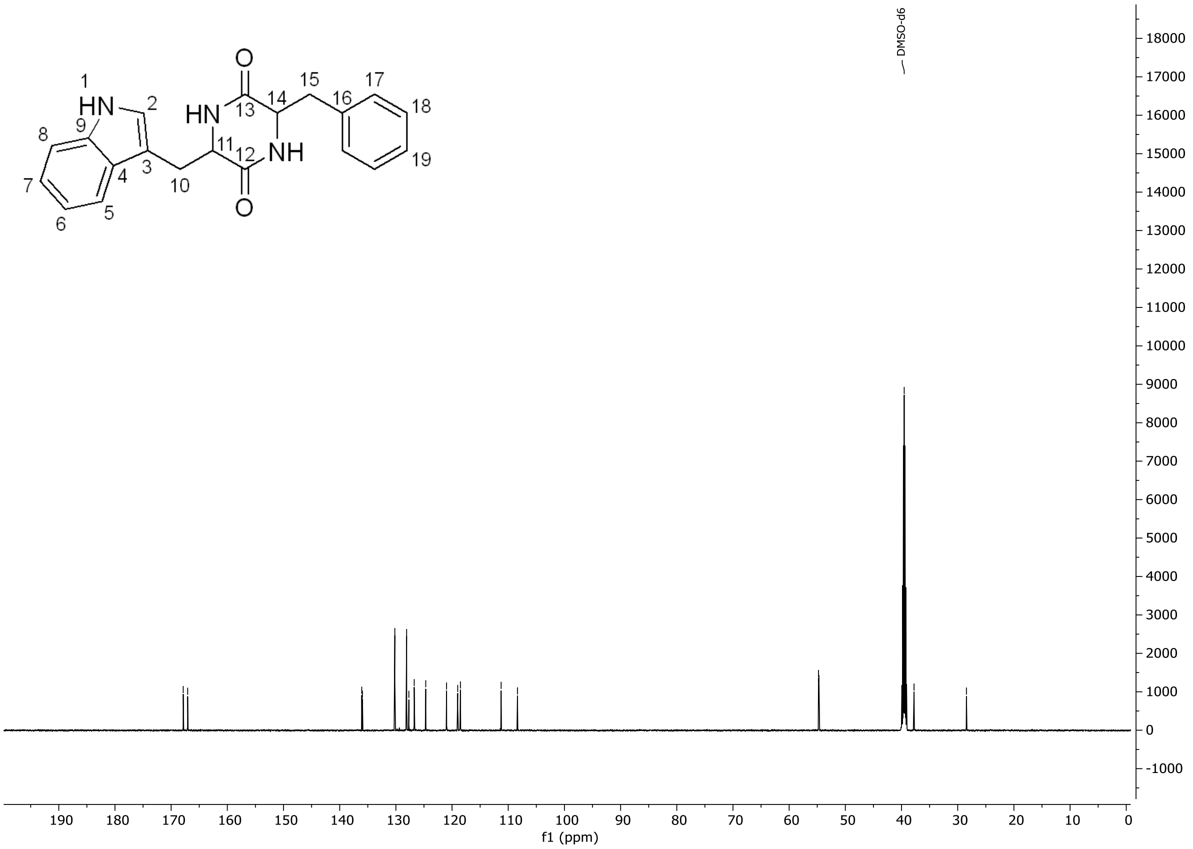

*Figure S20: ^{13}C-NMR spectrum of LD-cWF **7c** in DMSO (151 MHz).*

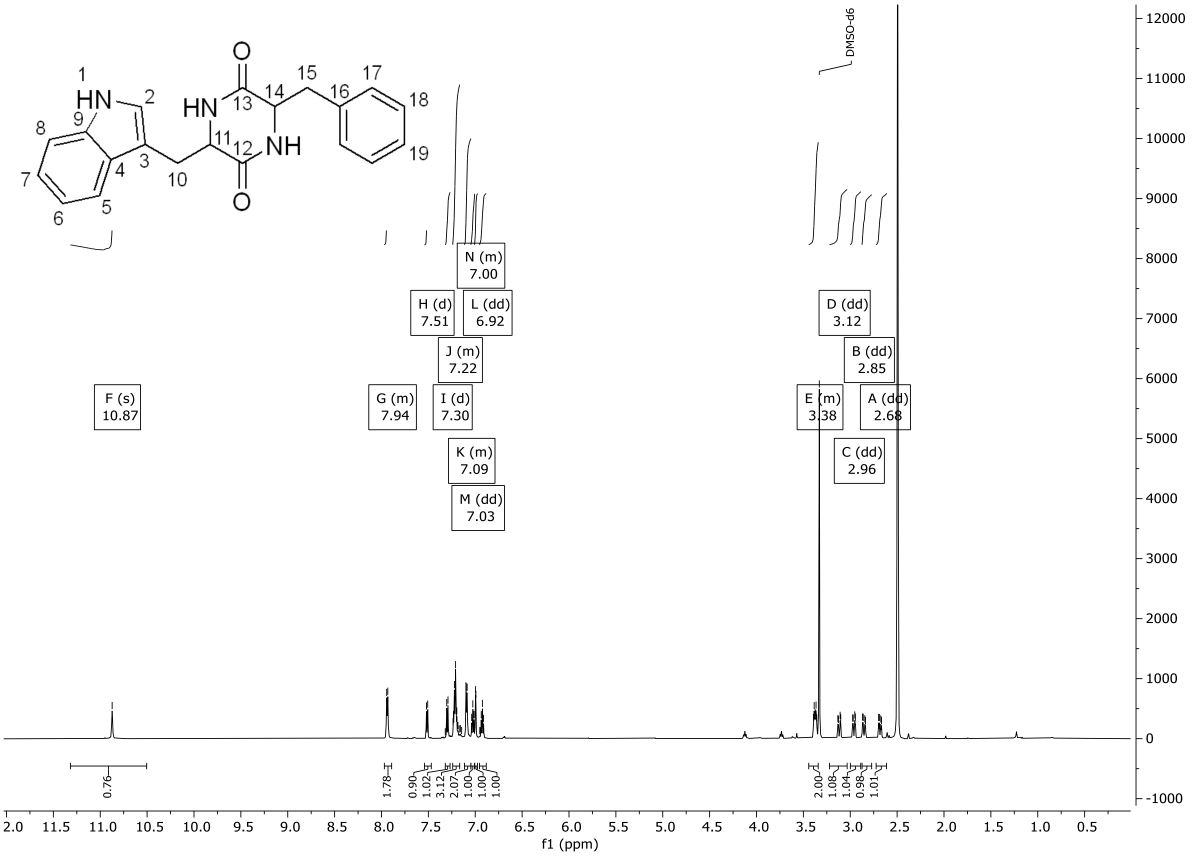

*Figure S21: ¹H-NMR spectrum of DL-cWF **7d** in DMSO (600 MHz).*

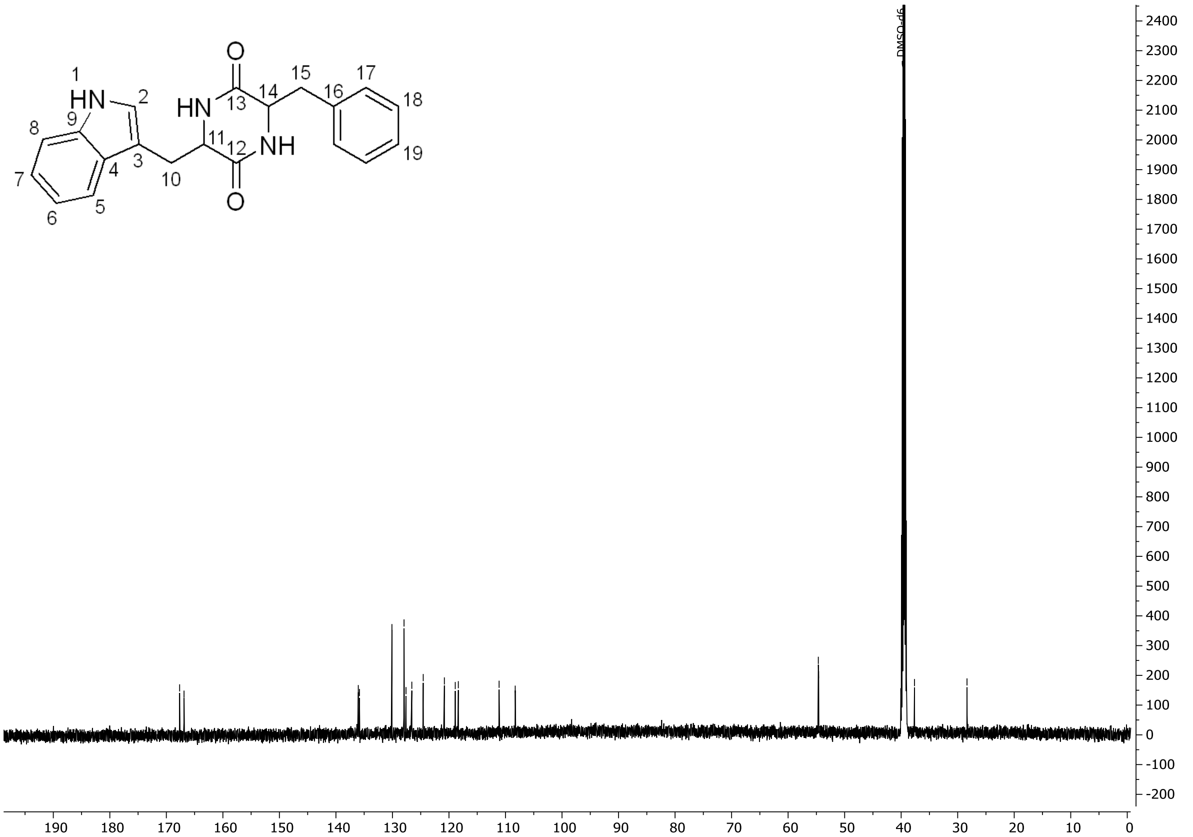

*Figure S22: ¹³C-NMR spectrum of DL-cWF **7d** in DMSO (151 MHz).*

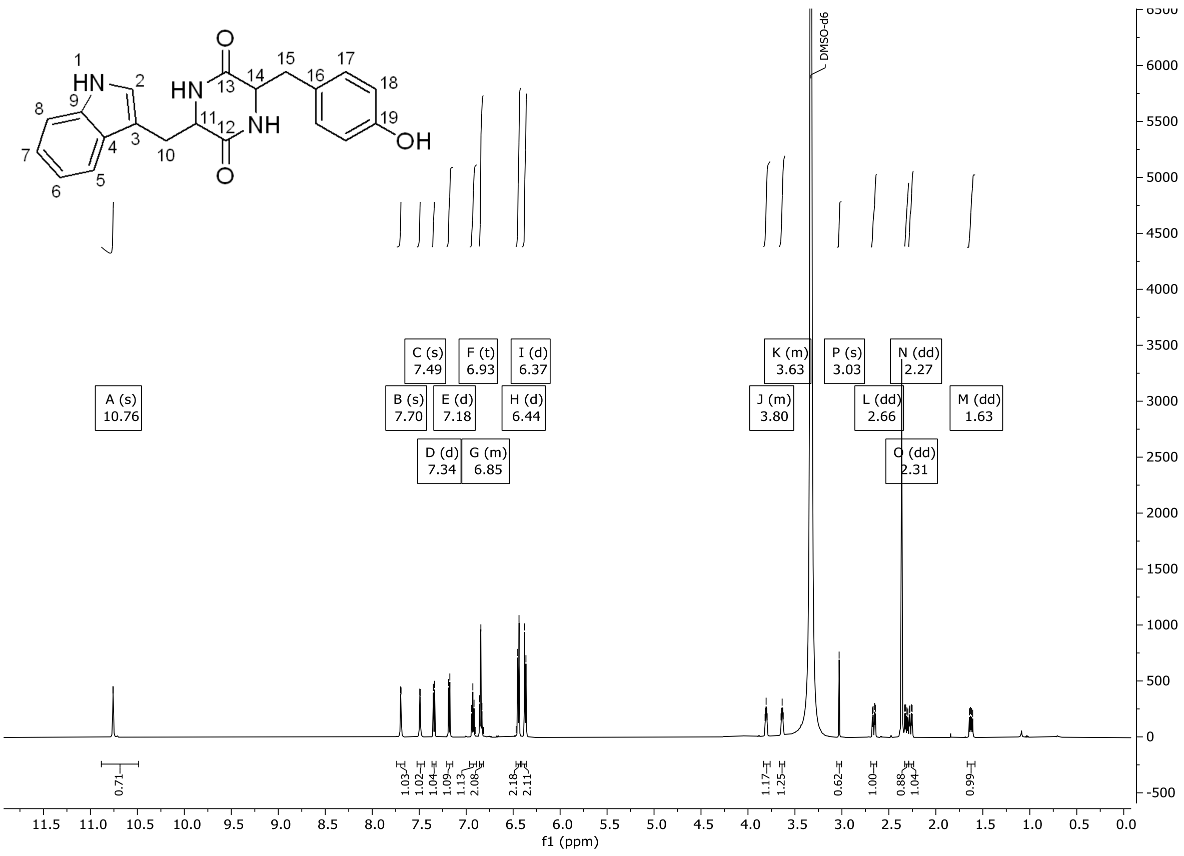

*Figure S23: ^{1}H-NMR spectrum of LL-cWY **6a** in DMSO (600 MHz).*

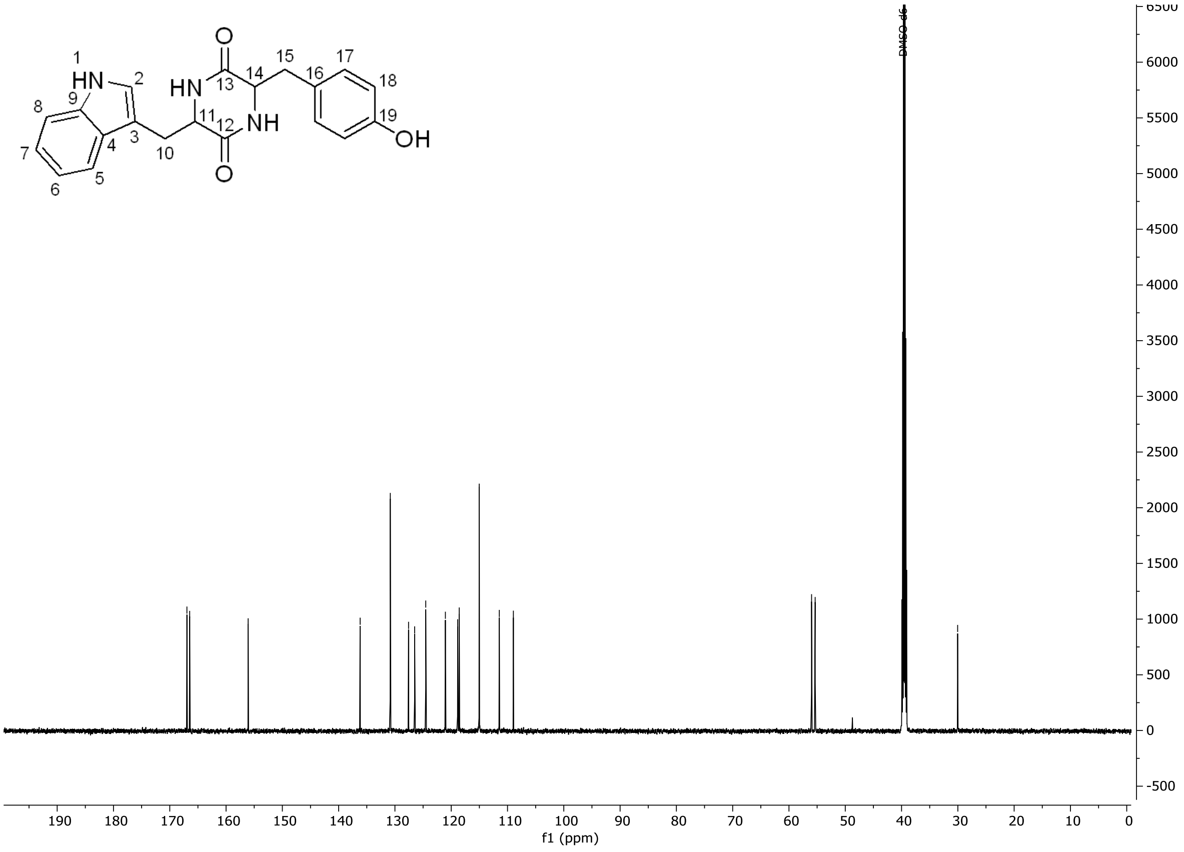

*Figure S24: ^{13}C-NMR spectrum of LL-cWY **6a** in DMSO (151 MHz).*

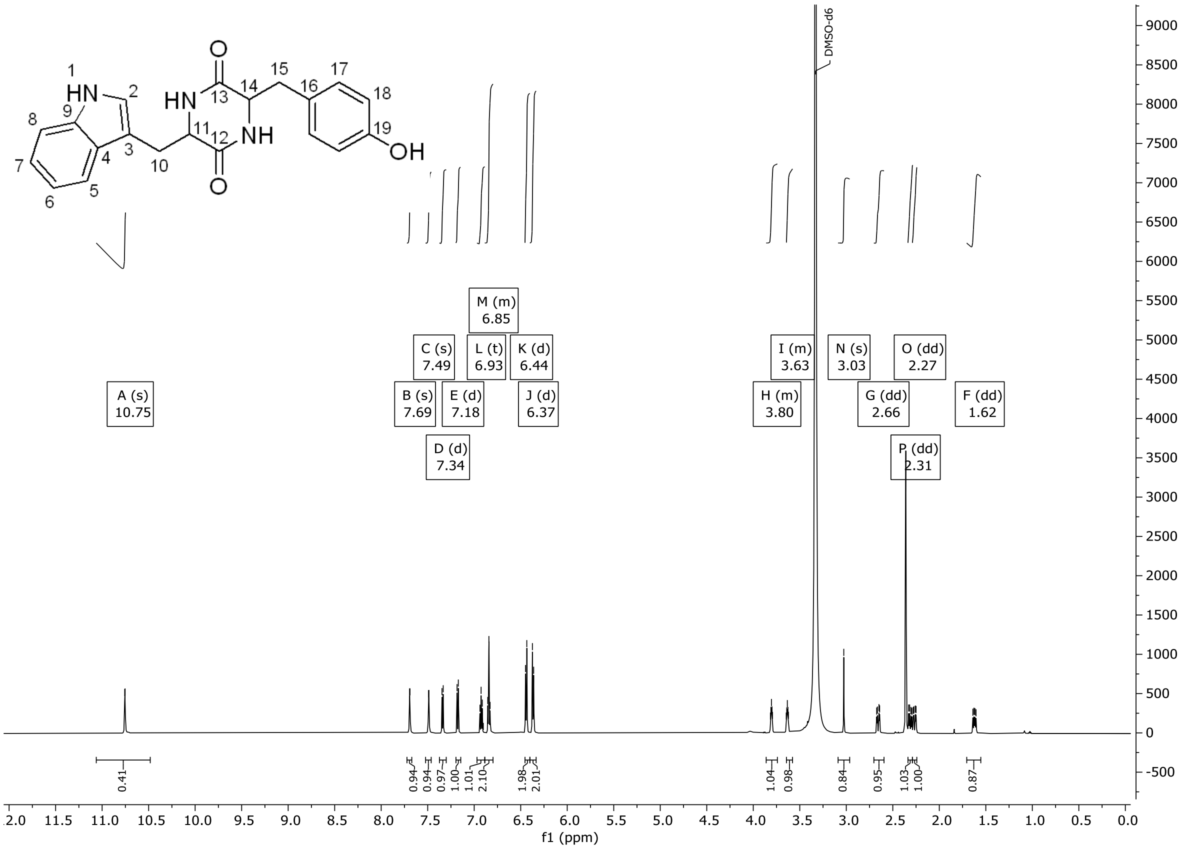

*Figure S25: ^{1}H-NMR spectrum of DD-cWY **6b** in DMSO (600 MHz).*

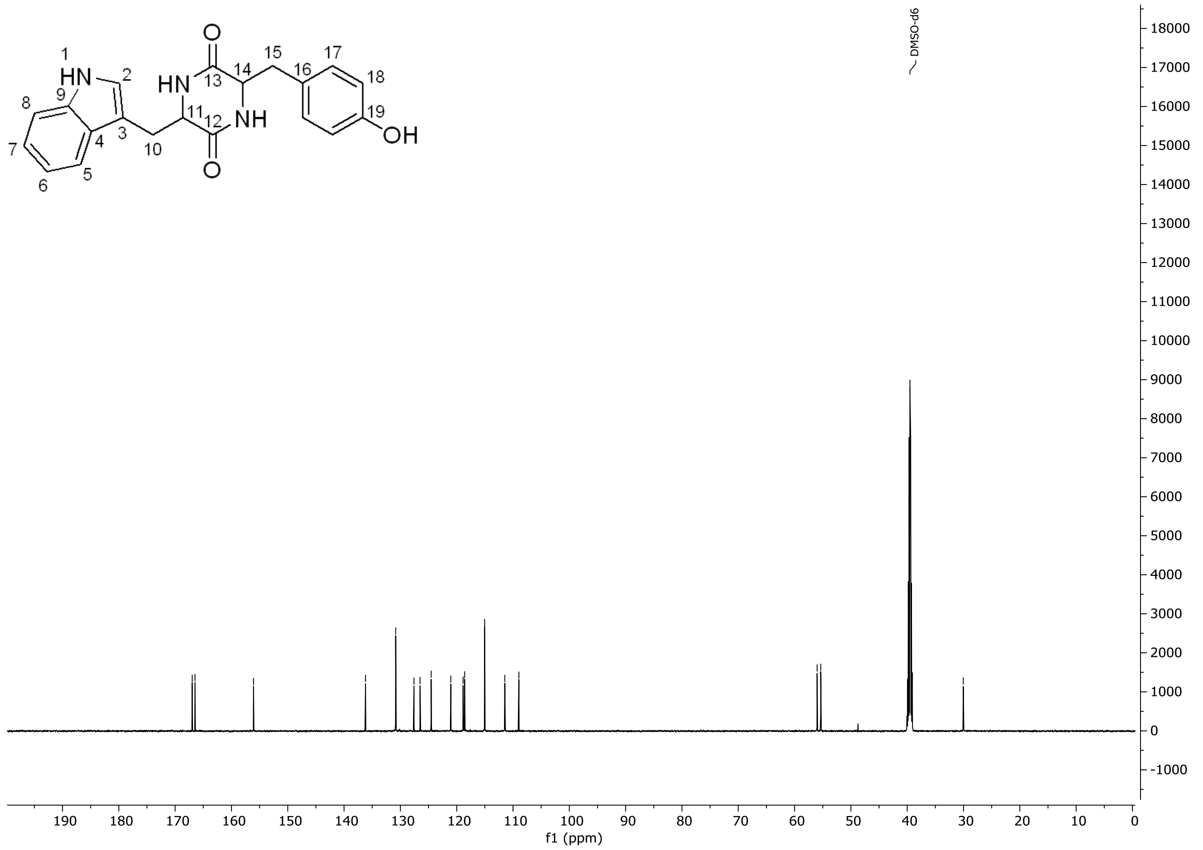

*Figure S26: ^{13}C-NMR spectrum of DD-cWY **6b** in DMSO (151 MHz).*

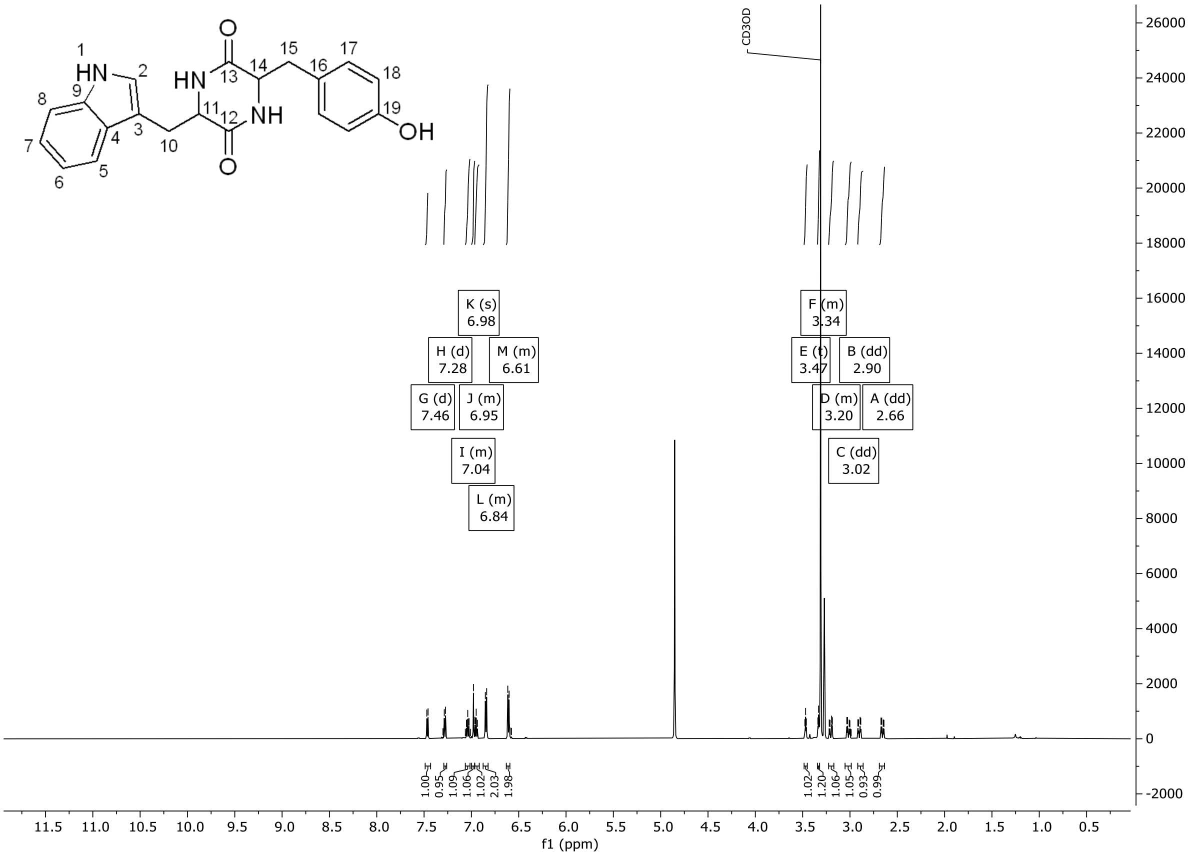

*Figure S27: ^{1}H-NMR spectrum of LD-cWY **6c** in MeOD (600 MHz).*

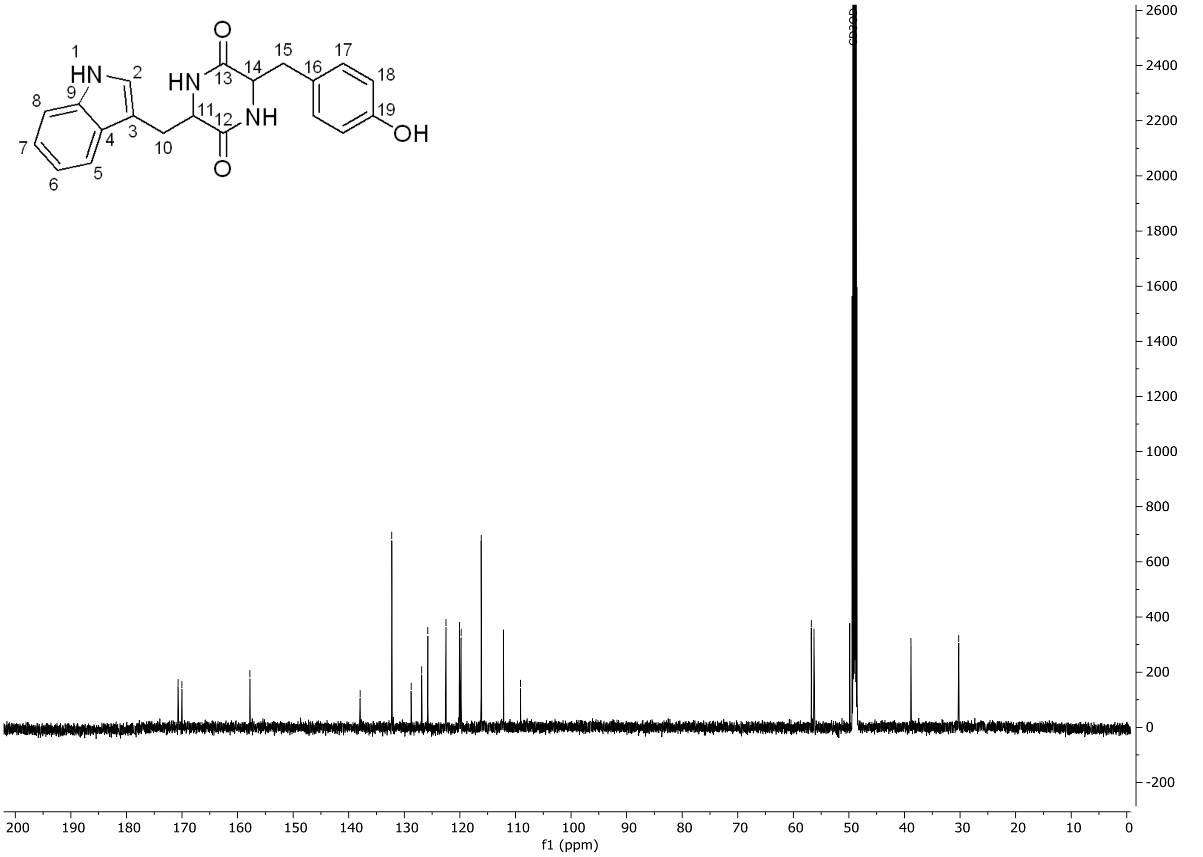

*Figure S28: ^{13}C-NMR spectrum of LD-cWY **6c** in DMSO (151 MHz).*

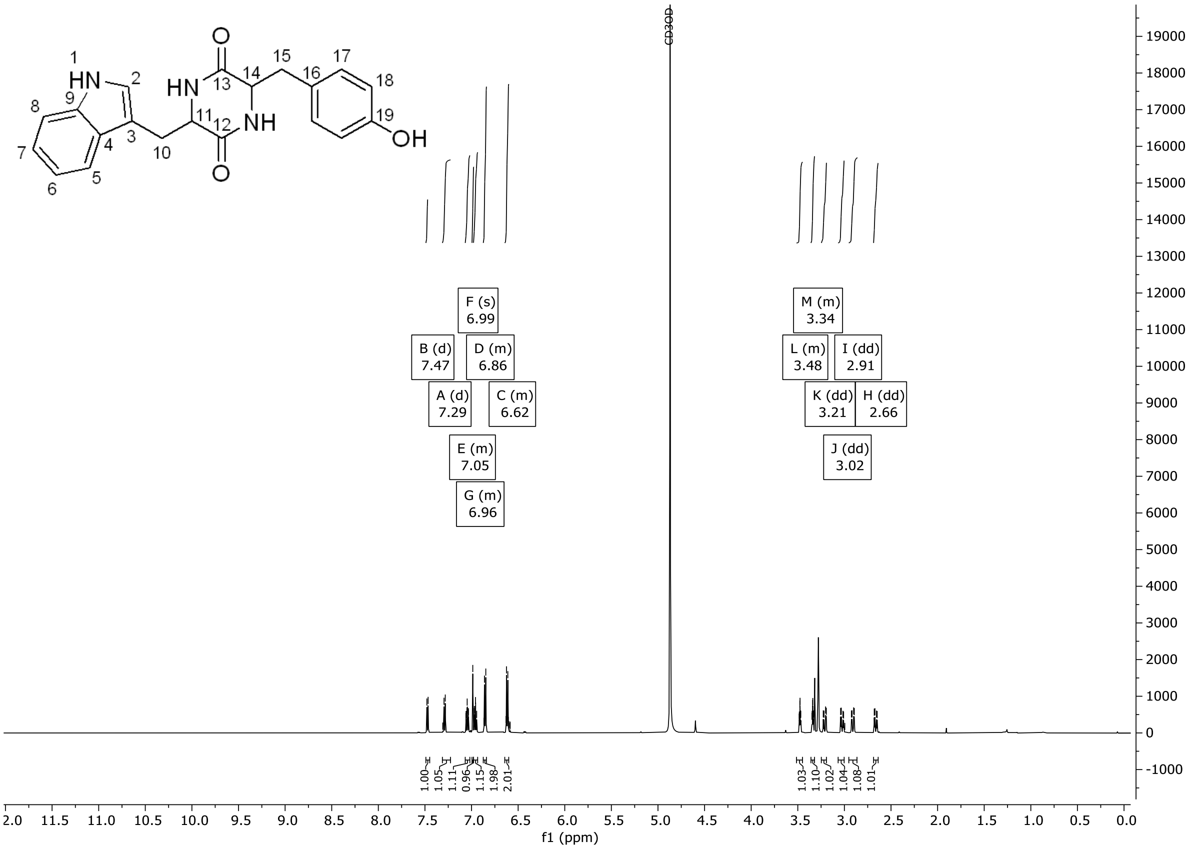

*Figure S29: ^{1}H-NMR spectrum of DL-cWY **6d** in MeOD (600 MHz).*

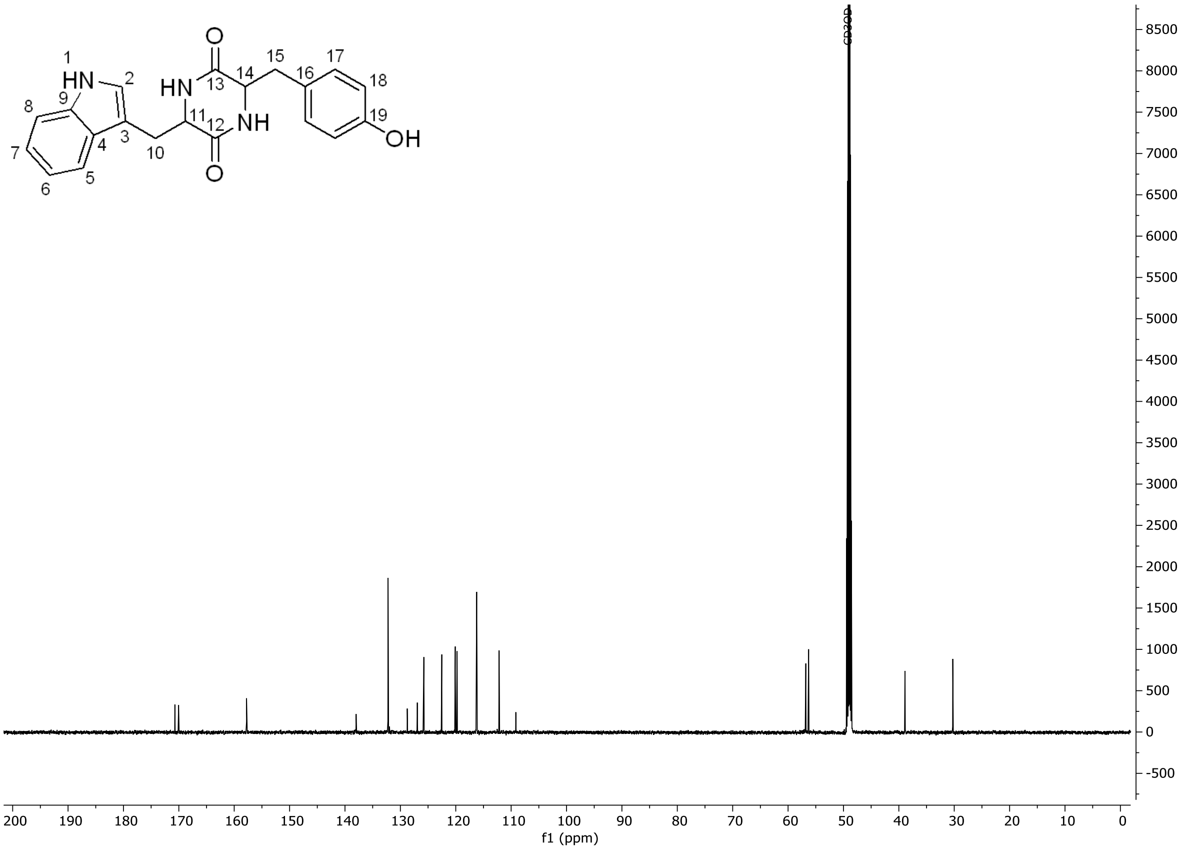

*Figure S30: ^{13}C-NMR spectrum of DL-cWY **6d** in DMSO (151 MHz).*

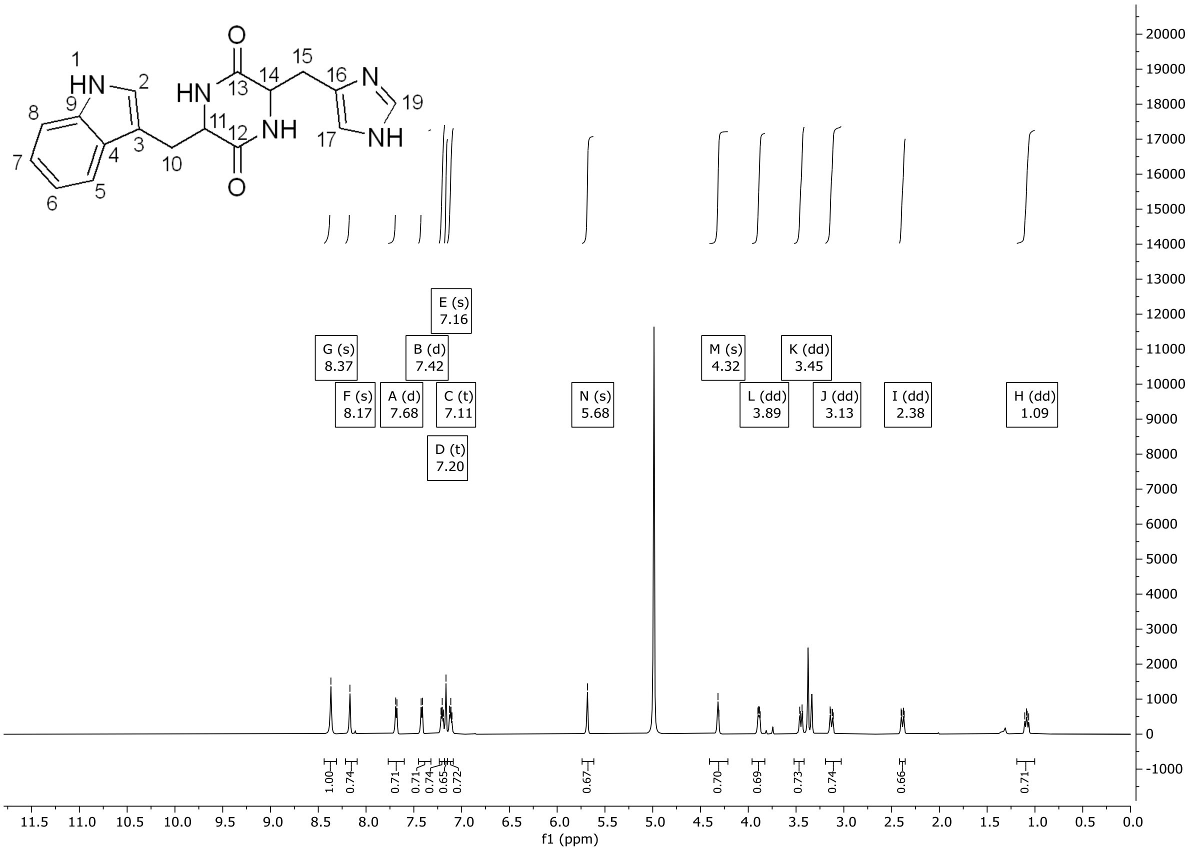

*Figure S31: ^{1}H-NMR spectrum of LL-cWH **8a** in MeOD (600 MHz).*

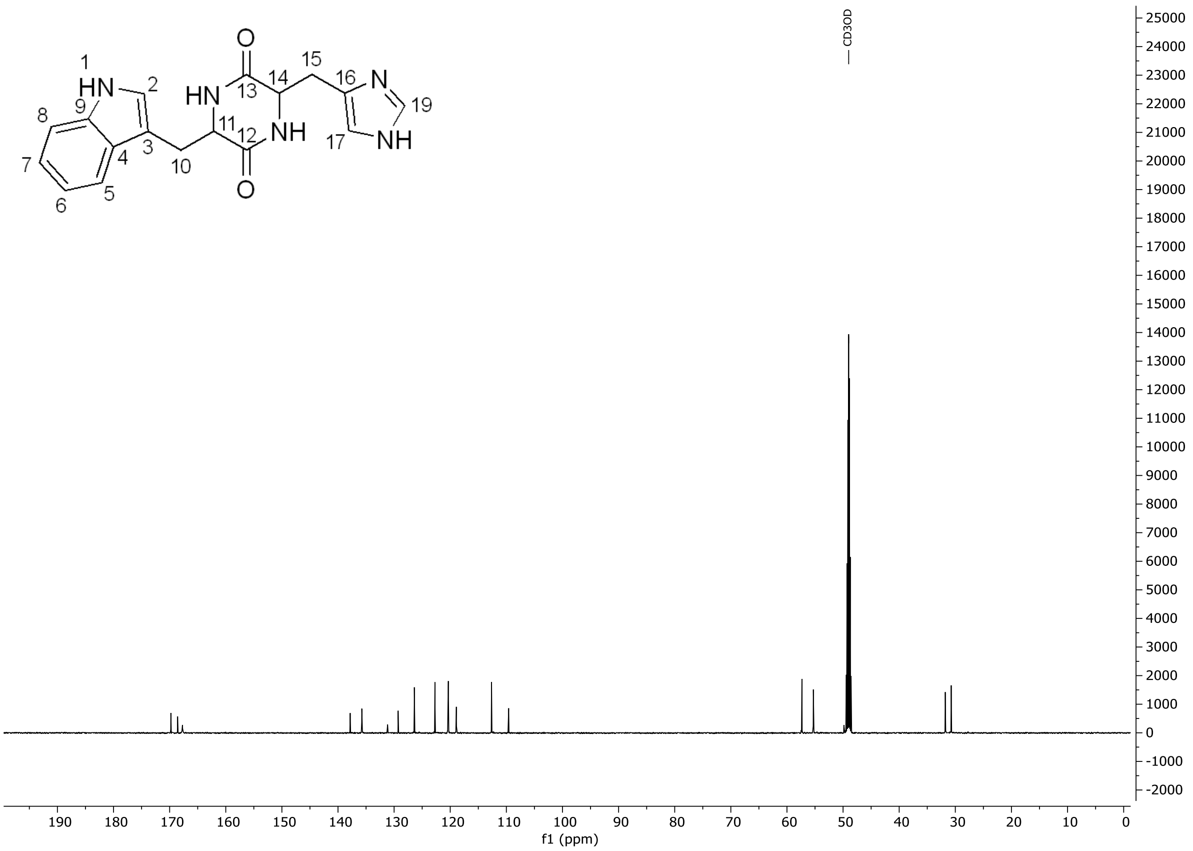

*Figure S32: ^{13}C-NMR spectrum of LL-cWH **8a** in MeOD (151 MHz).*

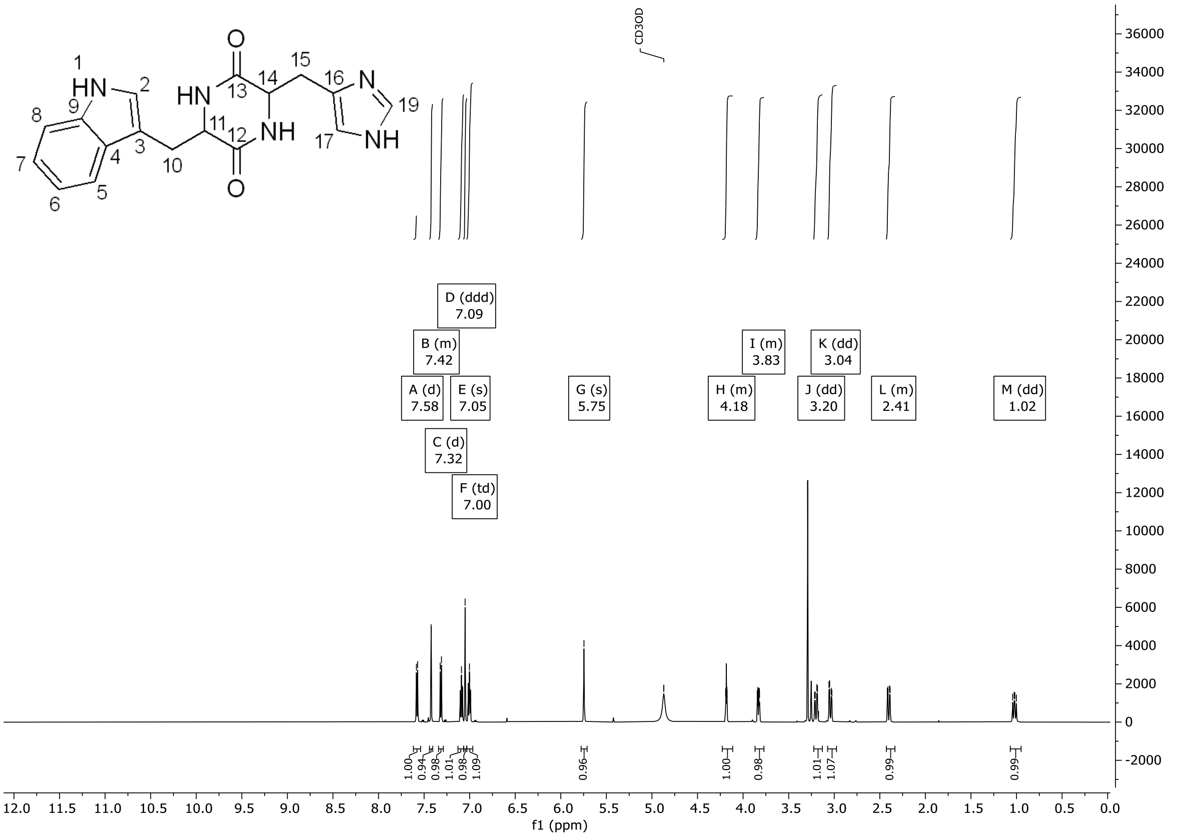

Figure S33: ^{1}H-NMR spectrum of DD-cWH ***8b*** *in MeOD (600 MHz).*

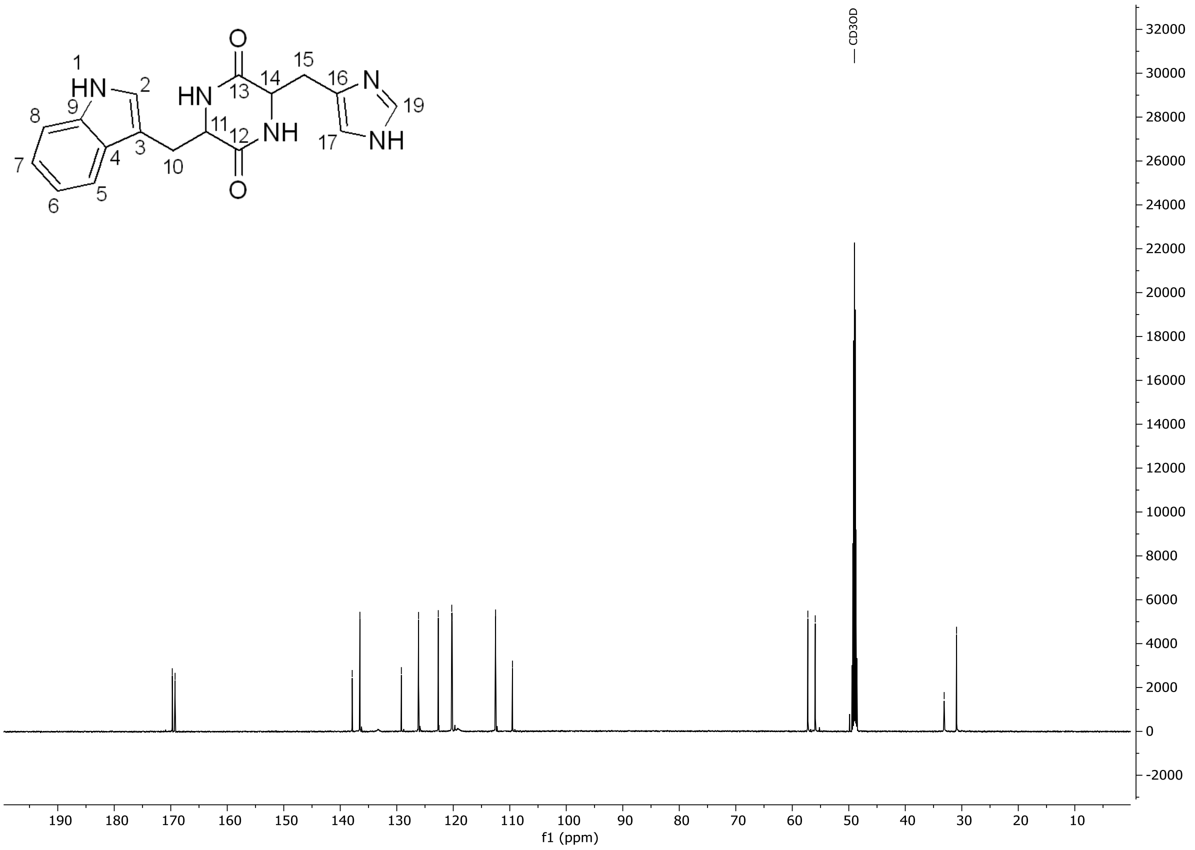

Figure S34: ^{13}C-NMR spectrum of DD-cWH ***8b*** *in MeOD (151 MHz).*

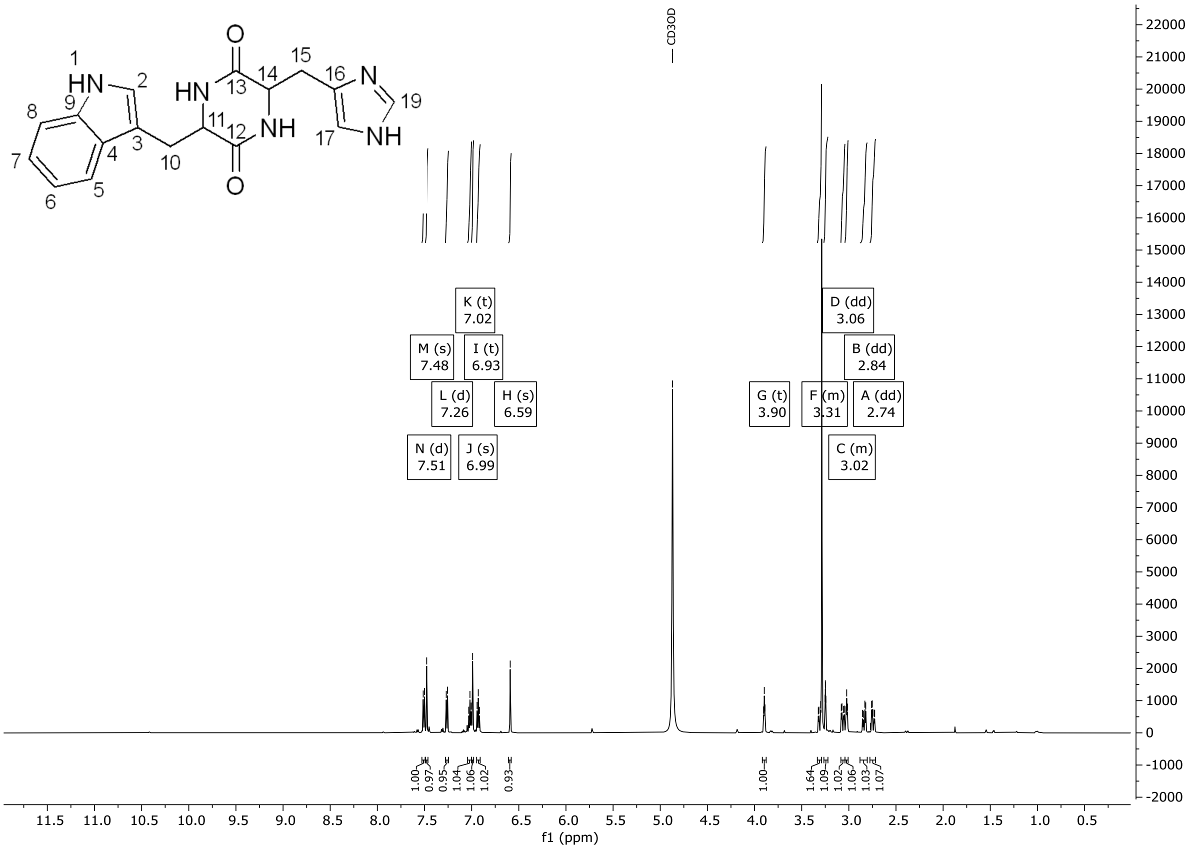

Figure S35: ^{1}H-NMR spectrum of LD-cWH **8c** in MeOD (600 MHz).

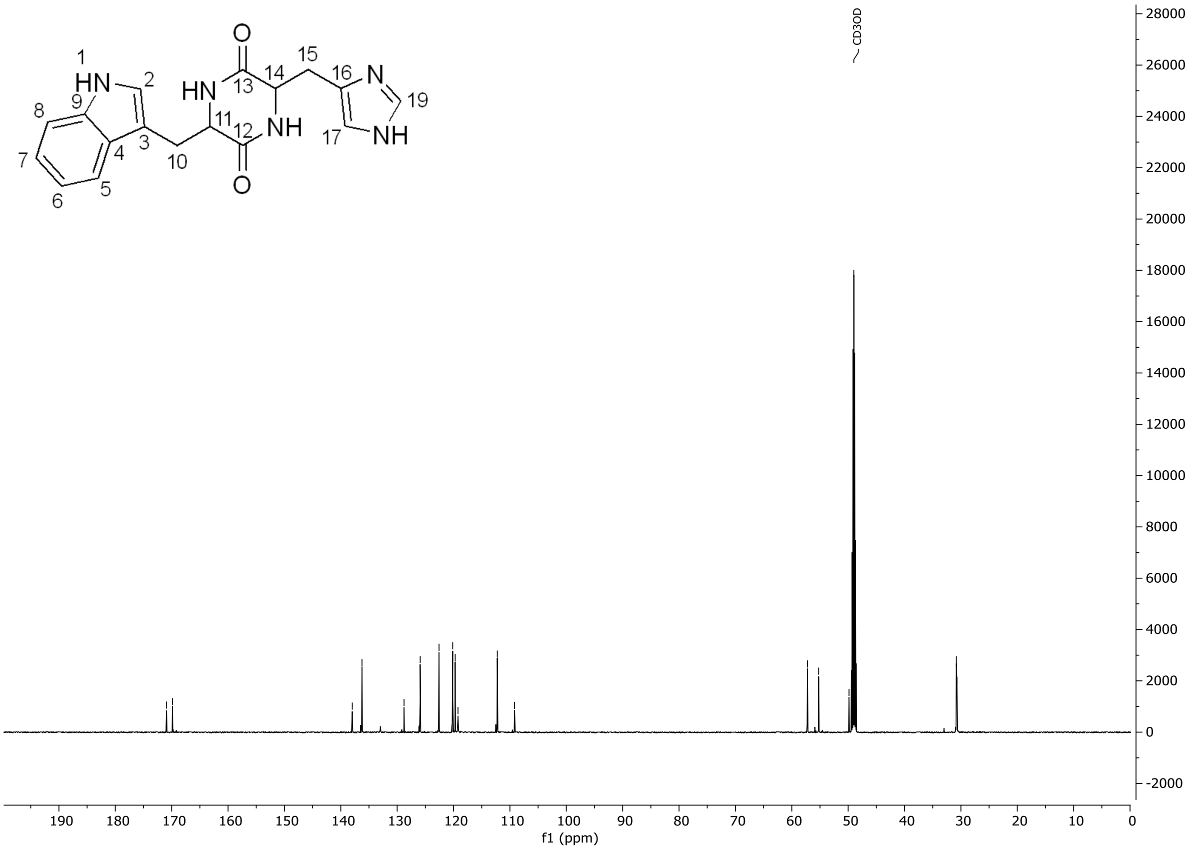

Figure S36: ^{13}C-NMR spectrum of LD-cWH **8c** in MeOD (151 MHz).

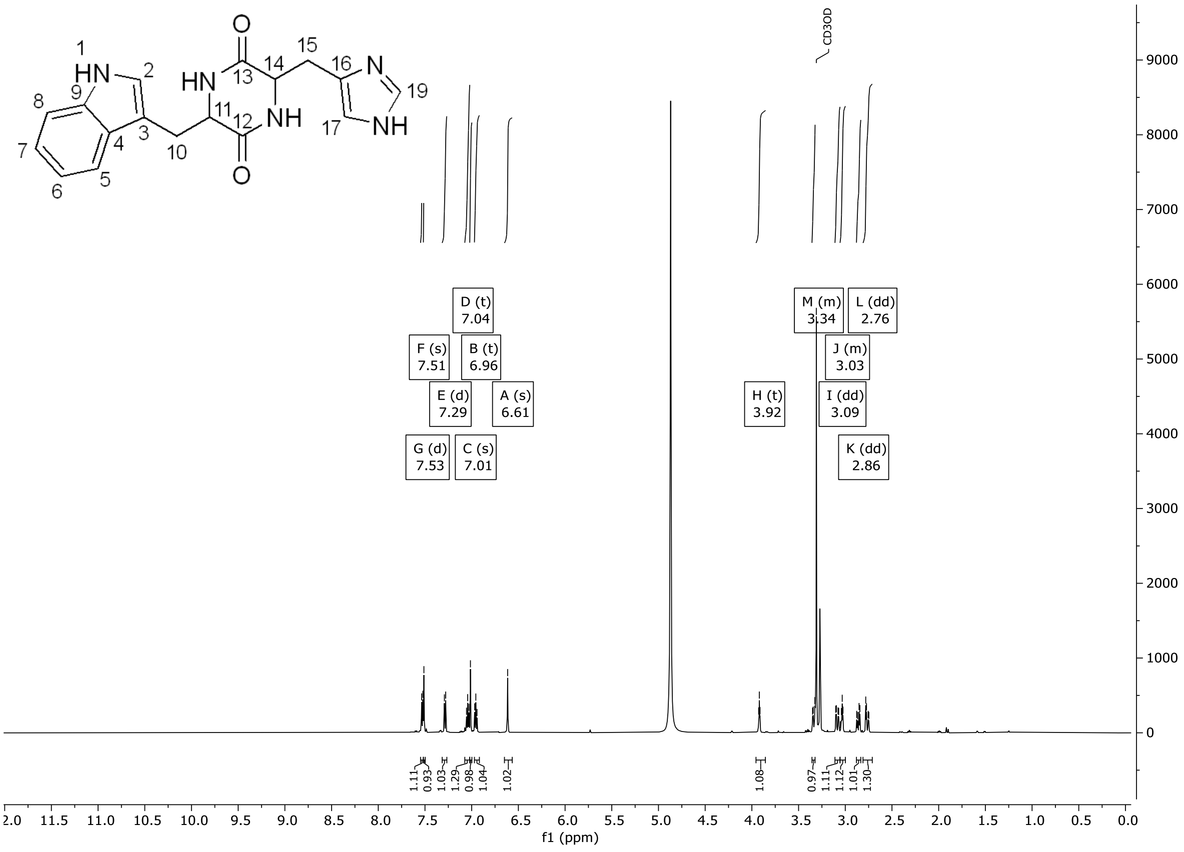

*Figure S37: ^{1}H-NMR spectrum of DL-cWH **8d** in MeOD (600 MHz).*

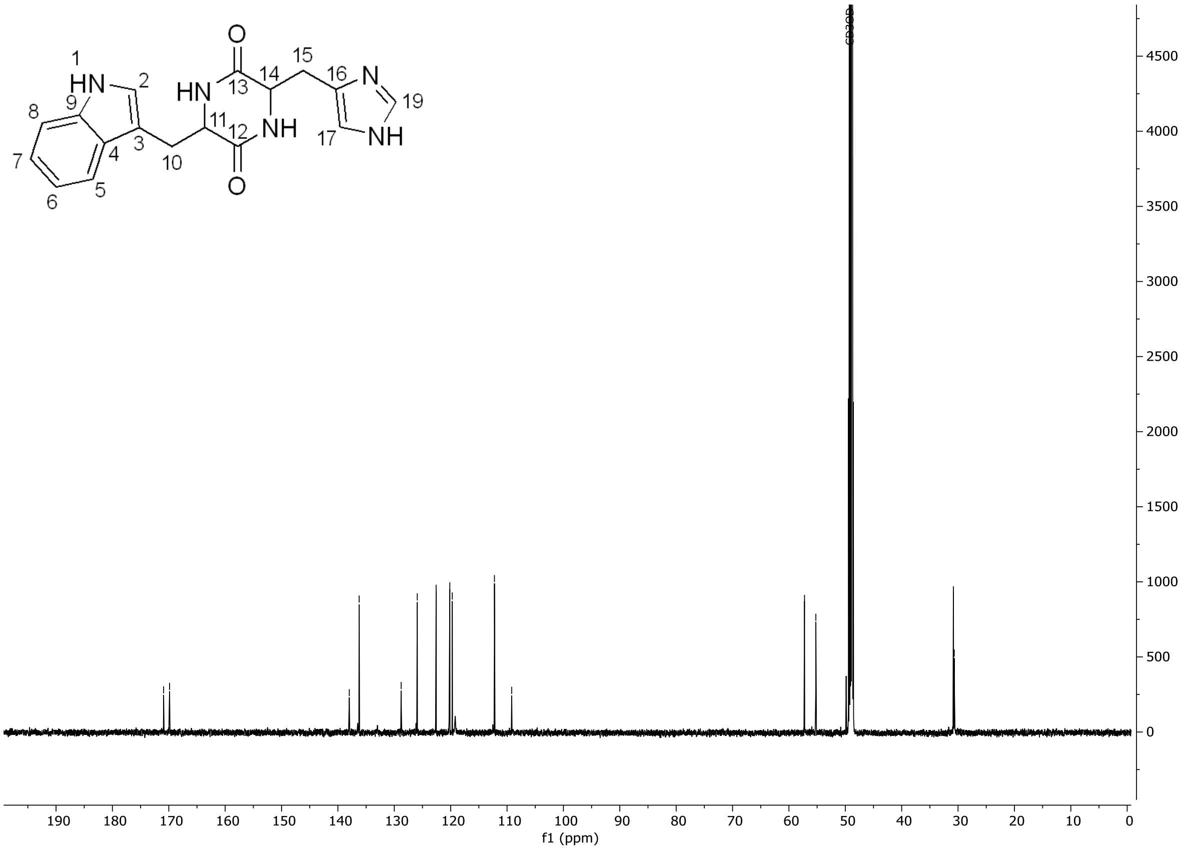

*Figure S38: ^{13}C-NMR spectrum of DL-cWH **8d** in MeOD (151 MHz).*

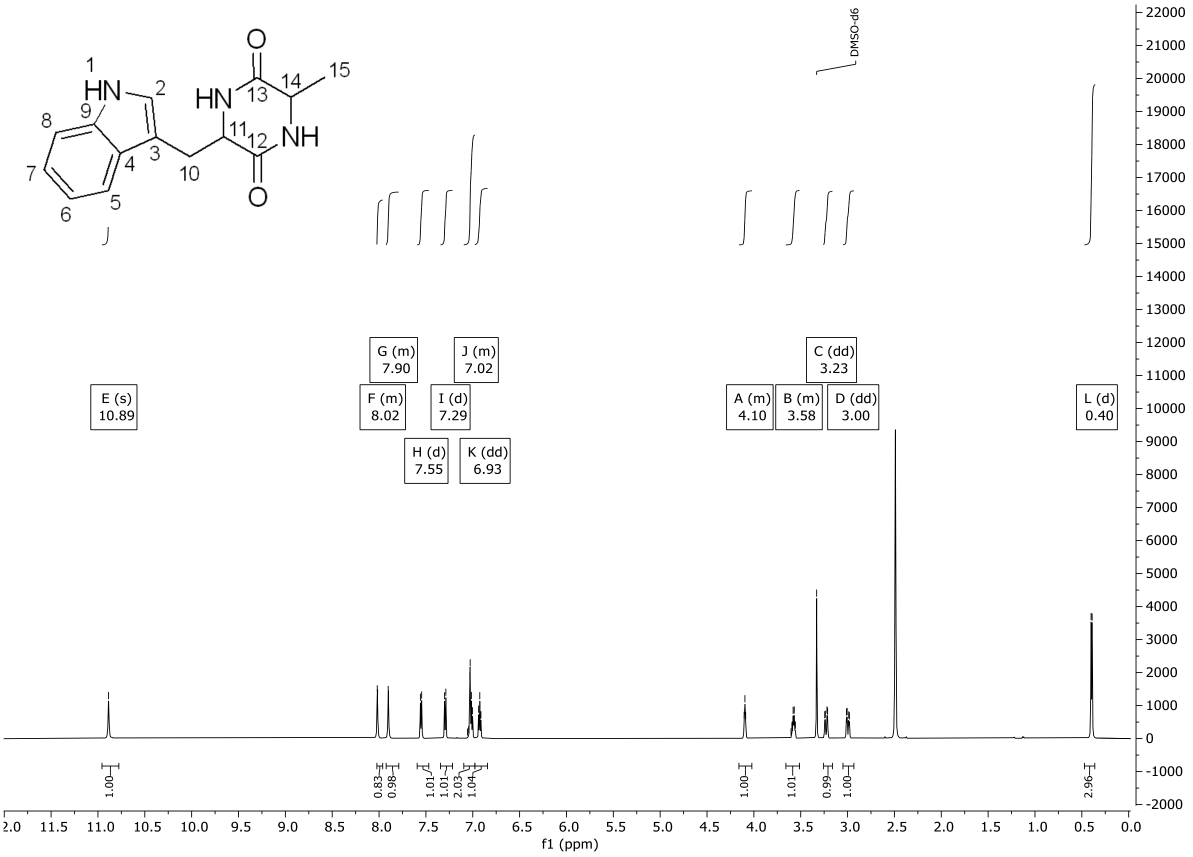

Figure S39:[1]*H-NMR spectrum of LL-cWA **9** in DMSO (600 MHz).*

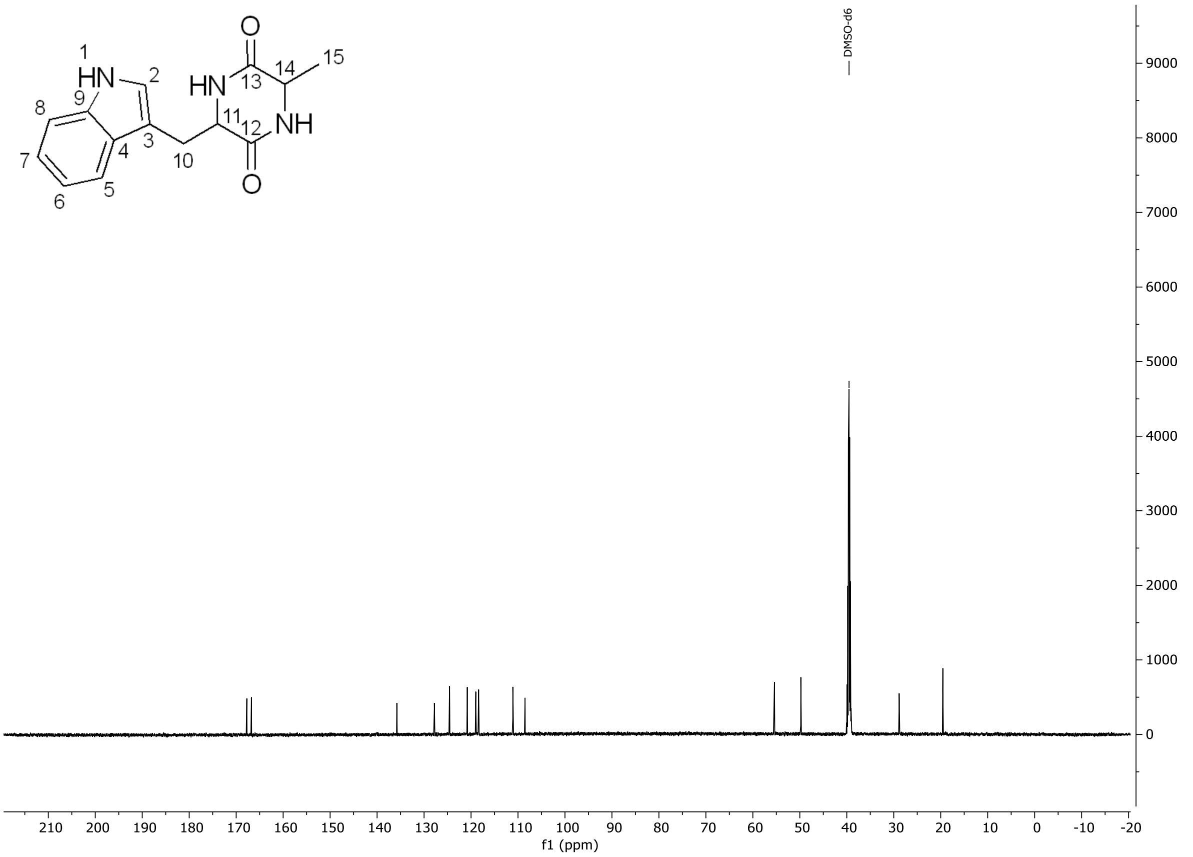

Figure S40: ^{13}*C-NMR spectrum of LL-cWA **9** in DMSO (151 MHz).*

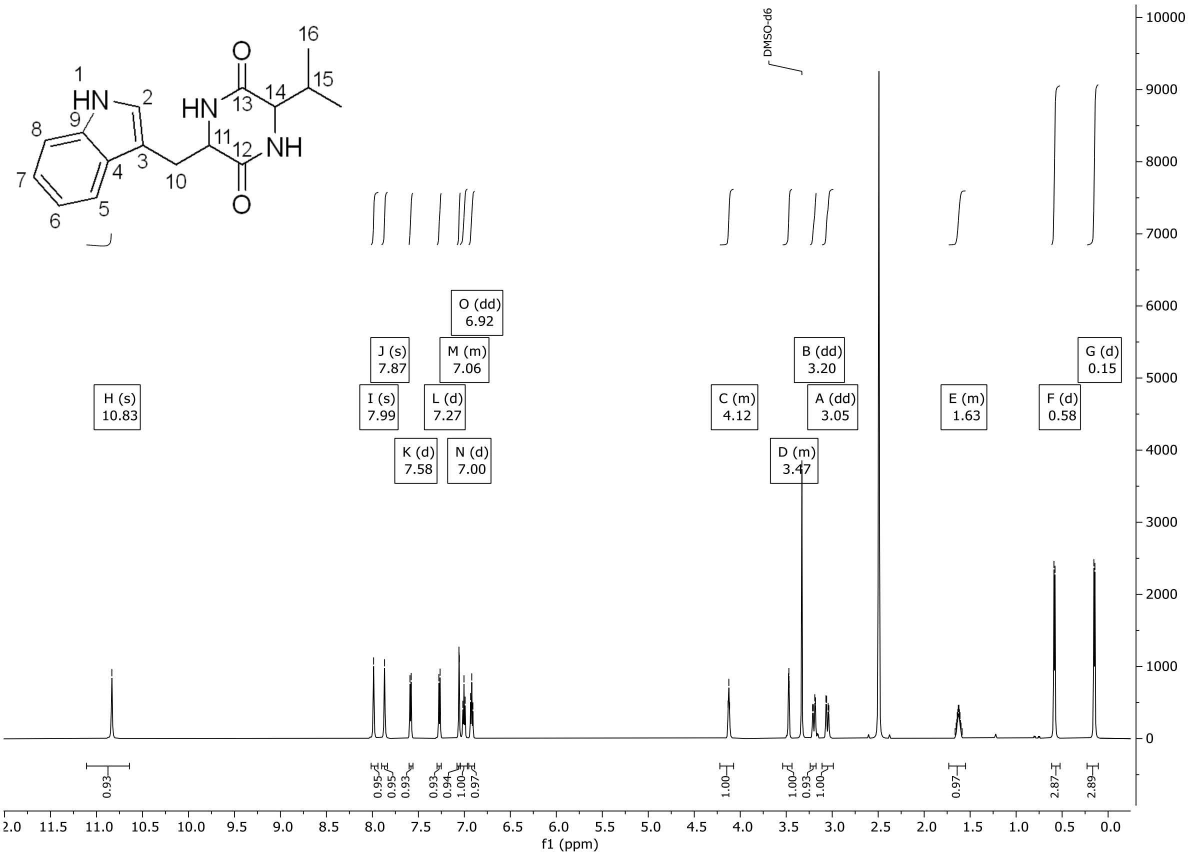

*Figure S41: ^{1}H-NMR spectrum of LL-cWV **10** in DMSO (600 MHz).*

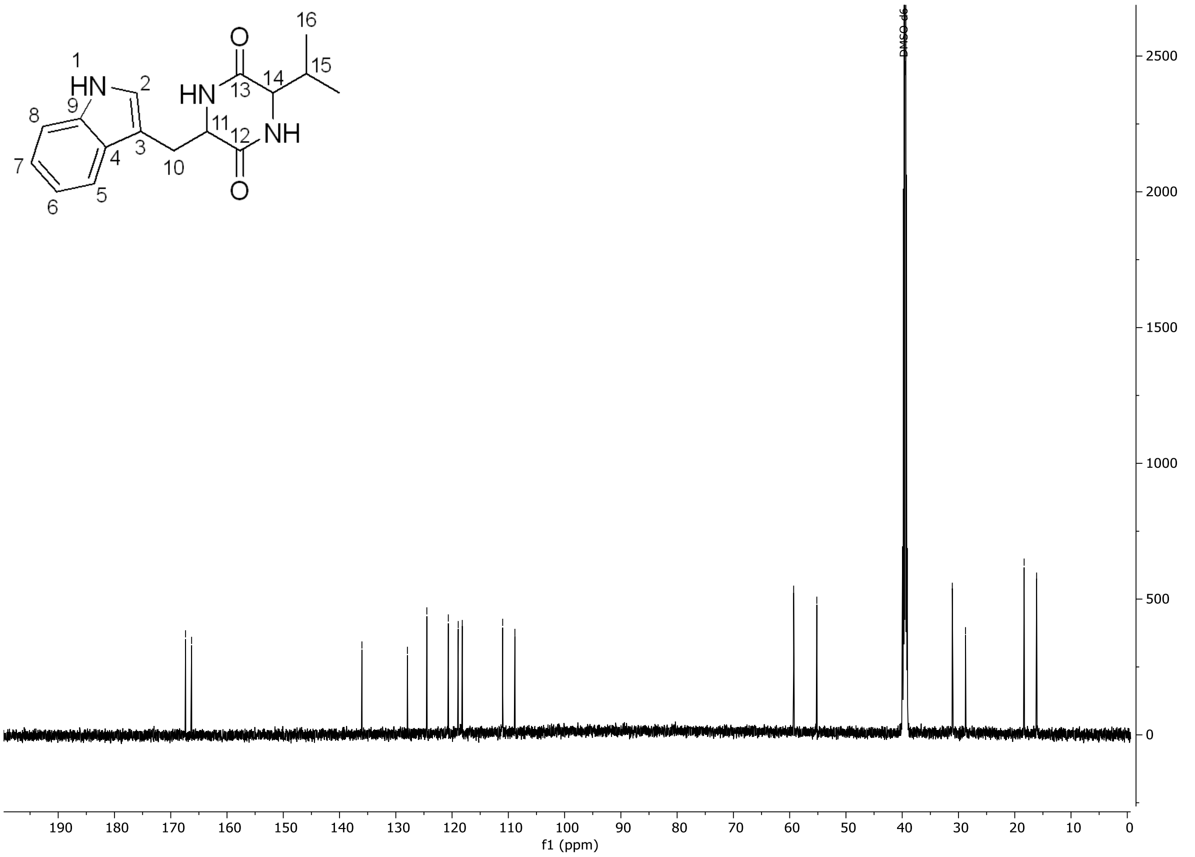

*Figure S42: ^{13}C-NMR spectrum of LL-cWV **10** in DMSO (151 MHz).*

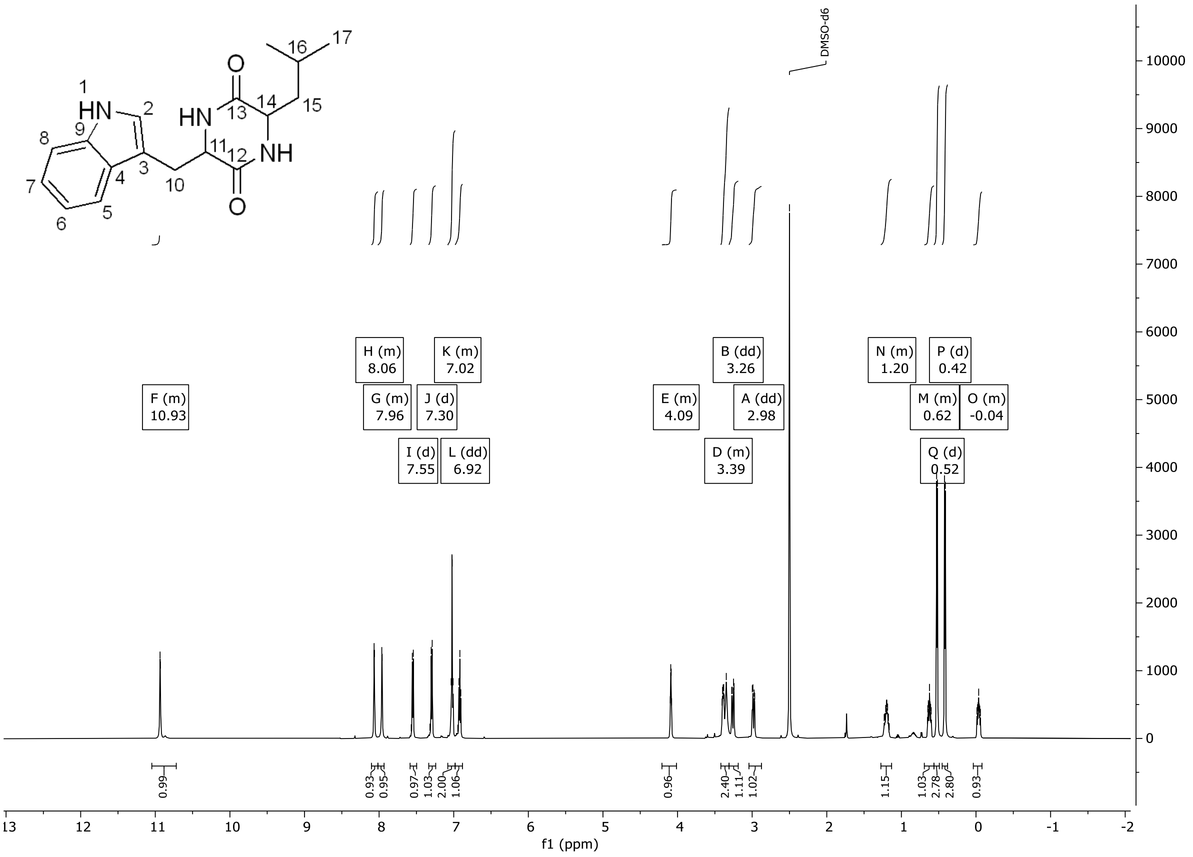

*Figure S43: ^{1}H-NMR spectrum of LL-cWL **11** in DMSO (600 MHz).*

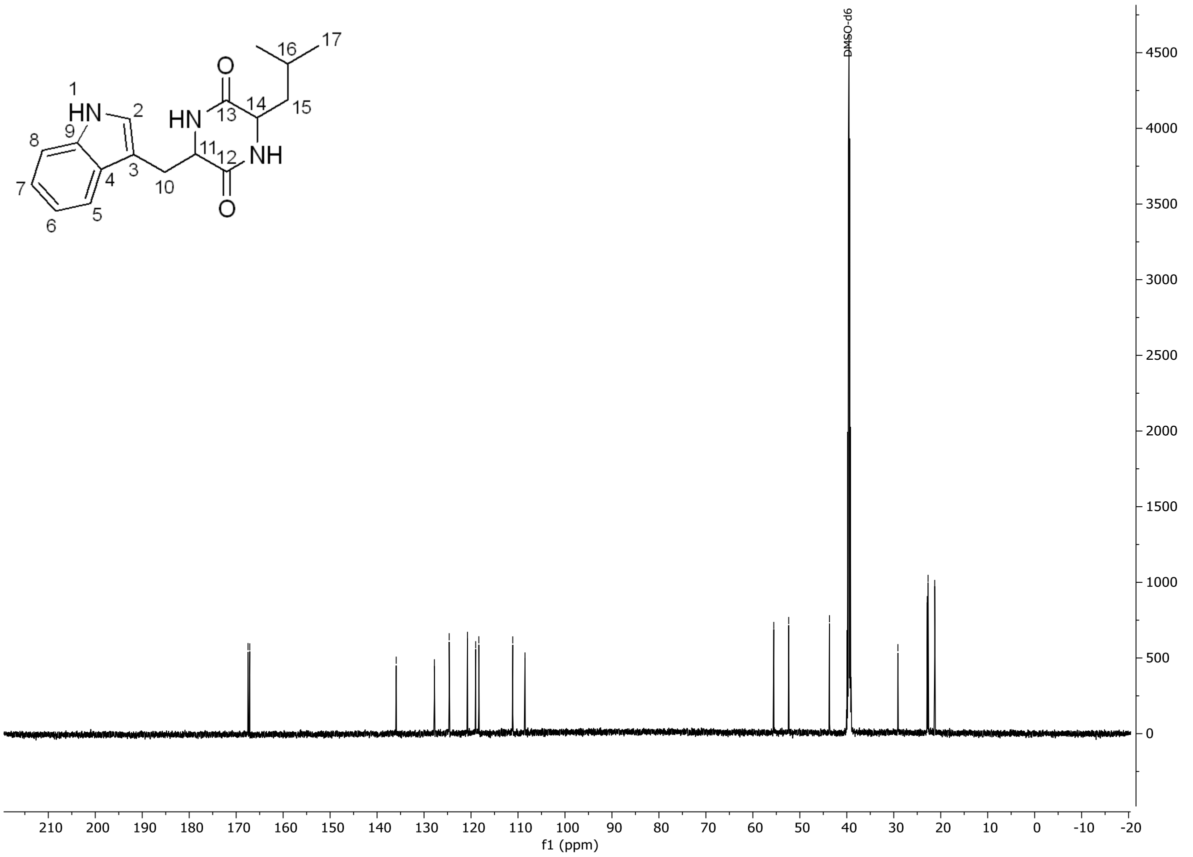

*Figure S44: ^{13}C-NMR spectrum of LL-cWL **11** in DMSO (151 MHz).*

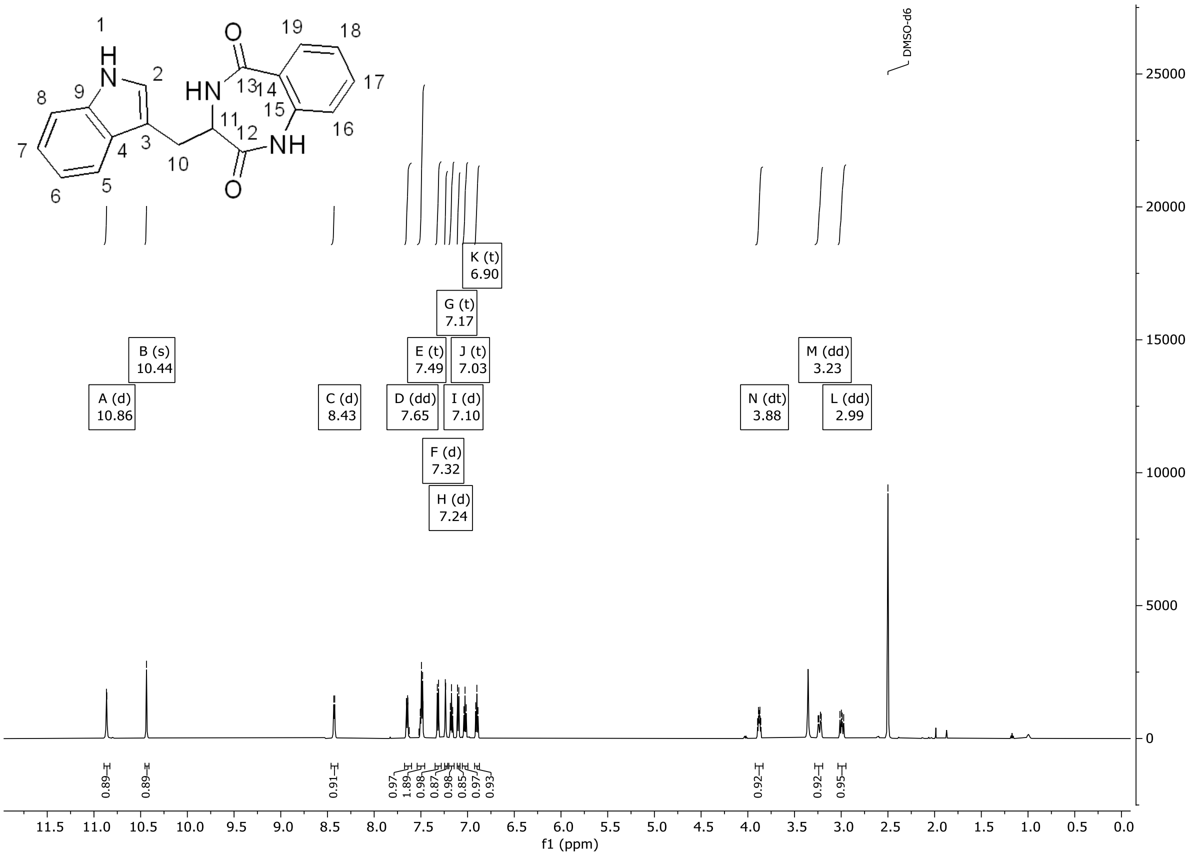

*Figure S45: ^{1}H-NMR spectrum of L-cWBenz **12a** in DMSO (600 MHz).*

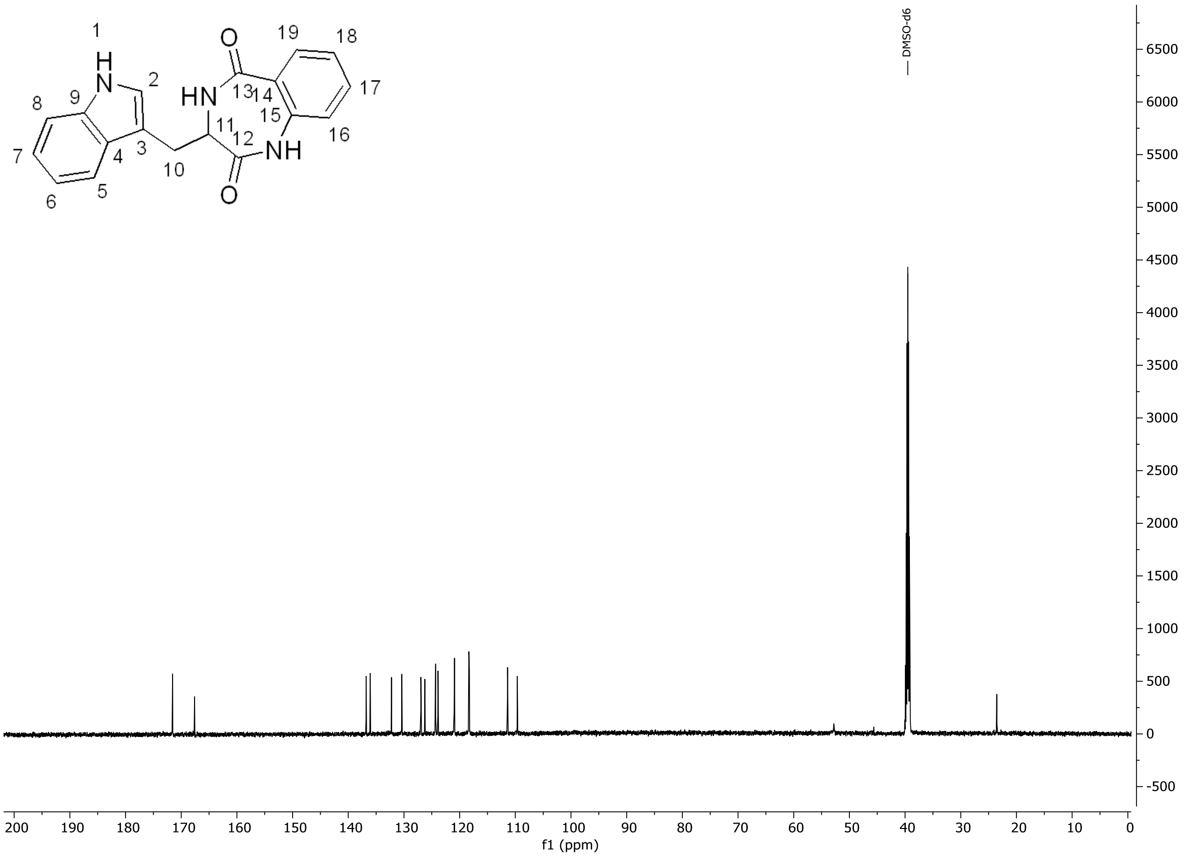

*Figure S46: ^{13}C-NMR spectrum of L-cWBenz **12a** in DMSO (151 MHz).*

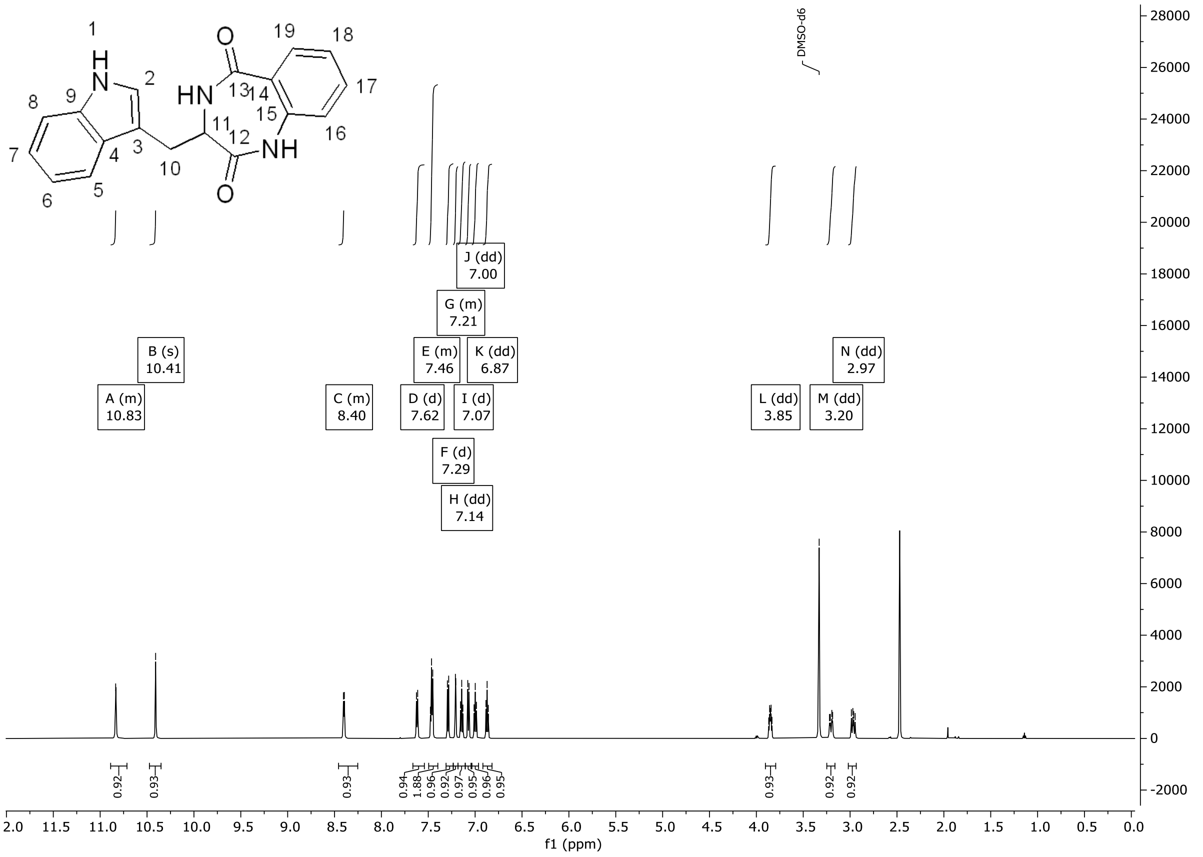

*Figure S47: ¹H-NMR spectrum of D-cWBenz **12b** in DMSO (600 MHz).*

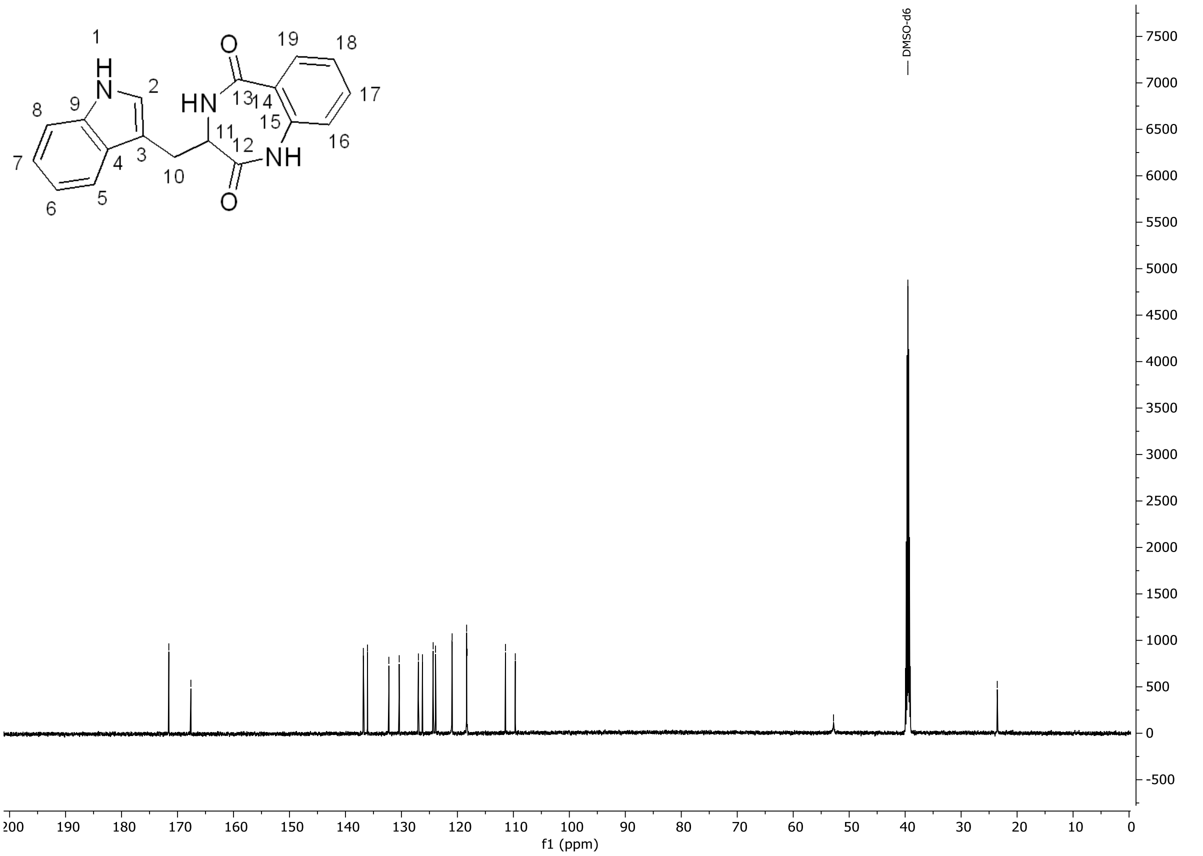

*Figure S48: ¹³C-NMR spectrum of D-cWBenz **12b** in DMSO (151 MHz).*

Literature

1. M. Haase, B. David, B. Paschold, T. Classen, P. Schneider, N. Pozhydaieva, H. Gohlke and J. Pietruszka, Application of the C3-Methyltransferase StspM1 for the Synthesis of the Natural Pyrroloindole Motif, *ACS Catal.*, 2023, **14**, 227-236.
2. J. Jumper, R. Evans, A. Pritzel, T. Green, M. Figurnov, O. Ronneberger, K. Tunyasuvunakool, R. Bates, A. Žídek, A. Potapenko, A. Bridgland, C. Meyer, S. A. A. Kohl, A. J. Ballard, A. Cowie, B. Romera-Paredes, S. Nikolov, R. Jain, J. Adler, T. Back, S. Petersen, D. Reiman, E. Clancy, M. Zielinski, M. Steinegger, M. Pacholska, T. Berghammer, S. Bodenstein, D. Silver, O. Vinyals, A. W. Senior, K. Kavukcuoglu, P. Kohli and D. Hassabis, Highly accurate protein structure prediction with AlphaFold, *Nature*, 2021, **596**, 583-589.
3. R. Evans, M. O'Neill, A. Pritzel, N. Antropova, A. Senior, T. Green, A. Žídek, R. Bates, S. Blackwell, J. Yim, O. Ronneberger, S. Bodenstein, M. Zielinski, A. Bridgland, A. Potapenko, A. Cowie, K. Tunyasuvunakool, R. Jain, E. Clancy, P. Kohli, J. Jumper and D. Hassabis, Protein complex prediction with AlphaFold-Multimer, *bioRxiv*, 2022, DOI: 10.1101/2021.10.04.463034, 2021.2010.2004.463034.
4. M. Mirdita, K. Schütze, Y. Moriwaki, L. Heo, S. Ovchinnikov and M. Steinegger, ColabFold: making protein folding accessible to all, *Nat. Methods*, 2022, **19**, 679-682.
5. R. A. Friesner, J. L. Banks, R. B. Murphy, T. A. Halgren, J. J. Klicic, D. T. Mainz, M. P. Repasky, E. H. Knoll, M. Shelley, J. K. Perry, D. E. Shaw, P. Francis and P. S. Shenkin, Glide: a new approach for rapid, accurate docking and scoring. 1. Method and assessment of docking accuracy, *J. Med. Chem.*, 2004, **47**, 1739-1749.
6. R. A. Friesner, R. B. Murphy, M. P. Repasky, L. L. Frye, J. R. Greenwood, T. A. Halgren, P. C. Sanschagrin and D. T. Mainz, Extra precision glide: docking and scoring incorporating a model of hydrophobic enclosure for protein-ligand complexes, *J. Med. Chem.*, 2006, **49**, 6177-6196.
7. E. Harder, W. Damm, J. Maple, C. Wu, M. Reboul, J. Y. Xiang, L. Wang, D. Lupyan, M. K. Dahlgren, J. L. Knight, J. W. Kaus, D. S. Cerutti, G. Krilov, W. L. Jorgensen, R. Abel and R. A. Friesner, OPLS3: A Force Field Providing Broad Coverage of Drug-like Small Molecules and Proteins, *J. Chem. Theory Comput.*, 2016, **12**, 281-296.
8. M. H. Olsson, C. R. Sondergaard, M. Rostkowski and J. H. Jensen, PROPKA3: Consistent Treatment of Internal and Surface Residues in Empirical pKa Predictions, *J. Chem. Theory Comput.*, 2011, **7**, 525-537.
9. G. M. Sastry, M. Adzhigirey, T. Day, R. Annabhimoju and W. Sherman, Protein and ligand preparation: parameters, protocols, and influence on virtual screening enrichments, *J. Comput. Aided Mol. Des.*, 2013, **27**, 221-234.
10. R. C. Johnston, K. Yao, Z. Kaplan, M. Chelliah, K. Leswing, S. Seekins, S. Watts, D. Calkins, J. Chief Elk, S. V. Jerome, M. P. Repasky and J. C. Shelley, Epik: pK(a) and Protonation State Prediction through Machine Learning, *J. Chem. Theory Comput.*, 2023, **19**, 2380-2388.
11. J. Wang, P. Cieplak and P. A. Kollman, How well does a restrained electrostatic potential (RESP) model perform in calculating conformational energies of organic and biological molecules?, *J. Comput. Chem.*, 2000, **21**, 1049-1074.
12. T. Lu and F. Chen, Multiwfn: a multifunctional wavefunction analyzer, *J. Comput. Chem.*, 2012, **33**, 580-592.
13. F. Neese, F. Wennmohs, U. Becker and C. Riplinger, The ORCA quantum chemistry program package, *J. Chem. Phys.*, 2020, **152**, 224108.
14. J. Wang, R. M. Wolf, J. W. Caldwell, P. A. Kollman and D. A. Case, Development and testing of a general amber force field, *J. Comput. Chem.*, 2004, **25**, 1157-1174.
15. J. Wang, W. Wang, P. A. Kollman and D. A. Case, Automatic atom type and bond type perception in molecular mechanical calculations, *J. Mol. Graph Model*, 2006, **25**, 247-260.

16. D. A. Case, H. M. Aktulga, K. Belfon, D. S. Cerutti, G. A. Cisneros, V. W. D. Cruzeiro, N. Forouzesh, T. J. Giese, A. W. Gotz, H. Gohlke, S. Izadi, K. Kasavajhala, M. C. Kaymak, E. King, T. Kurtzman, T. S. Lee, P. Li, J. Liu, T. Luchko, R. Luo, M. Manathunga, M. R. Machado, H. M. Nguyen, K. A. O'Hearn, A. V. Onufriev, F. Pan, S. Pantano, R. Qi, A. Rahnamoun, A. Risheh, S. Schott-Verdugo, A. Shajan, J. Swails, J. Wang, H. Wei, X. Wu, Y. Wu, S. Zhang, S. Zhao, Q. Zhu, T. E. Cheatham, 3rd, D. R. Roe, A. Roitberg, C. Simmerling, D. M. York, M. C. Nagan and K. M. Merz, Jr., AmberTools, *J. Chem. Inf. Model*, 2023, **63**, 6183-6191.
17. J. A. Maier, C. Martinez, K. Kasavajhala, L. Wickstrom, K. E. Hauser and C. Simmerling, ff14SB: Improving the Accuracy of Protein Side Chain and Backbone Parameters from ff99SB, *J. Chem. Theory Comput.*, 2015, **11**, 3696-3713.
18. W. L. Jorgensen, J. Chandrasekhar, J. D. Madura, R. W. Impey and M. L. Klein, Comparison of simple potential functions for simulating liquid water, *J. Chem. Phys.*, 1983, **79**, 926-935.
19. T. Darden, D. York and L. Pedersen, Particle mesh Ewald: An N·log(N) method for Ewald sums in large systems, *J. Chem. Phys.*, 1993, **98**, 10089-10092.
20. D. A. Case, H. M. Aktulga, K. Belfon, D. S. Cerutti, G. A. Cisneros, V. W. D. Cruzeiro, N. Forouzesh, T. J. Giese, A. W. Götz, H. Gohlke, S. Izadi, K. Kasavajhala, M. C. Kaymak, E. King, T. Kurtzman, T.-S. Lee, P. Li, J. Liu, T. Luchko, R. Luo, M. Manathunga, M. R. Machado, H. M. Nguyen, K. A. O’Hearn, A. V. Onufriev, F. Pan, S. Pantano, R. Qi, A. Rahnamoun, A. Risheh, S. Schott-Verdugo, A. Shajan, J. Swails, J. Wang, H. Wei, X. Wu, Y. Wu, S. Zhang, S. Zhao, Q. Zhu, T. E. Cheatham, III, D. R. Roe, A. Roitberg, C. Simmerling, D. M. York, M. C. Nagan and K. M. Merz, Jr., AmberTools, *J. Chem. Inf. Model.*, 2023, **63**, 6183-6191.
21. R. Salomon-Ferrer, A. W. Gotz, D. Poole, S. Le Grand and R. C. Walker, Routine Microsecond Molecular Dynamics Simulations with AMBER on GPUs. 2. Explicit Solvent Particle Mesh Ewald, *J. Chem. Theory Comput.*, 2013, **9**, 3878-3888.
22. J.-P. Ryckaert, G. Ciccotti and H. J. C. Berendsen, Numerical integration of the cartesian equations of motion of a system with constraints: molecular dynamics of n-alkanes, *J. Comput. Phys.*, 1977, **23**, 327-341.
23. D. R. Roe and T. E. Cheatham, 3rd, PTRAJ and CPPTRAJ: Software for Processing and Analysis of Molecular Dynamics Trajectory Data, *J. Chem. Theory Comput.*, 2013, **9**, 3084-3095.
24. B. E. Suzek, Y. Wang, H. Huang, P. B. McGarvey, C. H. Wu and t. U. Consortium, UniRef clusters: a comprehensive and scalable alternative for improving sequence similarity searches, *Bioinformatics*, 2014, **31**, 926-932.
25. B. E. Suzek, H. Huang, P. McGarvey, R. Mazumder and C. H. Wu, UniRef: comprehensive and non-redundant UniProt reference clusters, *Bioinformatics*, 2007, **23**, 1282-1288.
26. P. Cunningham, Biological Sequence Analysis. Probabilistic Models of Proteins and Nucleic Acids. R. Durbin, S. Eddy, A. Krogh and G. Mitchison, *Cell Biochem. Funct.*, 1999, **17**, 73-73.
27. F. Glaser, T. Pupko, I. Paz, R. E. Bell, D. Bechor-Shental, E. Martz and N. Ben-Tal, ConSurf: identification of functional regions in proteins by surface-mapping of phylogenetic information, *Bioinformatics*, 2003, **19**, 163-164.
28. M. Landau, I. Mayrose, Y. Rosenberg, F. Glaser, E. Martz, T. Pupko and N. Ben-Tal, ConSurf 2005: the projection of evolutionary conservation scores of residues on protein structures, *Nucleic Acids Res.*, 2005, **33**, W299-302.
29. G. Celniker, G. Nimrod, H. Ashkenazy, F. Glaser, E. Martz, I. Mayrose, T. Pupko and N. Ben-Tal, ConSurf: Using Evolutionary Data to Raise Testable Hypotheses about Protein Function, *Isr. J. Chem.*, 2013, **53**, 199-206.
30. H. Ashkenazy, S. Abadi, E. Martz, O. Chay, I. Mayrose, T. Pupko and N. Ben-Tal, ConSurf 2016: an improved methodology to estimate and visualize evolutionary conservation in macromolecules, *Nucleic Acids Res.*, 2016, **44**, W344-350.
31. B. Yariv, E. Yariv, A. Kessel, G. Masrati, A. B. Chorin, E. Martz, I. Mayrose, T. Pupko and N. Ben-Tal, Using evolutionary data to make sense of macromolecules with a "face-lifted" ConSurf, *Protein Sci*, 2023, **32**, e4582.

32. M. Steinegger and J. Soding, MMseqs2 enables sensitive protein sequence searching for the analysis of massive data sets, *Nat. Biotechnol.*, 2017, **35**, 1026-1028.
33. M. Mirdita, K. Schutze, Y. Moriwaki, L. Heo, S. Ovchinnikov and M. Steinegger, ColabFold: making protein folding accessible to all, *Nat. Methods*, 2022, **19**, 679-682.
34. R. C. Edgar, Muscle5: High-accuracy alignment ensembles enable unbiased assessments of sequence homology and phylogeny, *Nat. Commun.*, 2022, **13**, 6968.
35. T. D. Schneider and R. M. Stephens, Sequence logos: a new way to display consensus sequences, *Nucleic Acids Res.*, 1990, **18**, 6097-6100.
36. G. E. Crooks, G. Hon, J. M. Chandonia and S. E. Brenner, WebLogo: a sequence logo generator, *Genome Res*, 2004, **14**, 1188-1190.
37. W. Kabsch, XDS, *Acta Crystallogr. D Biol. Crystallogr.*, 2010, **66**, 125-132.
38. A. Vagin and A. Teplyakov, Molecular replacement with MOLREP, *Acta Crystallogr. D Biol. Crystallogr.*, 2010, **66**, 22-25.
39. T. C. Terwilliger, R. W. Grosse-Kunstleve, P. V. Afonine, N. W. Moriarty, P. H. Zwart, L.-W. Hung, R. J. Read and P. D. Adams, Iterative model building, structure refinement and density modification with the PHENIX AutoBuild wizard, *Acta Crystallogr. D Biol. Crystallogr.*, 2008, **64**, 61-69.
40. D. Liebschner, P. V. Afonine, M. L. Baker, G. Bunkóczi, V. B. Chen, T. I. Croll, B. Hintze, L. W. Hung, S. Jain, A. J. McCoy, N. W. Moriarty, R. D. Oeffner, B. K. Poon, M. G. Prisant, R. J. Read, J. S. Richardson, D. C. Richardson, M. D. Sammito, O. V. Sobolev, D. H. Stockwell, T. C. Terwilliger, A. G. Urzhumtsev, L. L. Videau, C. J. Williams and P. D. Adams, Macromolecular structure determination using X-rays, neutrons and electrons: recent developments in Phenix, *Acta Crystallogr. D Struct. Biol.*, 2019, **75**, 861-877.
41. P. Emsley, B. Lohkamp, W. G. Scott and K. Cowtan, Features and development of Coot, *Acta Crystallogr. D Biol. Crystallogr.*, 2010, **66**, 486-501.
42. C. J. Williams, J. J. Headd, N. W. Moriarty, M. G. Prisant, L. L. Videau, L. N. Deis, V. Verma, D. A. Keedy, B. J. Hintze, V. B. Chen, S. Jain, S. M. Lewis, W. B. Arendall III, J. Snoeyink, P. D. Adams, S. C. Lovell, J. S. Richardson and D. C. Richardson, MolProbity: More and better reference data for improved all-atom structure validation, *Protein Sci.*, 2018, **27**, 293-315.
43. I. Cacciatore, A. Cocco, M. Costa, M. Fontana, G. Lucente, L. Pecci and F. Pinnen, Biochemical properties of new synthetic carnosine analogues containing the residue of 2,3-diaminopropionic acid: the effect of N-acetylation, *Amino Acids*, 2005, **28**, 77-83.
44. P.-Q. Huang, Y. Wang, S.-P. Luo, H. Geng, Y.-P. Ruan and A.-E. Wang, Procedure—economical enantioselective total syntheses of asperlicins C and E, *Tetrahedron Lett.*, 2015, **56**, 1255-1258.
45. S. Nishanth Kumar, C. Mohandas and B. Nambisan, Purification, structural elucidation and bioactivity of tryptophan containing diketopiperazines, from Comamonas testosteroni associated with a rhabditid entomopathogenic nematode against major human-pathogenic bacteria, *Peptides*, 2014, **53**, 48-58.
46. C. Pérez-Balado and Á. R. de Lera, Concise total synthesis and structural revision of (+)-pestalazine B, *Org. Biomol. Chem.*, 2010, **8**, 5179-5186.
47. B. N. S. Ningsih, V. Rukachaisirikul, S. Phongpaichit, S. Preedanon, J. Sakayaroj and C. Muanprasat, A nonadride derivative from the marine-derived fungus Aspergillus chevalieri PSU-AMF79, *Nat. Prod. Res.*, 2023, **37**, 2311-2318.
48. H. Ueda, S. Sato, K. Noda, H. Hakamata, E. Kwon, N. Kobayashi and H. Tokuyama, Iron-Catalyzed Biomimetic Dimerization of Tryptophan-Containing Peptides, *Angew. Chem. Int. Ed.*, 2023, **62**, e202302404.
49. S. M. Anil, R. Shobith, K. R. Kiran, T. R. Swaroop, N. Mallesha and M. P. Sadashiva, Facile synthesis of 1,4-benzodiazepine-2,5-diones and quinazolinones from amino acids as anti-tubercular agents, *New J. Chem.*, 2019, **43**, 182-187.
